# In vivo secondary structural analysis of Influenza A virus genomic RNA

**DOI:** 10.1007/s00018-023-04764-1

**Published:** 2023-05-02

**Authors:** Barbara Mirska, Tomasz Woźniak, Dagny Lorent, Agnieszka Ruszkowska, Jake M. Peterson, Walter N. Moss, David H. Mathews, Ryszard Kierzek, Elzbieta Kierzek

**Affiliations:** 1 Institute of Bioorganic Chemistry, Polish Academy of Sciences, Noskowskiego 12/14, 61-704 Poznan, Poland; 2 Institute of Human Genetics, Polish Academy of Sciences, Strzeszynska 32, 60-479 Poznan, Poland; 3 Roy J. Carver Department of Biophysics, Biochemistry and Molecular Biology, Iowa State University, Ames, IA 50011 USA; 4 Department of Biochemistry & Biophysics and Center for RNA Biology, School of Medicine and Dentistry, University of Rochester, 601 Elmwood Avenue, Box 712, Rochester, NY 14642 USA

**Keywords:** Next-generation sequencing (NGS), Influenza A virus (IAV), Mutational profiling (MaP), RNA secondary structure, Conserved RNA motifs, Chemical mapping

## Abstract

Influenza A virus (IAV) is a respiratory virus that causes epidemics and pandemics. Knowledge of IAV RNA secondary structure in vivo is crucial for a better understanding of virus biology. Moreover, it is a fundament for the development of new RNA-targeting antivirals. Chemical RNA mapping using selective 2’-hydroxyl acylation analyzed by primer extension (SHAPE) coupled with Mutational Profiling (MaP) allows for the thorough examination of secondary structures in low-abundance RNAs in their biological context. So far, the method has been used for analyzing the RNA secondary structures of several viruses including SARS-CoV-2 in virio and in cellulo. Here, we used SHAPE-MaP and dimethyl sulfate mutational profiling with sequencing (DMS-MaPseq) for genome-wide secondary structure analysis of viral RNA (vRNA) of the pandemic influenza A/California/04/2009 (H1N1) strain in both in virio and in cellulo environments. Experimental data allowed the prediction of the secondary structures of all eight vRNA segments in virio and, for the first time, the structures of vRNA5, 7, and 8 in cellulo. We conducted a comprehensive structural analysis of the proposed vRNA structures to reveal the motifs predicted with the highest accuracy. We also performed a base-pairs conservation analysis of the predicted vRNA structures and revealed many highly conserved vRNA motifs among the IAVs. The structural motifs presented herein are potential candidates for new IAV antiviral strategies.

**Supplementary Information:**

The online version contains supplementary material available at 10.1007/s00018-023-04764-1.

## Introduction

Of all the viral threats to humankind, those with RNA genomes are considered particularly severe [1–3]. Among these are zoonotic single-stranded RNA (ssRNA) viruses, which are considered to have the highest pandemic potential [2, 4]. Indeed, the last IAV pandemic in 2009–2010 (H1N1 strain A/California/04/2009), as well as the recent coronavirus pandemic (2019–present day), showed that world public health and daily life could be highly affected by RNA viruses [5, 6]. Significantly, these pandemics could not be avoided despite both *Orthomyxoviridae* and *Coronaviridae* families having been identified many times prior as having pandemic potential [7–10].

RNA genomes allow for relatively fast but error-prone replication cycles [11]. This leads to a high mutation rate within RNA viruses that enables them to evolve and outpace current antiviral strategies [12]. Mutations to high-resistance antiviral strains could lead to an epidemic with unpredictable consequences [13]. Additionally, the effectiveness of vaccines varies between seasons and can often provide only minor protection against novel strains [14]. Current protein-targeting antivirals can be replaced with promising drug candidates that target RNA. Thus, multidimensional research concerning the biology of ssRNA viruses is crucial, as improved knowledge contributes to the design of more effective and targeted antiviral therapies.

The IAV genome consists of eight single-stranded, negative-sense viral RNAs (vRNAs), which combine with proteins to form viral ribonucleoprotein (vRNP) complexes [15]. Each vRNP complex comprises a single vRNA molecule interacting with many copies of nucleoproteins (NP) and three proteins forming a viral RNA-dependent RNA polymerase (RdRp) complex: PB2, PB1, and PA. During viral replication and transcription, each vRNP forms into independent functional units [16]. Research concerning RNA-RNA interactions inside the viral genome revealed a complicated interaction network [17, 18]. During the replication cycle of influenza, vRNAs communicate with each other via regions called packaging signals, allowing the accurate assembly and packaging of vRNPs into progeny virions [19]. RNA secondary structures are also proposed to play important roles during the viral life cycle including RNA transcription, replication and the transition between them, as well as interactions with the host cellular machinery [20–22]. The RNA motifs, often highly conserved, provide great antivirals for RNA-specific targeting across distant IAV strains.

The strategy of influenza inhibition using antisense oligonucleotides (ASOs), based on the secondary structures determined in vitro, was successfully applied in cellulo by our group [23–25]. We showed that the effectiveness of RNA-targeting inhibitors might be affected by RNA secondary structure and accessibility during the replication cycle. As of today, the in virio secondary structures of only one H1N1 strain A/WSN/33 have been proposed [18]. However, this research focused mainly on vRNA-vRNA interactions without considering the evolutionary conservation of vRNA secondary structures. Furthermore, genome-wide analysis of the in cellulo RNA structure has not yet been addressed. To fill this gap in the research, we performed both in virio and in cellulo vRNAs structure probing of pandemic influenza strain A/California/04/2009 (H1N1). For structure probing we used two approaches integrated with the Next Generation Sequencing (NGS) methodology: **S**elective 2’-**H**ydroxyl **A**cylation analyzed by **P**rimer **E**xtension coupled with **M**utational **P**rofiling (SHAPE-MaP) and **D**imethyl **S**ulfide probing coupled with **M**utational **P**rofiling (DMS-MaPseq) [26–28].

A variety of cell-permeable chemicals are used to chemically modify RNA in vivo. These chemicals can be further divided depending on the modification mechanism used at the single-stranded regions of the RNA. SHAPE reagents like 2-methylnicotinic acid imidazolide (NAI), 1-methyl-7-nitroisatoic anhydride (1M7), and 5-nitroisatoic anhydride (5NIA) are ribose-specific reagents that modify 2’–OH ribose of unpaired nucleotides within single-stranded and conformationally flexible regions of RNA [29]. Another group collects nucleotide-specific reagents that react at the base-pairing faces of unpaired nucleotides, like dimethyl sulfide (DMS) (adenosine and cytosine specific), glyoxal (guanosine specific), and 1-ethyl-3-(3-dimethylaminopropyl) carbodiimide (EDC) (uridine and guanosine specific) that has been recently introduced and successfully tested in cells [28, 30–32]. For RNA probing in cells and in virions, we chose two chemical reagents: NAI and DMS. The chemically modified RNA is further used for reverse transcription (RT) reaction using Mutational Profiling (MaP). MaP methodology uses a modified  RT reaction that facilitates the read-out of chemically probed RNA in vivo [27–29]*.* In the presence of Mn^2+^ ions, reverse transcriptase introduces mutations such as mismatches, deletions, or insertions in the complementary DNA (cDNA) strand in positions complementary to nucleotide modifications introduced by the chemical reagent. After the second strand synthesis, double-stranded DNA is used for library preparation, followed by NGS sequencing on the Illumina platform. The sequencing results of the reagent-treated and reagent-free samples are compared, and the mutational profile is calculated. The MaP is further calculated into chemical reactivities with single-nucleotide resolution [27–29].

Our data allowed us to propose structures of all eight vRNA segments in virio*,* as well as, for the very first time, secondary structures for vRNA segments 5, 7, and 8 in cellulo. Next, we performed a wide-scale structural conservation analysis on tens of thousands of IAV genomes and revealed dozens of structural motifs with high (> 95%) conservation. Moreover, we compared our data with previously established structures, including in vitro and in virio predictions within distant IAV strains [18, 24, 33–37]. We juxtapose low Shannon Entropy, low DMS reactivity, and low SHAPE reactivity regions to establish well-determined structural motifs for each vRNA structure, many of which have high structural conservation that make them ideal candidates for universal inhibitory methods.

## Materials and methods

### Cell culture and virus propagation

The Madin-Darby canine kidney (MDCK) (Merck, ECACC 85011435) cell culture, viral stock propagation of A/California/04/2009 strain (H1N1), and virus titration were prepared as described previously [37]. The original virus stock was a kind gift from prof. Luiz Martinez-Sobrido (Texas Biomedical Research Institute, San Antonio, USA) [38]. The viral titer was calculated with a Focus Forming Assay (FFA) as described in Zmora et al*.* [39]. The Adenocarcinomic human alveolar basal epithelial (A549) (Merck, ECACC 86012804) cell line culture was maintained in Dulbecco’s Modified Eagle’s Medium (DMEM) (Gibco, 10312021) supplemented with 10% heat-inactivated fetal bovine serum (FBS) (Gibco, A5256801), 2 mM glutamine, and antibiotics (100 U/ml penicillin, 100 µg/mL streptomycin) (Gibco, 10378016). The culture was passaged twice a week at 1:5 seeding concentration and incubated at 37 °C with 5% CO_2_ at 95% humidity. The cell cultures were regularly tested to confirm that they were free of Mycoplasma contamination.

### Infection of A549 cells

24 h before infection, A549 0.5 × 10^6^ cells were seeded in 6-well plates. The cells were infected at a multiplicity of infection (MOI) 0.01 with the virus dilution in an infection medium containing 0.3% bovine serum albumin (BSA) (Sigma-Aldrich, A9576), 100 U/mL penicillin, 100 µg/mL streptomycin (Gibco, 15140122), and phosphate-buffered saline (PBS) (BioShop, PBS.415.1). The plates were incubated for 1 h at room temperature with constant rocking. Next, the medium was exchanged with a post-infection medium containing 0.3% BSA, 100 U/mL penicillin, 100 µg/mL streptomycin, 2 mM glutamine (Gibco, 10378016), 2.5 µg/mL N-tosyl-L-phenylalanine chloromethyl ketone-treated (TPCK-treated) (Sigma-Aldrich, 4370285), and DMEM (Gibco, 10312021). The chemical modification in cellulo was performed 24 h after infection.

### Virus purification on a sucrose cushion

Virus stocks were purified via centrifugation on a sucrose cushion. 140 µL of virus stock (Focus Forming Units (FFU) = 3 × 10^6^/mL) was gently pipetted over 1400 µL of sucrose cushion (20% sucrose in TNE buffer containing 50 mM Tris–HCl, 100 mM NaCl, 0.1 mM EDTA). Next, the mixture was centrifuged for 6 h at 14,000 g. The supernatants were discarded and the viral pellets were dissolved in 180 µL of resuspension buffer (0.01 M Tris–HCl pH 7.4, 0.1 M NaCl, 0.0001 M EDTA) [18].

### Chemical probing and library preparation

**Fig. 1:**
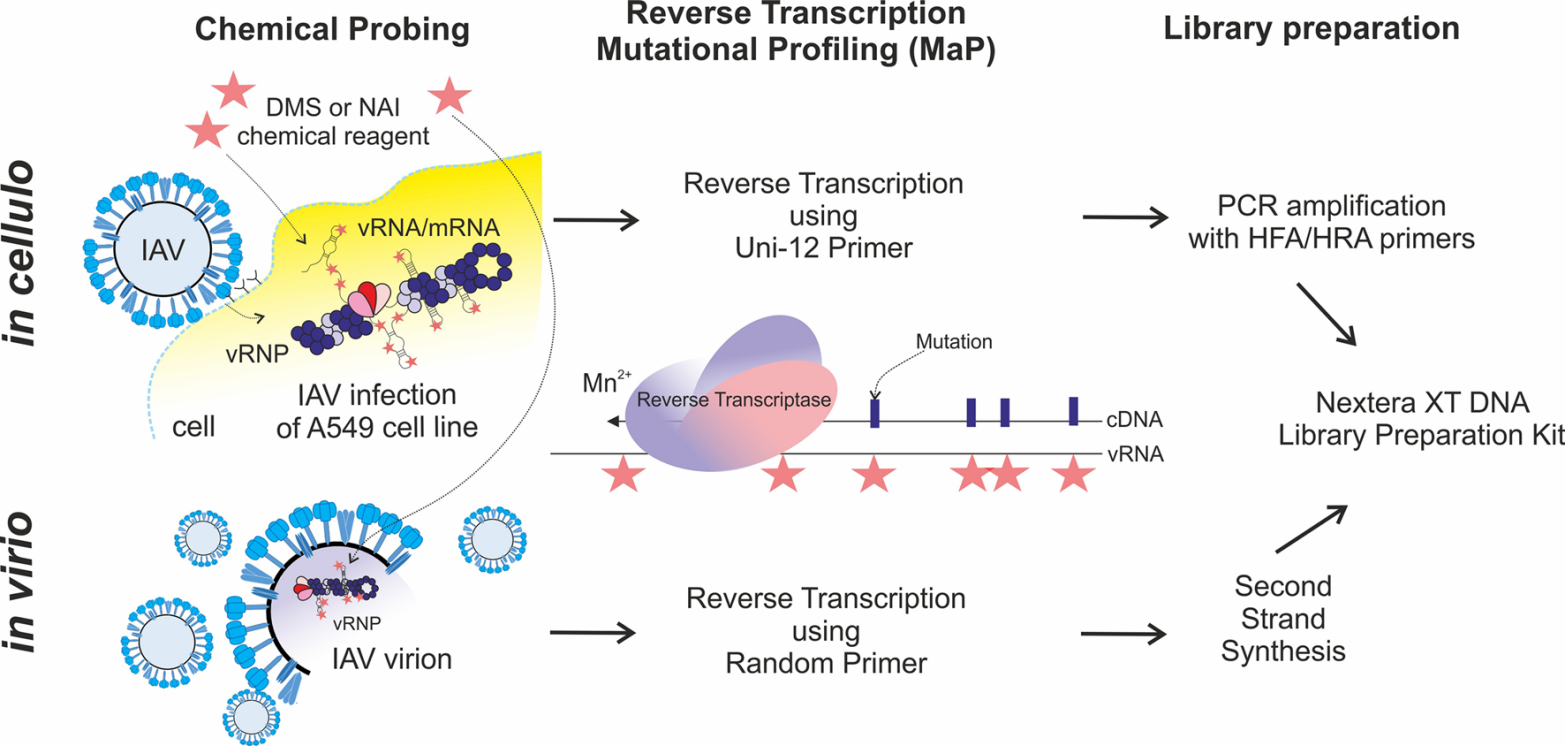
Experimental workflow for in virio and in cellulo experiments

Chemical mapping was carried out directly on virions concentrated via sucrose-cushion centrifugation (in virio) and on IAV-infected A549 cells (in cellulo). RNA originating from in virio and in cellulo experiments was treated according to published SHAPE-MaP protocols, although with different workflows (Fig. 1) [40]. For the in virio experiments, we used a workflow with random-priming RT reactions, followed by second-strand synthesis. For RNA from the IAV-infected cells, we used an IAV-universal “Uni-12” primer complementary to the 3’-end of each vRNA [41]. Next, the cDNA product was amplified with universal 5’- and 3’-primers (HFA, HRA) fused with transposase adapters on each end [42]. Detailed protocols for library preparation are described below. Each experiment was performed in three independent biological replicates.

### RNA structure chemical probing of IAV-infected A549 cells

The RNA structure in cellulo chemical probing protocol was developed based on published protocols [18, 27, 29]. For the chemical probing, two chemical reagents were used: DMS (Sigma-Aldrich, 274380) and NAI. The NAI reagent was synthesized according to Spitale et al. [43]. Before the modification, the cells were washed twice with 1 × PBS and then treated with trypsin–EDTA solution (Sigma-Aldrich, T4049) at 37 °C for 5 min, washed from a plate with a complete growth medium, and transferred to 1.5 mL tubes. Next, the cells were purified via centrifugation (5 min, 3000*g*) and washed once with 1 × PBS buffer. Purified cellular pellets were suspended in 180 µL of 1 × PBS buffer. For DMS probing, 20 µL of 3% DMS diluted in absolute ethanol was added (0.3% final) and the reaction was incubated at 23 °C for 5 min. The control reaction was treated with the same volume of ethanol. The reaction was quenched with quench buffer (50 mM Tris–HCl pH 7.5, 100 mM NaCl, 3 mM MgCl_2_, 40 mM β-mercaptoethanol) and cells were centrifuged for 3 min at 3000*g*. The cell pellets were washed twice with quench buffer before suspending them in 225 µL of 1 x PBS. The total RNA was isolated with Trizol LS (Invitrogen, 10296010) according to the manufacturer’s protocol. For NAI probing, 1 M stock of NAI dissolved in anhydrous dimethyl sulfoxide (DMSO) (Sigma-Aldrich, 276855) was prepared just before the reaction. To 180 µL of PBS, 20 µL of 1 M NAI (100 mM final) for the modification reaction (or 20 µL of DMSO for the control reaction) was added to the cell suspension and incubated for 8 min at 37 °C. The reaction was quenched by incubation for 5 min at 37 °C with 30 µL of 1 M dithiothreitol (DTT) and centrifuged (3 min, 3000*g*). The cell pellets were then washed once with 1 x PBS supplemented with 160 mM DTT and centrifuged. The cell pellets were suspended in 225 µL of 1 x PBS before total RNA isolation with Trizol LS according to the manufacturer’s protocol.

### Chemical probing of IAV vRNA in virio

NAI and DMS chemical reagents were used for the chemical probing in virio. For the NAI probing, 20 µL of 1 M stock of NAI (100 mM final in reaction sample) or anhydrous DMSO (control sample) was added to the viral suspension in the resuspension buffer (0.01 M Tris-HCl, pH 7.4, 0.1 M NaCl, 0.0001 M EDTA). After 10 min incubation at 37 °C, the reaction was quenched by adding 20 µL of 1 M DTT, and the reaction was incubated for 5 min at 37 °C before the viral RNA was isolated according to the manufacturer’s protocol with Trizol LS. The same volume of 1 M DTT was added to the control sample as well. For DMS probing, 20 µL of 4% DMS diluted in absolute ethanol (reaction sample, 0.4% final) or 20 µL of ethanol (control sample) was added to the viral suspension, and the reaction was incubated for 5 min at 37 °C. The reaction was quenched with 20 µL 1 M DTT and incubated for 5 min before RNA isolation with Trizol LS.

### SHAPE-MaP and DMS-Map—in cellulo library preparation

Total RNA isolated from cells (chemically mapped samples and controls, separately) was treated with DNase I, followed by on-column purification with a QIAGEN RNeasy Mini Kit (QIAGEN, 74104). The RNA integrity number (RIN) of the total RNA sample was checked via RNA IQ Assay on Qubit Fluorometer (Invitrogen, Q33221), and only RNA with a RIN > 8.5 was used for the final experiment. Reverse transcription using SuperScript II (Invitrogen, 18064014) was prepared according to the SHAPE-MaP protocol [40]. 3 µg of total RNA from cells was reverse transcribed using 1 µL of 10 µM Uni-12 primer [41]. The product was purified on an Illustra Microspin G-25 column (GE Healthcare, GE27-5325-01). Next, the cDNA was used for a PCR using Phusion polymerase (Thermo Fisher, F531L) with IAV universal HFA and HRA primers from the Illumina protocol [42]. The primers were ordered from Merck. For the PCR of the final 50 µL volume, 10 µL of cDNA was combined with 12.5 µL of 10 mM MgCl_2_, 1 µL of 10 µM of HFA and HRA primers (0.2 µM final), and 1 × Phusion Master Mix with HF Buffer. The reaction was initially denatured for 30 s at 98 °C, cycled 30 times (98 °C for 30 s, 58 °C for 45 s, 72 °C for 90 s), and extended for 10 min at 72 °C. The reaction was cleaned up using 0.5 × Ampure XP Beads (Beckman Coulter, A63882) before library preparation with Nextera XT DNA Library Preparation Kit (Illumina, FC-131-1096). The final library was size selected using 0.6 × and 0.15 × Ampure XP beads (double-size selection).

For the additional control sample of chemical mapping in cellulo, 1 µg of IAV-infected total RNA was used for RT reaction with 1 µl of 60 µM random primer mix (NEB, S1330S). The product was purified on the G-25 column, followed by second-strand synthesis using NEBNext Ultra II Non-Directional RNA Second Strand Synthesis Module (NEB, E6111L) according to manufacturer protocol but with an extended time of incubation to 2.5 h at 16 °C. Next, the DNA product was purified with a PureLink PCR Micro Kit (Invitrogen, K310050), and 1 ng of DNA was intended for library preparation with Nextera XT DNA Library Preparation Kit (Illumina, FC-131-1096). The final library was purified with 0.6 × Ampure XP Beads.

### PCR with segment-specific primers

Segment-specific primers were used to control the presence of all vRNA fragments in PCR products obtainted with HFA/HRA primers (Supplementary E1 Table 1). The PCR was prepared in the same way as described above.

### SHAPE-MaP and DMS-MaP—in virio library preparation

For the library preparation from in virio samples, 50–100 ng of viral RNA was reverse transcribed using 1 µl of 60 µM random primer mix (NEB), followed by purification on the G-25 Column. The whole cDNA was used for second-strand synthesis (NEB) and the incubation time at 16 °C was extended to 2.5 h. The DNA product was purified on a PureLink PCR Micro Kit, and 1 ng of DNA was used for library preparation with Nextera XT DNA Library Prep. Kit. The final library was purified using 0.6 × Ampure XP Beads.

### Sequencing and data analysis

The libraries were paired-end sequenced (2 × 150 bp) on Mid Output Flow Cell using the NextSeq550 instrument. Bioinformatic analysis was performed with ShapeMapper software version 2.1.5 [44] with the min-depth option set to 1000. Modified and untreated paired reads were analyzed simultaneously for each segment. We obtained reactivity data for all eight full-length segments of vRNA in virio (Supplementary E1, Table 2). We obtained reactivity data encompassing the 5ʹ- and 3ʹ-ends for all eight vRNAs while missing the central regions for segments 1, 2, 3, 4, and 6. The reactivity data for segments 5, 7, and 8 in cellulo are available in Supplementary E1 Table 3.

### Analysis of the control 18S rRNA

The 3D structure of the human ribosome (PDB ID: 4v6x) was analyzed and presented using Chimera software [45]. The secondary structure of 18S rRNA was retrieved from the RNAcentral database, ID: URS00005A14E2_9606 (https://rnacentral.org/rna/URS00005A14E2/9606). The reactivity data for the control 18S rRNA are available in Supplementary E1 Table 4.

### Shannon Entropy calculation

Shannon Entropies were calculated from partition function data for local and global structure predictions. To estimate the extent to which one structure dominates in each region of the sequence, we calculated the Shannon Entropy per nucleotide (*S*_*i*_) as:

$${S}_{i}=\sum_{j=1}^{N}{P}_{i,j}{\text{log}}_{10}\left({P}_{i,j}\right),$$

where *i* and *j* are nucleotide indices and *P*_*i,j*_ is the probability of the i-j base-pair. Only valid i-j pairs are included. The pair probabilities are estimated using the partition function in RNAstructure, version 6.4 [46]. Shannon entropies are lower for better defined structures [47]. Median Shannon Entropies were calculated for the center nucleotide in sliding 50 nt windows (OriginPro2021).

### Base-pairs conservation analysis

Data were downloaded from the Influenza Research Database (fludb.org) on August 11th, 2021 using the following parameters: nucleotide data, virus type A, any host, any country/region, segments 1 through 8, any subtype, full-length only, and collapse identical sequences. This resulted in eight segment databases of (in increasing segment order) 46416, 46287, 46821, 70927, 41210, 57097, 36731, and 36411 sequences for a total of 381360 sequences. The retrieved sequences were reverse-complemented to a negative sense and were then aligned to A/California/04/2009 vRNA1-8 sequences. For the alignment, we used the Multiple Alignment using Fast Fourier Transform (MAFFT) web server and the Fast Fourier Transform progressive method (FFT-NS-2) with low memory mode. A custom Python script was then used to count the base pairs observed in each sequence based on the experimental secondary structures. All canonical pairings (including GU wobble pairs) were considered to be conserved. All custom Python scripts can be found at https://github.com/moss-lab/scripts. Base-pairing conservation analyses can be found in Supplementary E2-E5. The motifs with the highest base-pairing conservation (≥ 95%) in virio and in cellulo are gathered in Tables 1 and 2 of Supplementary E6.

### Analysis of codon conservation

Plus sense sequence databases were aligned using MAFFT, as previously described. After identifying regions of interest, a custom Python script was used to count codon frequencies in the major reading frame of each vRNA segment. Any region spanning a start codon was aligned with the reading frame, regardless of actual translational capacity. Two vRNA7 regions (35–60, 144–166) were aligned with the spliced matrix protein 2 (M2) reading frame, as they were beyond the stop codon for the larger, un-spliced matrix protein 1 (M1). Motif alignments to codon frequency data can be found in Supplementary E7.

### Statistical analysis

Before calculating the average reactivity values from both SHAPE and DMS probing, we examined the Pearson Correlation Coefficient (PCC) between all biological replicates. The PCC for each pair of reactivity datasets was calculated with OriginPro2021 (OriginLab). The correlation coefficients are presented in Supplementary F1 Fig. S1. PCC between reactivity datasets in virio and in cellulo was calculated using the MovCoef function over a sliding 50 nucleotide window (Supplementary F1 Fig. S2). The Pearson coefficient (*r*) ranges from − 1 to 1, where *r* ≥ 0.9 means very high positive correlation, 0.7 ≤ *r* < 0.9 means high positive correlation, 0.5 ≤ *r* < 0.7 means moderate positive correlation, and < 0.5 means low positive correlation. All additional statistical analyses were performed with the OriginPro2021 program. We found our data to be highly reproducible between independent biological experiments. Pearson coefficients for reactivities of individual vRNAs ranged from *r* = 0.77 to 0.99 indicating a high correlation between replicates. The lowest correlation was observed in the case of vRNA segments from the in cellulo experiment for which we obtained an incomplete subset of data.

### RNA secondary structure prediction

The average reactivities for all the nucleotides were calculated from three independent replicates from the DMS and NAI experiments. For the structure prediction, RNAstructure ver. 6.3 was used with default parameters (intercept -0.6 kcal/mol, slope 1.8 kcal/mol) [46]. For the calculation of Minimum Free Energy (MFE) structure, SHAPE (NAI) reactivities were implemented as pseudo-energy constraints, while DMS (reactivities ≥ 0.85) were implemented as chemical modification constraints [48, 49]. Additionally, partition function calculations were conducted using RNAstructure with the implementation of the experimental mapping data of a particular vRNA, as described below. The partition function calculation was used for the generation of Maximum Expected Accuracy (MEA) structures via the RNAstructure program, as well as for the calculation of the base-pairs probability of predicted vRNA secondary structures [50].

**Fig. 2:**
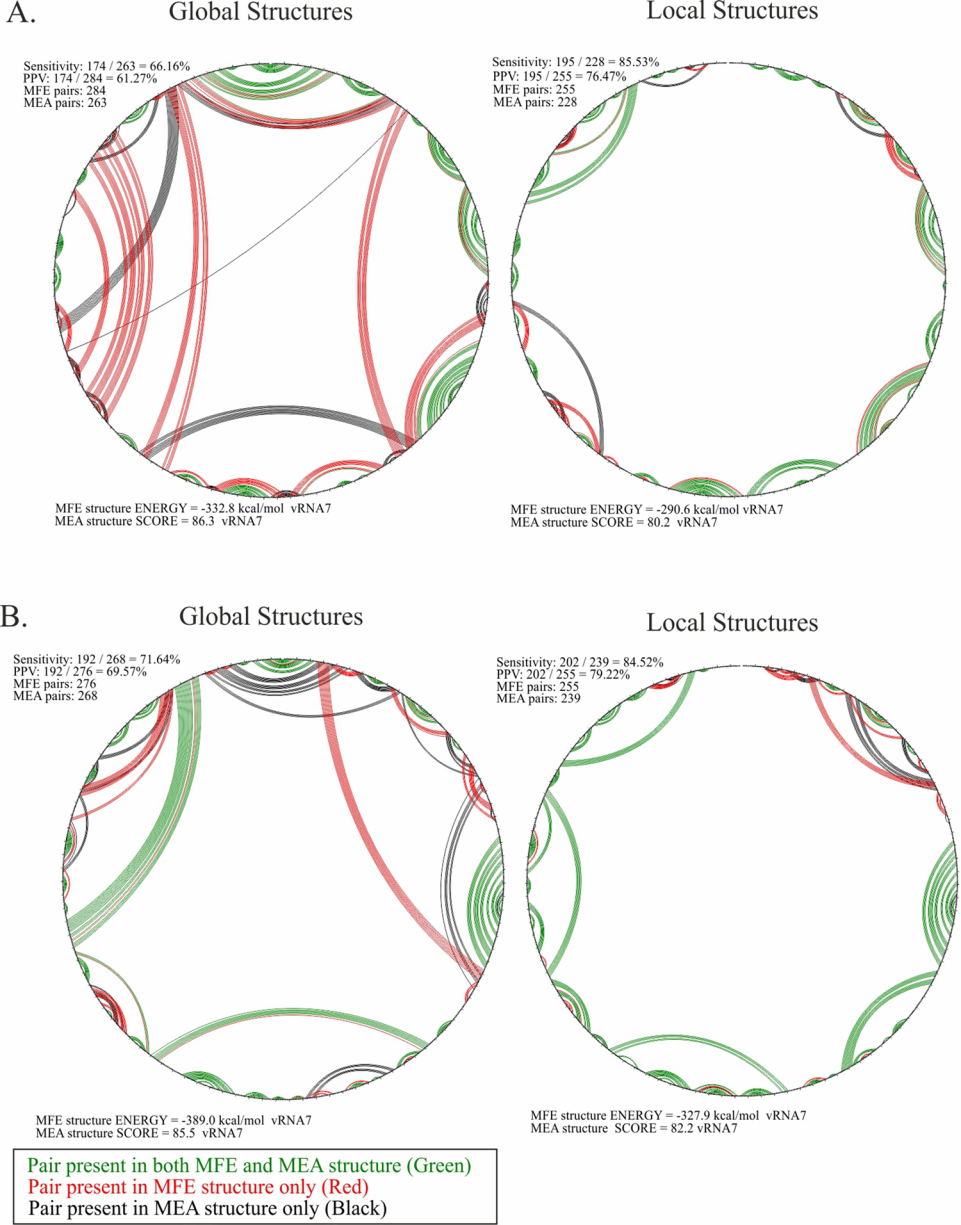
Comparison between MFE and MEA structures of vRNA7 predicted in global and local context. Comparison via CircleCompare tool (RNAstructure). The comparison shows similarities (green) and differences (red) in the base-pairing between the MFE and MEA structure predictions. In MEA, the score is = 2*sum(Pij) + sum(Pk) where Pij is the probability for the base pair i-j for all pairs and Pk is the probability that k is unpaired for all unpaired nucleotides. **A** Comparison between MFE and MEA vRNA7 structure predictions from in virio experiment. **B** Comparison between MFE and MEA  vRNA7 structure predictions from in cellulo experiment

We used two algorithms for the secondary structure prediction: MFE and MEA. Additionally, both folding algorithms were calculated using two approaches: the first without constraining the base-pairing distance, and the second limited to a distance equal to 150 nt. Next, the in virio and in cellulo global and local MFE/MEA structures were compared using the CircleCompare tool (RNAstructure) (Fig. 2; Supplementary F1 Figs. S3–S6 in virio and Fig. S7 in cellulo). The CircleCompare tool enables the comparison of similarities between structures. For such an estimation, two measures are used: sensitivity corresponding to the percentage of the base-pairs common in both compared structures, and the positive predictive value (PPV), which measures the percentage of base-pairs predicted in both structures relative to the total number of base-pairs in the predicted structure [50].

## Results

### Mutational profiling and nucleotide reactivities of the IAV genomic RNA

We compared in virio and in cellulo probing data for vRNA segments from which we obtained a complete subset of the nucleotide reactivities (segments 5, 7, and 8). A comparison of the average reactivity profiles between in cellulo and in virio showed that average nucleotide reactivities in those environments were similar in both NAI and DMS probing experiments (Supplementary F1 Fig. S8 and S9). The PCC calculated for reactivities of segments vRNA5, 7, and 8 showed a high positive correlation between in virio and in cellulo experiments (Supplementary F1 Fig. S2). Calculation of the reactivity difference ΔNAI and ΔDMS at the single-nucleotide level showed that the vRNA nucleotides were mostly more reactive in cellulo (Supplementary F1 Fig. S10).

### Chemical mapping results of human 18S rRNA

We used human 18S ribosomal RNA (rRNA) as a control for in cellulo chemical mapping experiments using NAI and DMS chemical reagents (Supplementary E1 Table 4). 18S rRNA is a component of the small ribosomal subunit, and it has a known structure [51]. We correlated the modification sites resulting from our experiments with the published secondary structure and cryo-EM tertiary structure of 18S rRNA within the human ribosome (PDB ID: 4v6x). Modification sites for 18S rRNA were found within single-stranded and solvent-accessible regions (Supplementary F1 Fig. 11). Further, sites that appear single-stranded based on the secondary structure but are hidden inside the ribosomal core were not modified.

### Secondary structure prediction of IAV vRNA in virio and in cellulo

We found that more single-stranded regions were predicted in vRNA MEA structures when compared to MFE (Fig. 2; Supplementary F1 Figs. S3–S7; vRNA MFE structures were implemented as accepted and MEA as predicted structures). Interestingly, the PPV and sensitivity values for the global structures increased with the length of the vRNA segments (Table 1). In contrast, a comparison of the local structures showed that the PPV and sensitivity values were higher for shorter vRNA segments and that the values decreased with length (Table 1). On each vRNA segment, both in virio and in cellulo, we observed many locally folded motifs predicted in both the MFE and MEA structures. Global vRNA structures indicated many long-range interactions occurring within each vRNA, regardless of the algorithm used for the secondary structure prediction (Table 2). The secondary structures (MEA, MFE, local, and global) of particular vRNAs are shown in Supplementary F2.

**Table 1:** The PPV and sensitivity values between global and local MFE/MEA vRNA structures in virio and in cellulo calculated using CircleCompare tool (RNAstructure).

Segment	Global		Local	
	PPV	Sensitivity	PPV	Sensitivity
In virio				
vRNA1	84.04	59.34	52.91	41.93
vRNA2	90.98	59.97	81.77	62.06
vRNA3	81.16	68.30	81.57	69.02
vRNA4	75.00	52.90	80.37	65.83
vRNA5	83.68	70.88	89.31	70.82
vRNA6	76.47	49.09	89.50	78.60
vRNA7	66.16	61.27	85.53	76.47
vRNA8	76.43	57.97	90.91	75.68
In cellulo				
vRNA5	71.64	60.61	80.63	69.02
vRNA7	71.64	69.51	94.52	79.22
vRNA8	41.24	40.20	90.91	78.65

**Table 2:** List of long-range interactions predicted in both MFE and MEA global structures of particular vRNA segment in virio and in cellulo

In virio	
Segment	Base-pairing [nt] 5ʹ–3ʹ/3ʹ–5ʹ
vRNA1	89–123/2279–2251
	247–254/2142–2135
	370–374/1660–1664
	436–450/1653–1638
	617–638/1119–1098
	688–692/1082–1078
	839–846/1028–1021
vRNA2	114–127/2310–2299
	129–142/1913–1902
	830–837/1697–1690
	930–941/1591–1578
	953–958/1514–1509
	960–968/1417–1409
	971–990/1387–1367
	1936–1942/2282–2276
	2040–2047/2269–2261
vRNA3	23–50/1947–1920
	52–52/1807–1805
	96–102/1784–1778
	494–500/1760–1753
	609–622/1745–1733
vRNA4	235–241/1543–1537
	652–675/1158–1134
	685–697/1086–1076
vRNA5	29–44/524–511
	117–137/434–419
	140–148/398–382
	526–528/1457–1455
	598–602/949–945
vRNA6	180–183/689–686
	290–295/629–624
vRNA8	92–95/619–621
	189–199/606–597
	210–222/416–405
	645–652/863–856

In cellulo	
Segment	Base-pairing [nt] 5'-3'/3'-5'
vRNA5	29–34/1458–1453
	40–44/1405–1401
	939–949/1357–1348
	951–958/1314–1307
	1000–1008/1267–1260
	1025–1029/1216–1212
vRNA7	396–401/620–614
	720–741/964–945

### Comparison of vRNA secondary structures in virio and in cellulo

**Fig. 3:**
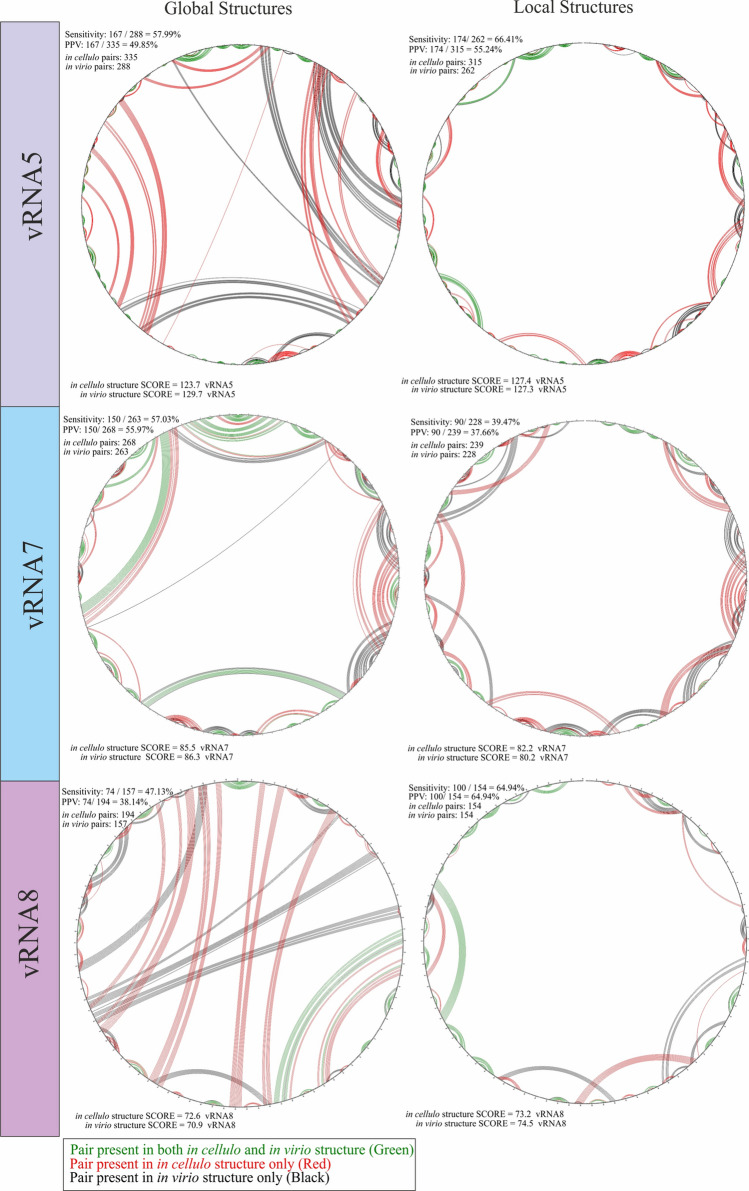
Comparison via CircleCompare tool (RNAstructure) between in virio and in cellulo MEA structures of vRNA segment 5, 7, and 8 predicted in global and local context. We introduced in cellulo MEA structure as the predicted structure and in virio MEA as the accepted structure. The comparison shows similarities (green) and differences (red) in the base-pairing between the structure predictions in different environments. In MEA, the score is = 2*sum(Pij) + sum(Pk) where Pij is the probability for the base pair i-j for all pairs and Pk is the probability that k is unpaired for all unpaired nucleotides

The structures of vRNA5, 7, and 8 in virio and in cellulo showed varying levels of similarity depending on the particular segment and global/local context. The CircleCompare tool (RNAstructure) was used to estimate the similarity and variability of vRNA structures in virio and in cellulo for segments 5, 7, and 8 (Fig. 3). We observed a higher number of base pairs in the case of nearly all in cellulo vRNA structures, except for vRNA8 local structure which has the same number of base pairs in both in cellulo and in virio (Fig. 3). The highest PPV and sensitivity values were observed in the case of the comparison of the global structures of vRNA7 (sensitivity: 57.03%, PPV: 55.97%), followed by global vRNA5 structures (sensitivity: 57.99%, PPV: 49.85%). The highest difference between global MFE and MEA structures was in the case of the vRNA8, where sensitivity was 47.13% and PPV was 38.14%. Interestingly, a different trend was observed in the comparison of the local structures. The highest similarity was found in local vRNA8 structures (sensitivity: 64.94%, PPV: 64.94%), followed by local vRNA5 structures (sensitivity: 66.41%, PPV: 55.24%). The highest difference between the predicted MEA and MFE local structures was noticed for vRNA7, where the sensitivity and PPV values were 39.46% and 37.66%, respectively. We observed numerous structural motifs that are common in in virio and in cellulo environments (Table 3). Some motifs extend this commonality further between predicted global and local structures; for example, vRNA5 motifs in regions 87–115 nt, 349–366 nt, 746–776 nt, 809–823 nt, 1075–1110 nt, 1142–1160 nt, 1193–1210 nt, 1460–1522 nt, and 1527–1555 nt were predicted in local and global MEA structures in virio and in cellulo. Other motifs exist exclusively in global or local predictions; vRNA5 motifs in regions 46–63 nt, 614–627 nt, 1036–1051 nt, 1055–1072 nt, and 1371–1388 nt were predicted only in global MEA structures, but not in the local context. Motifs exclusive to in virio and in cellulo vRNA5 local structures were predicted in regions 125–165 nt, 511–527 nt, 952–1064 nt, and 1262–1275 nt.

**Table 3:** Structural motifs (≥ 5 base pairs) of segments vRNA5, vRNA7, and vRNA8 common for in virio and in cellulo environments in the local and global context (MEA structures)

	vRNA5		vRNA7		vRNA8	
	Global	Local	Global	Local	Global	Local
Sensitivity	57.99%	66.41%	57.03%	39.47%	47.13%	64.94%
PPV	49.85%	55.24%	55.97%	37.66%	38.14%	64.94%
	vRNA5 motifs (base-pairing [nt])		vRNA7 motifs (base-pairing [nt])		vRNA8 motifs (base-pairing [nt])	
	1–16/1551–1565	**87–98/115–104**	1–16/1113–1027	35–41/54–60	1–16/890–876	**65–79/76–80**
	46–51/57–63	125–142/146–165	28–49/973–984	64–69/73–78	**65–79/76–80**	262–270/279–287
	**87–98/115–104**	**349–355/360–366**	54–69/73–88	96–110/115–128	210–222/404–407	**312–317/322–327**
	**349–355/360–366**	511–516/522–527	136–141/168–171	**452–456/461–465**	262–270/279–287	**461–465/473–477**
	614–618/624–627	**746–757/763–776**	257–265/286–292	521–531/537–548	**312–317/322–327**	531–541/546–557
	**746–757/763–776**	**809–815/818–823**	405–415/419–426	**692–698/707–712**	**461–465/473–477**	565–578/581–504
	**809–815/818–823**	952–963/1053–1064	**452–456/461–465**	**788–796/801–809**	747–755/759–768	596–604/719–728
	**974–979/982–988**	974–979/982–988	525–531/537–544	**845–851/856–862**	**794–800/806–812**	645–649/662–666
	1036–1040/1047–1051	**1075–1092/1198–1110**	625–629/638–642	**904–914/921–931**		768–775/782–789
	1055–1061/1066–1072	**1142–1146/1156–1160**	654–658/680–685			**794–800/806–812**
	**1075–1092/1198–1110**	**1193–1199/1204–1210**	**692–698/707–712**			844–857/861–877
	**1142–1146/1156–1160**	1262–1266/1271–1275	**788–796/801–809**			
	**1193–1199/1204–1210**	1460–1479/1495–1522*	**845–851/856–862**			
	1371–1375/1384–1388	**1527–1537/1540–1555**	**904–914/921–931**			
	1460–1479/1495–1522*					
	**1527–1537/1540–1555**					

*Slight difference in the base-pairing

Motifs predicted in both global and local vRNA structures are bolded. The calculated PPV and Sensitivity values between MEA vRNA structures are included

Next, we checked for the presence of long-range interactions common between in virio and in cellulo global MEA vRNA structures. In the case of vRNA5, we did not observe such interactions. For vRNA7, we observed common long-range interactions in regions: 26–49/974–993 nt (without 44/979 pair), 396–401/620–614 nt, and 732–741/954–945 nt. In the case of vRNA8, one long-range interaction in region 210–222/405–416 nt (without 407/220 pair) was observed in both environments (Fig. 3).

### Base-pairing conservation analysis

**Fig. 4:**
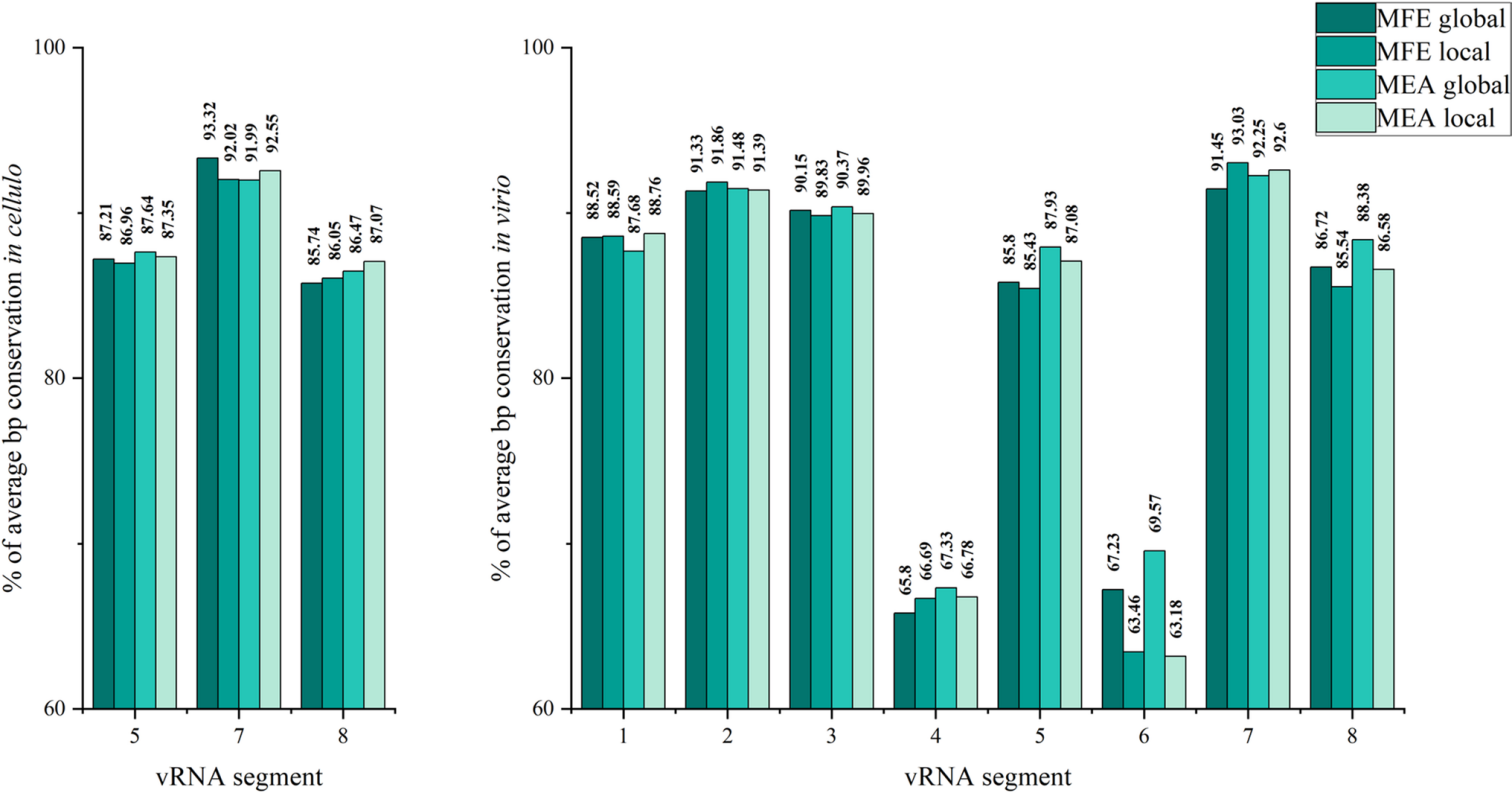
Average base-pairing (bp) conservation (%) for vRNA segments 5, 7 and 8 in cellulo as well as all vRNA segments in virio. The calculation was performed independently for every generated structure—MFE and MEA in global and local folding prediction

We examined the base-pairing conservation of MFE and MEA structures in both global (Supplementary E2, E3) and local (Supplementary E4, E5) contexts. Average vRNA structure conservation in cellulo and in virio was very similar between nearly all generated structures (Fig. 4). In detail, the lowest difference (%) between the highest and the lowest base-pairing conservation percentages were observed for vRNA2 in virio (0.53%) and vRNA5 in cellulo (0.68%), while the highest differences (%) were observed for vRNA6 in virio (6.39%) and vRNA8 in cellulo (1.52%). The highest average structure conservation was observed for vRNA7 in both in virio (93.03% for MFE local structure) and in cellulo (93.32% for MFE global structure). Apart from vRNA7, the highest conservation in virio was observed in segments 1–3, which code for the viral polymerase complex (vRNA1: 91.86% for MFE local structure, vRNA2: 92.86% for MFE local structure, vRNA3: 90.37% for MEA global structure). The lowest average conservation in virio was calculated for vRNA6 (63.18% for MEA local structure) and vRNA4 (65.8% for MFE global structure), which code the most variable of IAV proteins, the subtype-defining viral surface glycoproteins: hemagglutinin and neuraminidase.

Thereafter, we performed a detailed analysis to find particular RNA motifs in virio and in cellulo with the highest base-pairing conservation (≥95%) (Supplementary E6). The analysis confirmed the conservation of long-range intramolecular interactions, including the *panhandle* motif formed between the 5ʹ- and 3ʹ-ends of all vRNA segments. Notably, in the case of vRNA4 and 6, we did not find any additional highly conserved (≥ 95%) structural motifs in virio (Supplementary E6). Other vRNA segments had multiple conserved motifs predicted within all generated structures, both MEA and MFE, in the local and global context (Supplementary E6). Interestingly, some motifs of high conservation were found in local or global, MFE or MEA structures exclusively. For example, in the case of segment 7 vRNA in virio, a hairpin in region 34–41/61–54 nt (99.86% of conservation) was predicted in both local structures, but was not found in global contexts. In contrast, a hairpin motif in region 115–119/128–124 nt was predicted in global MFE and MEA structures only. Another example is a hairpin formed in region 788–796/809–801 nt (99.77% of conservation) which was predicted in all generated structures. Other highly conserved short hairpins in regions 96–99/128–124 nt (99.89%) and 323–336/408–393 nt (96.46%) were predicted in the local MEA structure only. In the case of in cellulo vRNA structure prediction, we also found some differences in conserved motifs between versions of the predicted structures of particular vRNA (Supplementary E6). For example, in segment 7 vRNA, one hairpin in region 788–796/809–801 nt (99.33%) was predicted in all generated structures, while a hairpin in region 64–69/78–73 nt (97.81%) was found in nearly all predictions, except for local MFE structure. Accordingly, a highly conserved (99.96%) hairpin in region 935–942/954–946 nt was predicted in local structures only. Apart from locally folded structural motifs, we noted highly conserved long-range interactions predicted in global structures, such as the pairing in region 40–44/1405–1401 nt (96.67% of conservation) predicted in MFE and MEA global vRNA5 structures in cellulo.

### The most stable locally folded vRNA motifs of potential functionality

**Fig. 5:**
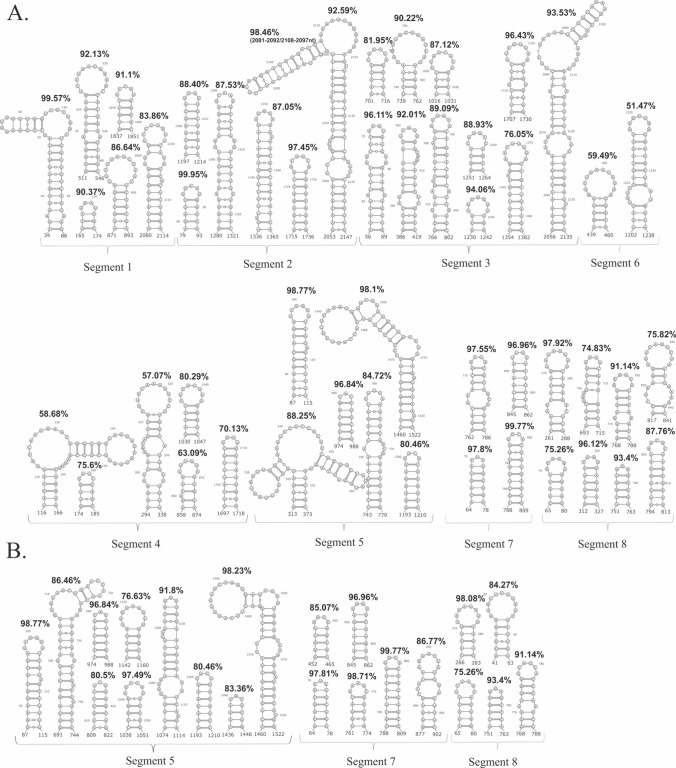
Structural motifs predicted in vRNA secondary structures in low Shannon entropy-low SHAPE-low DMS regions. The conservation percentage across Influenza A strains of each motif is indicated above. **A** The well-defined motifs of each vRNA in virio. **B** The well-defined vRNA5, vRNA7 and vRNA8 motifs in cellulo

**Fig. 6:**
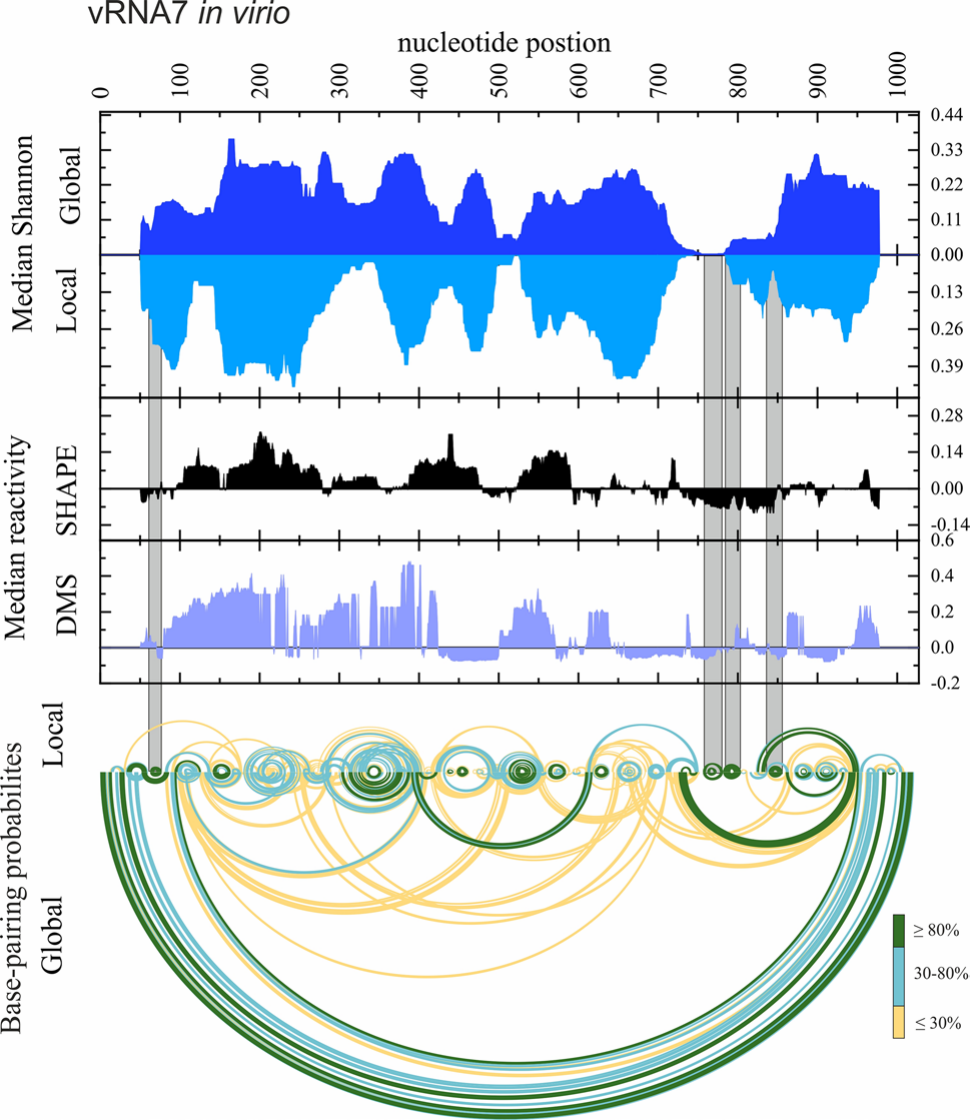
The secondary structure of vRNA7 in virio. Median Shannon Entropies of global and local structures were calculated in centered, sliding 50 nt window. Median SHAPE (NAI) and DMS reactivities were calculated in 50 nt window and plotted with respect to global median. Arc plots showing the base-pairing probabilities of predicted local (upper) and global (lower) structures were calculated using partition function (RNAstructure). Grey shadings indicate the low Shannon entropy-SHAPE-DMS regions of the most probable, well-defined structural motifs predicted in both—global and local vRNA7 secondary structures. Regions of 50 nt from both ends of vRNA7 (graphs: Median Shannon, and Median reactivity) were excluded from visualization

**Fig. 7:**
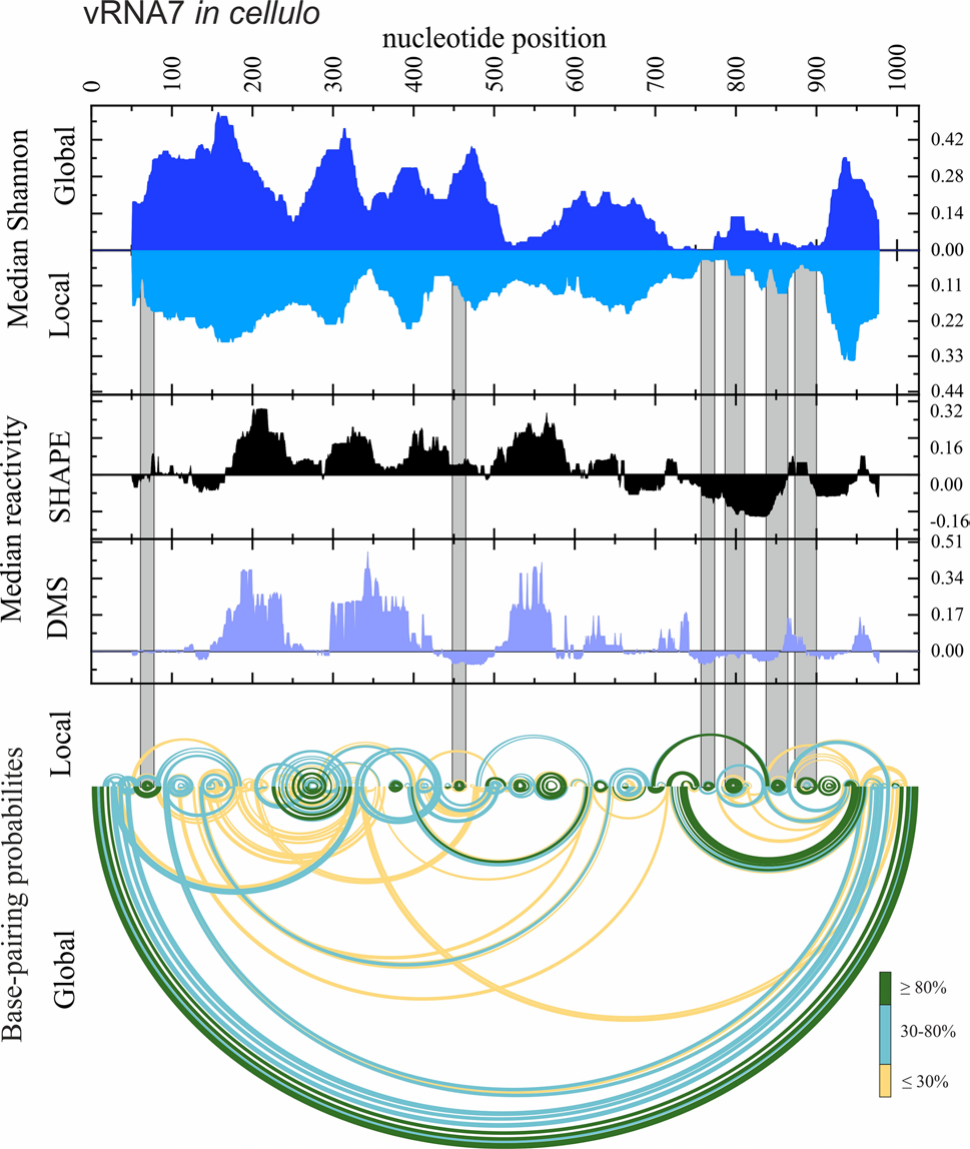
The secondary structure of vRNA7 in cellulo. Median Shannon Entropies of global and local structures were calculated in centered, sliding 50 nt window. Median SHAPE (NAI) and DMS reactivities were calculated in 50 nt window and plotted with respect to global median. Arc plots showing the base-pairing probabilities of predicted local (upper) and global (lower) structures were calculated using partition function (RNAstructure). Grey shadings indicate the low Shannon entropy-SHAPE-DMS regions of the most probable, well-defined structural motifs predicted in both—global and local vRNA7 secondary structures. Regions of 50 nt from both ends of vRNA7 (graphs: Median Shannon and Median reactivity) were excluded from visualization

For the identification of the most stable, well-determined local RNA structural motifs, we identified low-Shannon entropy, low-DMS, and low-SHAPE regions (Figs. 5, 6, 7; Supplementary F1 Figs. S12–18 in virio*,* S19–20 in cellulo). In such regions, it is most probable that single well-defined structural motif is formed [26, 27]. As mentioned before, we did not obtain sufficient sequencing coverage to allow us to calculate nucleotide reactivities from in cellulo structure probing for the internal fragments of vRNA segments 1, 2, 3, 4, and 6. Therefore, we decided not to analyze the partial data for these vRNA segments. For the rest of the RNA segments, we identified multiple well-determined structural motifs in virio (Fig. 5A) and in cellulo (Fig. 5B). Notably, many of these motifs have high structural-sequence conservation across IAV strains, indicating their possible functionality. Comparison of motifs predicted in vRNA structures in both environments led to the discovery of motifs common to in virio and in cellulo conditions, like hairpin motifs in vRNA5 (87–115 nt, 974–988 nt, 1193–1210 nt, and 1460–1522 nt), vRNA7 (64–78 nt, 788–809 nt, 845–862 nt), and vRNA8 (65–80 nt, 266–283 nt, 751–763 nt, 768–788 nt) (Fig. 5). All of these motifs were predicted in regions indicated as responsible for the packaging processes of nascent vRNAs [19].

### vRNA motifs common to different IAV strains

We previously proposed in vitro secondary structures of segments 5 and 8 of strain A/California/04/2009 and segments 5, 7, and 8 of strain A/Vietnam/1204/2003 [24, 33–37]. So far, these segments have been the most studied. The secondary structures of segments 1, 2, 3, 4, and 6 have only been proposed in one in virio study by Dadonaite et al. [18].

We selected the in virio and in cellulo vRNA structural motifs predicted here that were proposed in our in vitro predictions, as well as in virio vRNA secondary structures of strain A/WSN/1933 proposed by Dadonaite et al. [18]. We indicated the base-pairing conservation of each motif. Additionally, we calculated the sequence conservation of these motifs in all IAV strains.

We found that several vRNA secondary structure motifs proposed in this study were also predicted in virio for vRNA1, 2, 3, 4, and 6 of A/WSN/1933 (Table 4) [18]. Only three motifs common to both strains have very high base-pairs and sequence conservation: 50–63 nt (vRNA1), 79–93 nt, and 2301–2311 nt (vRNA2) (Table 4). The other motifs have lower conservation (both sequential and structural) among IAVs, which may indicate that they are more conserved for the A/H1N1 subtype.

**Table 4:** Common structural motifs of vRNA segments 1, 2, 3, 4 and 6 for A/California/04/2009 and A/WSN/33 strains in virio [18]

Segment	Sequence identity [%]	Motif region [nt]	Base-pairs conservation [%]	Nucleotide sequence conservation [%]
vRNA1	86	13–33	87.91	93.74
		50–63	99.89	98.64
		1122–1135	90.69	90.31
		1837–1851	97.10	93.45
vRNA2	83	79–93	99.95	99.53
		436–456	87.54	88.85
		621–638	96.04	88.92
		1333–1367	90.60	88.89
		2301–2311	99.84	99.30
vRNA3	85	457–465	97.20	88.42
		1279–1292	90.90	94.35
vRNA4	80	133–156	55.26	67.73
		637–657	67.19	82.75
vRNA6	81	693–703	91.11	76.34
		832–852	67.21	72.94

The sequence identity of each segment (full-length) was calculated via ClustalW webtool (www.genome.jp). Motifs with > 80% base-pairing probability (from RNAstructure) were selected. Base-pairs conservation as well as nucleotide sequence conservation for each motif are shown in table

We then compared other segments and found multiple vRNA5 (Table 5), vRNA7 (Table 6), and vRNA8 (Table 7) motifs that were proposed in different experimental environments. We found several highly conserved (sequence and structural conservation) motifs that were predicted in all or nearly all environments and strains: motifs in the region of 87–115 nt and 1527–1550 nt of vRNA5; 35–60 nt, 144–166 nt, 339–355 nt and 788–809 nt of vRNA7; 261–287 nt and 312–327 nt of vRNA8 (Tables 5, 6, 7). These highly conserved motifs are likely to play an important role during the replication cycle or may be involved in packaging nascent virions.

**Table 5:** Structural motifs predicted in vRNA5 in virio and in cellulo structures of A/California/04/2009 with base-pairing probabilities > 80% that were predicted also in previously established IAV vRNA5 structures probed in different environments [18, 24]

vRNA5								
IAV strain	A/California/04/2009			A/WSN/1933		A/Vietnam/1203/2004	Base-pairs conservation [%]	Nucleotide sequence conservation [%]
Probing environment	In virio	In cellulo	In vitro	In virio	Ex virio	In vitro		
Motif region [nt]	Base-pairing probability							
87–115	> 80%	> 80%	> 80%	< 10%	None	> 80% for 88–114 nt	98.77	96.22
238–254	none	> 80%	None	None	None	> 80%	93.55	91.09
349–366	> 80%	30–80%	None	30–80%	> 80%	None	88.01	93.62
974–988	> 80%	> 80%	> 80%	< 10% for 976–986 nt	30–80%	> 80%	96.84	89.52
993–1001	None	> 80%	> 80%	> 80%	< 10%	None	83.57	87.94
1038–1049	> 80%	> 80% for 1035–1051 nt	None	None	None	None	97.33	95.29
1076–1110	> 80%; for 1094–1099 nt 30–80%	> 80%	> 80%	None	None	> 80% for 1079–1109 nt	95.99	93.19
1129–1136	> 80%	30–80%	None	< 10%	None	None	88.58	87.28
1142–1160	> 80%	> 80%	None	30–80% for 1144–1158 nt	None	None	76.63	89.81
1193–1210	> 80%	> 80%	> 80%	> 80%	30–80%	None	80.46	88.05
1262–1275	> 80%	> 80%	None	30–80%	none	None	87.60	88.66
1372–1387	30–80%	> 80%	None	None	30–80%	None	96.87	94.69
1436–1446	30–80%	> 80%	None	None	none	None	89.92	90.83
1460–1522	> 80%	> 80%	> 80%	None	> 80%*	> 80%*	98.23	93.91
1527–1550	> 80%	> 80%	> 80%	> 80%	> 80%	30–80%	99.49	97.51

*Changes in sequence influenced the base-pairing

The base-pairing probability, as well as base-pairing and sequence conservation percentage values are indicated in the table. The conservation % values are rounded to the hundredth place

**Table 6:** Structural motifs predicted in vRNA7 in virio and in cellulo structures of A/California/04/2009 with base-pairing probabilities > 80% that were predicted also in previously established IAV vRNA7 structures probed in different environments [18, 35]

vRNA7							
IAV strain	A/California/04/2009		A/WSN/1933		A/Vietnam/1203/2004	Base-pairs conservation [%]	Nucleotide sequence conservation [%]
probing environment	In virio	In cellulo	In virio	Ex virio	In vitro		
Motif region [nt]	Base-pairing probability						
35–60	> 80%	30–80%	> 80%	> 80%	> 80%	99.91	95.85
64–78	> 80%	> 80%	> 80%	> 80%	None	97.81	96.41
144–166	> 80%	None	> 80%	> 80%	> 80%	99.18	90.04
250–300	None	> 80%	> 80% for 264–294 nt	> 80%	None	94.91	94.66
339–355	> 80%	None	> 80%	> 80%	> 80%	98.84	91.59
734–747	> 80%	> 80%	None	None	< 10%	91.21	92.32
762–786	> 80%	None	> 80%	None	> 80%	97.55	98.69
788–809	> 80%	> 80%	> 80%	> 80%	> 80%	99.77	99.80
845–862	> 80%	> 80%	None	None	> 80%	96.96	97.05
904–931	30–80%	> 80%	> 80% for 909–924 nt	30–80% for 909–924 nt	None	91.49	92.95
995–1004	30–80%	> 80%	None	30–80%	None	99.94	97.39

The base-pairing probability, as well as base-pairing and sequence conservation percentage values are indicated in the table

The conservation % values are rounded to the hundredth place

**Table 7:** Structural motifs predicted in vRNA8 in virio and in cellulo structures of A/California/04/2009 with base-pairing probabilities > 80% that were predicted also in previously established IAV vRNA8 structures probed in different environments [18, 33]

vRNA8																				
IAV strain		A/California/04/2009								A/WSN/1933					A/Vietnam/1204/2004		Base-pairs conservation [%]		Nucleotide sequence conservation [%]	
Probing environment		In virio		In cellulo		Cell lysate		In vitro		In virio	Ex virio		In vitro		In vitro					
Motif region [nt]		Base-pairing probability																		
42–62		none		> 80%		30–80%		> 80%		None	None		None		None		91.87		92.86	
65–80		> 80%		> 80%		30–80%		None		None	None		None		None		75.26		91.68	
261–288		> 80%		> 80%		> 80%		30–80%		> 80%	> 80%		> 80%		> 80%		97.87		94.63	
312–327		> 80%		> 80%		> 80%		> 80%		> 80%	> 80%		> 80%		> 80%		96.15		96.86	
380–393		30–80%		> 80%		> 80%		none		> 80%	> 80%		> 80%		none		97.50		91.01	
461–477		> 80%		> 80%		30–80%		None		> 80%	> 80%		> 80%		None		88.62		87.26	
645–666		> 80%		> 80%		30–80%		> 80%		none	> 80%		> 80%		None		78.69		79.87	
693–715		> 80%		None		30–80%		None		None	None		None		None		74.83		85.45	
751–763		> 80%		> 80%		None		None		> 80%	30–80%		None		> 80% for 736–748 nt		93.40		90.70	
768–789		> 80%		> 80%		None		None		30–80%	None		None		None		91.14		91.41	
793–813		> 80%		> 80%		> 80%		> 80% for 797–809 nt		30–80%; > 80% for 797–809 nt	30–80%		> 80%		None		87.76		85.53	
844–877		> 80%		> 80%		None		None		> 80%	> 80%		> 80%		None		94.45		94.55	

The base-pairing probability, as well as base pairing and sequence conservation percentage values are indicated in the table

The conservation % values are rounded to the hundredth place

### Amino acid sequence conservation in selected vRNA regions

Since some vRNA motifs are highly conserved despite having relatively low nucleotide sequence conservation, we analyzed the rate of nonsynonymous mutations in regions corresponding to these vRNA motifs, located within the coding region of vRNAs. The motifs were selected from Tables 4, 5, 6, 7 according to high structure conservation (≥ 96%) and lower nucleotide sequence conservation (< 96%). This analysis revealed several regions within vRNA1, 2, 3, and 5 containing synonymous mutations (Table 8; Supplementary E7). Interestingly, for vRNA5 region 1460–1522, vRNA7 regions 35–60 and 144–166, and vRNA8 region 261–288 the preservation of amino acid residues is comparatively low (Table 8; Supplementary E7). This indicates evolutionary pressure on the maintenance of these vRNA structural motifs which may play a role in the viral replication cycle.

**Table 8:** Amino acid conservation for vRNA structural motifs with ≥ 96% base-pairs conservation and < 96% nucleotide sequence conservation (selected from Tables 4, 5, 6, 7)

Segment	Motif region [nt]	Base-pairs conservation [%]	Nucleotide sequence conservation [%]	Amino acid sequence conservation [%]
vRNA1	1837–1851	97.10	93.45	99.36
vRNA2	621–638	96.04	88.92	99.57
vRNA3	457–465	97.20	88.42	99.90
vRNA5	974–988	96.84	89.52	99.63
	1076–1110	95.99	93.19	99.42
	1372–1387	96.87	94.69	98.54
	1460–1522	98.23	93.91	93.74
vRNA7	35–60	99.91	95.85	91.53
	144–166	99.18	90.04	84.77
vRNA8	261–288	97.87	94.63	87.71

## Discussion

RNAs are dynamic molecules that fold into many co-existing alternative structures of different abundances based on their biological environment and interactions with other RNAs and proteins [17, 52, 53]. During the replication processes, influenza RNA takes part in many essential cellular and viral interactions that alter its conformational landscape beyond of the virion [17, 54–56]. The study of this landscape is extremely difficult due to the low abundance of IAV vRNA compared to the total RNA isolated from cells. The application of standard methods based on capillary electrophoresis is not possible for in vivo studies. An opportunity has arrived with the establishment of new, highly sensitive NGS-based methodologies for RNA secondary structure probing such as Structure-seq and Structure-seq2, and methods such as SHAPE-MaP and DMS-MaPseq [26–28, 40, 57–59]. Since its inception, the MaP methodology has been used to resolve viral RNA structures for HIV, Chikungunya, SARS-CoV-2, Hepatitis C, Dengue, and Zika viruses [26, 60–66]. Recently, Dadonaite et al. used MaP to compare vRNA structures in the influenza virus A/WSN/33 strain under different conditions: in vitro, ex virio, and in virio [18]. Such data are a great source of knowledge concerning influenza virus vRNA structure.

Influenza evolution through antigenic drift and shift phenomena influences RNA sequence, which, in turn, can impact RNA secondary structure [67]. For this reason, a comparison of the vRNA secondary structures originating from different IAV strains might reveal RNA motifs that are potentially important for influenza. Our research concerning in vitro vRNA5 and vRNA8 structures showed that some motifs are conserved across distant IAV strains [24, 33, 34, 36]. These structures might fold differently in a biological context, as we showed recently in an example of the vRNA8 structure in cell lysate [37]. Moreover, a comparison by Dadonaite et al. between in vitro, ex virio, and in virio vRNA secondary structures showed that a limited number of similar structural motifs are preserved in a biological environment [18]. This may be the result of both RNA structural changes and RNA-RNA or RNA–protein interactions. A recent comparison between in virio and in cellulo SARS-CoV-2 RNA structures showed unique base-pair interactions while sharing many similar structural motifs [63]. Thus, apart from vRNA structure comparison and conservation between different influenza strains, it is important to study the folding in different environments, including in-cell probing.

Another argument in favor of in cellulo research is their use for the design of more effective RNA-targeting inhibitory methods. Inhibition efficiency depends on the target’s RNA secondary structure and accessibility in cells, and our research also supports this statement [23–25, 68]. Before this work, the folding of influenza vRNA in cells was largely unknown. Structural research concerning in cellulo folding is crucial to a better understanding of influenza virus biology, which will lead to new antiviral designs.

The careful analysis of structural data from in vivo RNA chemical mapping is key for the interpretation of biological processes and the application of that knowledge. We took into consideration previous approaches in the analysis of vRNA and our own experience in the determination and prediction of RNA structure. We used data originating from the chemical probing of two chemical reagents: NAI (SHAPE) and DMS. The major benefit is a more accurate structure prediction via the RNAstructure program [46, 69]. This is a step forward in methodology improvement, as in vivo mapping conventionally uses only one reagent [18, 26, 28, 60–62, 65, 70].

The interpretation of the RNA secondary structure in its biological context is challenging both experimentally and computationally and demands a different methodology for structure prediction compared to in vitro studies. We utilized an expanded global/local base-pairing approach, as the vast majority of identified viral RNA structures were predicted using a limited maximum base-pairing distance [18, 26, 60–63, 66, 70, 71]. Distance limitations reduce the computational load for structure prediction but cause data loss concerning long-distance intra-molecular interactions. Using our approach, long-range interactions were predicted. Such interactions were found within the viral RNA of different ssRNA viruses, including influenza [17, 18, 63, 65, 71, 72]. For example, one of the well-known intramolecular interactions of the confirmed structure–function relationship is the structure of the RdRp promotor that forms between the 5ʹ- and 3ʹ-ends of vRNA [18, 73]. Numerous other intrasegmental interactions are present in vRNAs [17, 18]. Base-pairing predictions that exclude such long-distance interactions may present a distorted picture, as many of these interactions take place in the authentic secondary structure. Thus, long-distance predictions need to be considered to ensure that all probable conformations can be properly assessed.

We decided to use different strategies while predicting vRNA structures in the RNAstructure program using experimental constraints. First, we used parameters based on nearest neighbor thermodynamic calculation for determining MFE structures with and without adding additional base-pair limitations (< 150 nt). This allowed us to detect both local structural motifs and long-distance interactions. Next, we performed partition function calculations, which were used to predict MEA structures in both local and global contexts (Fig. 2, Supplementary F1 Figs. S3–S7). The MEA structures are confirmed to have a higher positive predictive value (PPV), although they tend to maintain single-stranded regions [50]. For that reason, the MEA structure alone might result in a misleading conclusion about the degree of folding in RNA secondary structure predictions. Our dual approach using different RNA folding methods and constraints allowed us to exclude possible misinterpretations of the experimental data.

Our vRNA structure predictions in virio and in cellulo show that each vRNA segment forms locally folded motifs and long-range interactions. Notably, local and global structures predicted with the MEA or MFE algorithms share common structural motifs, indicating high thermodynamic stability in such regions. On the other hand, some motifs were unique for MFE structures (Fig. 2A, B; Supplementary F1 Figs. S3–S7), pointing to the importance of using different folding algorithms for secondary structure prediction. A diversified approach to structure prediction leads us to an interesting observation. A comparison of the PPV and sensitivity between MEA and MFE structures showed higher values for the global predictions and lower values for the local predictions in the case of the longer vRNA segments (Table 1). Meanwhile, the opposite trend was observed in shorter vRNAs, where sensitivity and PPV were higher in local structures and decreased with the RNA length. In the global vRNA structure predictions for shorter segments, sensitivity and PPV were lower in comparison to longer segments (Table 1). We observed many long-range interactions in all predicted global MEA and MFE in virio and in cellulo vRNA structures and these interactions exceeded the base-pair distance of the 150 nt.

When genomic RNA forms a vRNP complex, RNA is coiled on the protein core but is still partially exposed to the outside environment [15]. Independent research has shown that vRNAs in virio form unique and complex inter-and intra-segmental interaction networks [18, 34]. It was confirmed that specific regions within the vRNA (called packaging signals) are crucial for vRNP-vRNP interactions, influencing, among other factors, the appropriate assembly and packaging of all vRNPs set into the nascent virion [19, 21]. The RNA secondary structure of the packaging signals plays a key factor in maintaining the virulency of the influenza virus, and their disruption affects viral fitness [74]. In cells, vRNP complexes undergo structural changes resulting from diverse molecular processes [75]. There are indications that, despite differences in secondary structure, some relevant vRNA motifs might be present in both in virio and in cellulo environments. For the identification of common in virio and in cellulo motifs with functional potential, we compared our experimental probing data for vRNA segments 5, 7, and 8 (Table 3). CircleCompare analysis showed, with PPV values ranging from 64.95% (vRNA8 local MEA structures) to 37.66% (vRNA7 local MEA structures), a moderate similarity between the structures in different environments (Table 3). These common motifs might be engaged in interactions between different vRNA molecules, or might take part in intramolecular interactions to stabilize vRNP complexes [76].

Analysis of RNA secondary structures in vivo has some limitations, as chemical probing is affected not only by the RNA structure itself but by the interactions of the RNA with other biomolecules. Thus, a lack of nucleotide reactivity might be interpreted as a double-stranded region of RNA, while it could also be the result of some intermolecular interaction. Such ambiguity can lead to data misinterpretation, but can be largely avoided by performing additional sequence-structure conservation analysis. To this end, we calculated the base-pairing conservation (including compensatory and consistent mutations) among the IAV for each vRNA MEA and MFE structure, in global and local contexts (Supplementary E2-E5). In general, a similar average conservation of the whole structures (MEA, MFE, local, and global) is observed within each RNS segment, with some distinct highly conserved motifs (Fig. 4).

Independent research concerning the analysis of at least 35,000 influenza virus sequences for each vRNA showed that sequence conservation is different within particular vRNA segments [77]. Several conserved regions were observed in vRNA segments 1, 2, 3, 5, 7, and 8, but none in segments 4 and 6 [77]. Notably, the authors found that overall sequence conservation is different depending on the host. The most conserved segment was PB1 (vRNA2) for human strains, NS (vRNA8) for avian strains, and M (vRNA7) for swine influenza strains [77]. Our research concerning A/California/04/2009 strain (swine-origin) showed the highest average structural conservation in vRNA7 for both in virio (from 91.45 to 93.03%) and in cellulo (from 92.02 to 93.32%) structures. The lowest structural conservation was observed in vRNA4 and 6, segments which code for surface glycoproteins. This indicates the correlation between structural and sequence conservation, as these segments are evolutionally stimulated to mutate to preserve the virulency of influenza [78].

**Fig. 8:**
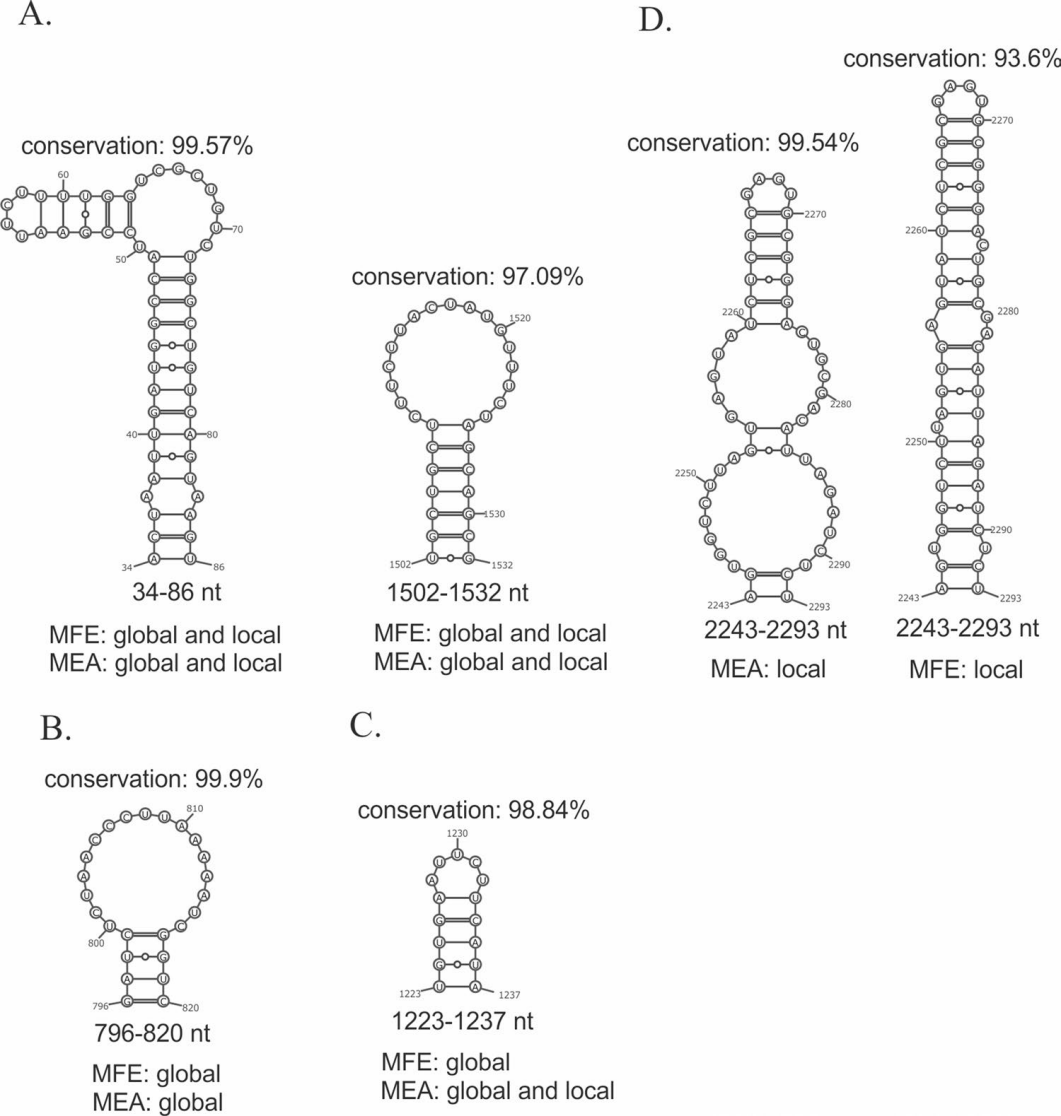
Differences and similarities in vRNA1 in virio between MEA and MFE structures predicted using global and local approach. **A** Highly conserved structural motifs predicted in all structures. **B** Highly conserved motif predicted in global structures only. **C** Highly conserved motif predicted in all structures apart from MFE local structure. **D** Motifs predicted in region 2243–2293 nt in MEA and MFE local structures

We determined many structural motifs of vRNA 1–3 and 5–8, which have ≥ 95% conservation between influenza strains (Supplementary E6 Tables 1 and 2). In the case of all predicted structures (MEA, MFE, local, and global) we found numerous highly conserved short-range interactions. Furthermore, we found a few long-range interactions in global predictions, for example helixes in global vRNA3 in virio structures (MEA and MFE) in regions 609–622/1754–1732 nt (conservation: 96.87%), 96–102/1784–1778 nt (conservation: 95.54%) and in global vRNA5 in cellulo structure (MEA and MFE) in region 40–44/1405–1401 nt (conservation: 96.96%). Interestingly, some highly conserved structural motifs were predicted in all structures (MEA, MFE), while some were exclusive to global, local, or MFE, MEA structures only. For example, in vRNA1 in virio (Fig. 8) we found two long hairpins in regions 34–86 nt and 1502–1532 nt in all structure predictions (Fig. 8A), while the nearly 100% conserved hairpin in region 796–820 nt was predicted in local and global MFE stuctures only (Fig. 8B). Next, the vRNA1 hairpin in region 1223–1237 nt was predicted in all structures apart from the local MFE structure (Fig. 8C), while region 2243–2293 nt was folded differently in the predicted MFE and MEA local structures (Fig. 8D).

In the case of vRNA4 and 6, we observed a limited number of highly conserved motifs (≥ 95%). Apart from a *panhandle* motif, the highest conservation was observed mostly in individual base pairs. Nonetheless, we found one highly conserved short hairpin in region 283–292 nt (conservation: 96.54%) in vRNA4 in virio in local folding prediction (Supplementary E6). Similarly, one short hairpin was observed in vRNA6 in virio in local folding prediction in region 19–28 nt (99.15%) (without a 21–26 pair in the MEA structure).

The comparison of the median low-SHAPE and low-Shannon entropy regions (Figs. 6 and 7; Supplementary F1 Figs. S12–20) allowed us to determine well-defined structural vRNA motifs (Fig. 5A, B). The highest number of motifs in the low-SHAPE and low-Shannon entropy regions were predicted in vRNA3 in virio and vRNA5 in cellulo (Fig. 5). Notably, some motifs in low-SHAPE, low-Shannon entropy regions were common for in virio and in cellulo predictions: vRNA5 hairpins in regions 87–115 nt, 974–988 nt, 1193–1210 nt, and 1460–1522 nt; vRNA7 hairpins in regions 64–78 nt, 788–809 nt, and 845–862 nt; and vRNA8 hairpins in regions 65–80 nt, 266–283 nt, 751–763 nt, and 768–788 nt. Many of the predicted motifs in low-SHAPE, low-Shannon entropy regions had high base-pairs conservation among IAV (Fig. 5), indicating their possible functional importance.

Our previous research concerning vRNA5, 7, and 8 in vitro and vRNA8 in cell lysate suggested multiple conserved structural RNA motifs that may play roles during the viral replication cycle and that might be preserved in virio and in cellulo [24, 33–35, 37]*.* Additionally, the first genome-wide analysis of in vitro, ex virio, and in virio structures of A/WSN/33 (H1N1) vRNA segments was published [18], and we proposed vRNA5 and vRNA8 secondary structures that correlated with this independent study [18, 24, 34, 36].

Detailed analysis of segments 5, 7, and 8 (Tables 5, 6, 7) confirmed many motifs in vitro, in virio, and ex virio within the same IAV strain, as well as in the different and distant IAV strains (H5N1) [18, 24, 33–36]. Many of the motifs had high base-pairs conservation. Based on previous study we did not expect significant sequence covariation (structure supporting mutation) in the motifs [79, 80]. Interestingly, we observed higher base-pairs conservation of the motifs located at both the 5ʹ- and 3ʹ-ends of these segments. Notably, these regions were indicated to serve as possible packaging signals for IAVs [19]. In the case of vRNA5, we observed two motifs in regions 87–115 nt (conservation: 98.77%) and 1527–1550 nt (conservation: 99.49%) that were found in different IAV strains, including the H5N1 subtype (Table 5). These two motifs were proposed as packaging signals in our previous in vitro study [34]. In vRNA7, we found two motifs (Table 6) in regions 35–60 nt and 788–809 nt that are nearly 100% conserved within IAV (99.9% and 99.77%, respectively). These two motifs had been predicted in an independent in silico study [81]. In the same study, the hairpin motif formed in region 64–78 nt was also indicated as highly conserved within IAV [81]. We also found two motifs in the vRNA8 structure (Table 7) that were predicted in all compared vRNA8 structures in regions 261–288 nt and 312–327 nt, and these two had the highest base-pairs conservation in this dataset (97.87% and 96.15%, respectively). Some other motifs were common to the H1N1 subtype, such as motifs of vRNA5 (974–988 nt, 993–1001 nt, and 1193–1210 nt), vRNA7 (64–78 nt and 264–294 nt), and vRNA8 (793–813 nt) [18, 24, 33–36].

The least studied influenza vRNA structures are within segments 1, 2, 3, 4, and 6, with only data of A/WSN/33 being available [18]. We observed a limited number of the structural motifs predicted (with base-pairing probability >80%, calculated according to RNA structure) in our in virio structures that were also predicted in the study concerning A/WSN/33 strain in virio (Table 4). In the case of segments 1, 2, and 3, we found that common structural motifs were also highly structurally conserved across all IAV strains. Three common RNA hairpins were almost 100% conserved: vRNA1 motif in region 50–63 nt (99.88%); vRNA2 motifs in region 79–93 nt (99.95%) and 2301–2311 nt (99.84%). Although both strains represent the same influenza subtype (H1N1), only a few motifs were common for vRNA4 (regions: 133–156 nt, 637–657 nt) and vRNA6 (regions: 693–703 nt, 832–852 nt) (Table 4).

Multiple studies suggest that biologically functional RNA motifs exist in regions of strong primary sequence conservation and that suppression of synonymous codon usage may be a result of maintaining RNA structure [82–84]. Additionally, the introduction of synonymous mutations within conserved regions of vRNA packaging signals has been shown to dramatically reduce genome assembly [83, 85, 86]. As another example, the structural element formed in the conserved nucleotide region 30–90 of vRNA1 (in our structure 34–86 nt, Fig. 5) has been determined as the genome packaging signal for IAV [84]. Synonymous mutations to disrupt this motif significantly affected PB2 segment packaging efficiency; however, compensatory mutations (synonymous and nonsynonymous) restoring the motif rescued PB2 segment packaging [84]. This shows the key role of the vRNA structure in the viral life cycle. In conclusion, since RNA structure constraints appear to suppress sequence variation, RNA motifs of high structural and sequential conservation, such as 79–93, 2301–2311 (vRNA2, Table 4); 1527–1550 (vRNA5, Table 5); 788–809, and 995–1004 (vRNA7, Table 6) have great functional potential. These motifs are all located in regions identified as terminal packaging signals for IAV genome assembly (reviewed in [87]).

The exact relationship between the vRNA secondary structure and the role it plays in intersegmental interactions in virions and cells remains a mystery. Independent research has confirmed the existence of intersegmental vRNA-vRNA interactions in virio for IAV and reported that these interactions are strain-dependent. It is worth mentioning that so far no such studies have been published on the strain A/California/04/2009. Le Sage et al. [17] revealed a complex network of intersegmental interactions for the strain A/WSN/1933. The authors indicated some regions within the vRNA that are likelier to interact than others, and they called these regions “hot spots.” The highest number of intersegmental interactions was detected between vRNA5 and vRNA6, while vRNA5 is the segment responsible for the largest number of all interactions. One of the most active “hot spots” within vRNA5 is the region 632–706 nt [17]. We did not find any conserved motif in this vRNA5 fragment (Table 5). Moreover, the in virio MEA global structure in this region is predominantly unfolded, whereas the global MFE structure features a single hairpin at 664–697 nt (with rather low base-pairs conservation for IAV equal to 88.53%). The four motifs present in virio and analyzed in Table 5 are located in regions that form intersegmental interactions within A/WSN/1933: 87–115 nt, 974–988 nt, 1460–1522 nt, and 1527–1550 nt [17]. Importantly, the 1460–1522 nt motif is highly structurally conserved (98.23%) despite the lower nucleotide and amino acid conservation (93.91% and 93.74%, respectively; Table 8, Supplementary E7) which may indicate evolutionary pressure on the preservation of this RNA structural element. Interestingly, the 1460–1522 nt motif was identified in a region proposed by Dadonaite et al. as liable for interaction between vRNA5 and vRNA2 [18]. The rest of the analyzed motifs were identified outside the regions involved in intersegmental interactions.

In A/WSN/1933 vRNA6, the “hot spot” region 825–890 nt is responsible for interacting with vRNA1, vRNA2, and vRNA3 [17]. In this region, we found a motif 832–852 nt, which is common for A/WSN/1933 and A/California/04/2009 strains (Table 4, Supplementary F2). However, within different IAV strains the base-pairing conservation of this motif is very low. Upstream of this “hot spot” region, we found another motif—693–703 nt with a high base-pairing conservation equal to 91.11% (Table 4, Supplementary F2). The region 115–144 nt of vRNA6 proposed for interaction with vRNA5 “hot spot” in A/WSN/1933 [17] is relatively poorly structured in both MEA and MFE A/California/04/2009 structures. We found one motif in this region (107–139 nt), however, it has a very low probability of base-pairing (calculated by RNAstructure). Interestingly, the interaction between 686–711 nt of vRNA5 and 115–144 nt of vRNA6 shown by Le Sage et al., can occur in A/California/04/2009 but with slight differences in the base-pairing due to the sequence variation in this region [17]. A region 40–260 nt of vRNA6 was found to interact with vRNA1, vRNA2, vRNA3, and vRNA5 [17]. In our structural study, we found several small hairpins and motifs within this region but these have rather low base-pairing probability as well as structural conservation. It is worth noting that this may be a result of evolutionary pressure to preserve intersegmental interactions, especially since the 40–260 nt region has been shown in previous research to be a 5ʹ end packaging signal [19, 87]. Two regions 40–255 and 1340–1405 nt of vRNA3 were identified as taking part in vRNA-vRNA interactions in A/WSN/1933 [17]. The interaction between vRNA3 43–93 nt and vRNA5 662–703 nt was proposed by Le Sage et al. [17], and the interaction between vRNA3 102–133 nt and vRNA4 831–859 nt was proposed by Dadonaite et al. [18]. Interestingly, our data revealed structurally conserved hairpins in 56–89 nt and 666–686 nt regions of vRNA3 (with a base-pairing conservation equal to 96.11% and 99.88%, respectively), and these may be involved in the intersegmental interactions.

Previous research has shown that vRNA7 and vRNA8 are involved in intersegmental interactions but to a lower extent than vRNA3, vRNA5, and vRNA6 [17, 18]. Dadonaite et al. showed that the 605–780 nt region of vRNA8 in A/WSN/1933 can interact with vRNA1, vRNA3, and vRNA7 [18]. Further research on A/WSN/1933 by Le Sage et al. has indicated interactions between 500–550 nt and 800–850 nt regions of vRNA8 and multiple regions of vRNA5 [17]. Four motifs (645–666, 693–715, 751–763, and 768–788 nt) were identified in the first region, and two (793–813 and 844–877 nt) in the second region (Table 7). Two motifs are highly conserved for IAV: 751–763 and 768–788 nt (with a base-pairing conservation equal to 93.40% and 90.81%, respectively). Presumably, these motifs and the unpaired regions between them are involved in intersegmental interactions in virio. There is another motif between these highly interactive regions, located in 719–782 nt of the vRNA8, which is highly conserved for IAV (93.6% base pairs conservation). This motif was predicted in the in virio global MEA structure (Supplementary F2) and was described in our previous in vitro study [36]. A majority of the motifs identified in vRNA7 and vRNA8 of A/California/04/2009 were found outside the regions identified previously as engaging in vRNA-vRNA interactions [17, 18]. However, a few motifs of vRNA7 (35–60 nt, 144–166 nt, 845–862 nt, and 904–931 nt) with high structural conservation (ranging from 91.49 to 99.91%) may take part in the intersegmental interactions (Table 6). The motifs 35–60 and 144–166 nt in the 5ʹ-end and 845–862 and 904–931 nt in the 3ʹ-end of vRNA7 are located in regions previously suggested as packaging signals [35]. Interestingly, two highly conserved (> 99%) structures, 35–60 nt and 144–166 nt, have relatively low nucleotide and amino acid conservation (nt: 95.85% and 90.04%, respectively; AA: 91.53% and 84.77% respectively; Table 8, Supplementary E7). This suggests evolutionary pressure on maintaining the RNA secondary structure, which may be biologically functional. The regions 380–430 and 550–580 nt of vRNA7 in strain A/WSN/1933 were reported to be involved in vRNA-vRNA interactions [17], but no significant motif was found in this region for A/California/04/2009 in our prediction.

Our predictions of the global in virio and in cellulo vRNA structures show many long-range intrasegmental base-pairing. Such long-range interactions, at least in virio, are in agreement with the previously established intrasegmental interaction network proposed in crosslinking experiments [17, 18]. Previous research has shown, that the *panhandle* interaction between the 5ʹ- and 3ʹ-end of vRNAs is not the only intrasegmental interaction and many others can take place, especially for segments 1, 2, 3, 5, 6, and 7 [17, 18]. Interestingly, these findings also show a coexistence of intersegmental and intrasegmental interactions within the same vRNA regions [17, 18] This indicates great flexibility of vRNA structures and interactions during the virion packaging processes. This flexibility has been validated using synonymous mutations of “hot spot” regions in several different strains [17, 18].

Lee et al. demonstrated that NP binding with vRNA of both A/California/04/2009 and A/WSN/1933 strains in virio is generally uneven [15]. In addition, the NP binding profile is unique to each vRNA segment, and NP-rich regions are usually located at the ends and in the middle of the segments. The comparison between in virio vRNA secondary structure of A/California/04/2009 with the binding profile of NP revealed that NP binding occurs independently of the RNA duplex formation, both in highly structured regions and intersegmental “hot spots” [15, 17]. Several conserved motifs possibly involved in intersegmental interactions were identified in NP-rich regions, like motifs 87–115, 657-681 ("hot-spot"), 974–988, 1460–1522, and 1527–1550 nt of vRNA5; 35–60, 845–862 and 904–931 nt of vRNA7; motifs 475–620; 645–666, and 768–788 nt of vRNA8. Alternatively, some highly structured RNA were identified as NP-poor, like 693–715 and 751–762 nt motifs of vRNA8 and 1125–1425 nt motifs of vRNA5 (Table 5, 6 and 7, Supplementary E6). Several other conserved motifs found in regions that do not take part in RNA-RNA interactions belong to both NP-rich and NP-poor RNA. For example, two vRNA8 motifs (261–288 and 793–813 nt) are predicted in the NP-poor regions, while one highly conserved motif (312–327 nt) in vRNA8 is predicted in the NP-rich region. Other examples are motifs in vRNA5 (1076–1110 nt) and vRNA7 (64–78 nt) predicted in NP-rich regions and the vRNA7 motif (762–786 nt) predicted in the NP-poor region (Tables 5, 6 and 7, Supplementary E6) [18, 24, 33, 35, 37, 88].

Interactions between vRNA segments can occur in cells [89]. This is an indication for further studies of the vRNA motifs with a particular emphasis on motifs common in virio and in cellulo. It is worth noting, that many motifs that were exclusively predicted in virio might be particularly critical for intersegmental interactions. Also, many RNA-RNA interactions might be dynamic. They could, for example, disrupt individual motifs by binding to surrounding single-stranded regions and thereby unfolding existing base-pairing. Finally, the structural conservation of the motifs may be related to unknown functions that need to be investigated for a better understanding of the virus biology.

In summary, RNA secondary structure of the IAV genome of strain A/California/04/2009 (H1N1) was determined in virio and in cellulo. Our research showed that despite differences between the vRNA secondary structures of individual IAV strains, some structural motifs are common and highly conserved across distant strains. Notably, for the first time, we showed a vRNA secondary structure in living cells. Moreover, we highlighted potentially functional structural motifs that exist in virio and in cellulo environments. These motifs are of interest for further investigation, as they may play key roles in the viral replication cycle. The knowledge gained in this research may also be used to design more specific RNA-targeting inhibitors, focusing on vRNA motifs universal to all IAV strains.

## Supplementary Information

Below is the link to the electronic supplementary material.

Supplementary file1 (XLSX 2673 KB)

Supplementary file2 (XLSX 453 KB)

Supplementary file3 (XLSX 565 KB)

Supplementary file4 (XLSX 450 KB)

Supplementary file5 (XLSX 534 KB)

Supplementary file6 (XLSX 32 KB)

Supplementary file7 (XLSX 147 KB)

Supplementary file8 (PDF 4784 KB)

Supplementary file9 (PDF 17363 KB)

## Data Availability

Raw sequencing data from chemical probing experiments and quality reports from ShapeMapper2 are available in the Zenodo repository (https://doi.org/10.5281/zenodo.7266983), https://doi.org/10.5281/zenodo.7266983. Supplementary Data are available at Cellular and Molecular Life Sciences online.
