# Supplementary material for: In vivo secondary structural analysis of Influenza A virus genomic RNA
Source: Cell Mol Life Sci. 2023 May 2;80(5):136. doi: 10.1007/s00018-023-04764-1 (PMC10153785; doi:10.1007/s00018-023-04764-1)
Supplement: Supplementary file 8 — Supplementary file8 (PDF 4784 KB) [file 18_2023_4764_MOESM8_ESM.pdf]

Supplementary F1

In vivo secondary structural analysis of Influenza A virus genomic RNA

Barbara Mirska[1], Tomasz Woźniak[2], Dagny Lorent[1], Agnieszka Ruszkowska[1], Jake M. Peterson[3], Walter N. Moss[3], David H. Mathews[4], Ryszard Kierzek[1] and Elzbieta Kierzek[1*]

[1] Institute of Bioorganic Chemistry, Polish Academy of Sciences, Noskowskiego 12/14, 61-704 Poznan, Poland

[2] Institute of Human Genetics, Polish Academy of Sciences, Strzeszynska 32, 60-479 Poznan, Poland

[3] Roy J. Carver Department of Biophysics, Biochemistry and Molecular Biology, Iowa State University, Ames, IA 50011, USA

[4] Department of Biochemistry & Biophysics and Center for RNA Biology, 601 Elmwood Avenue, Box 712, School of Medicine and Dentistry, University of Rochester, Rochester, NY 14642, USA

*elzbieta.kierzek@ibch.poznan.pl

Supplementary F1

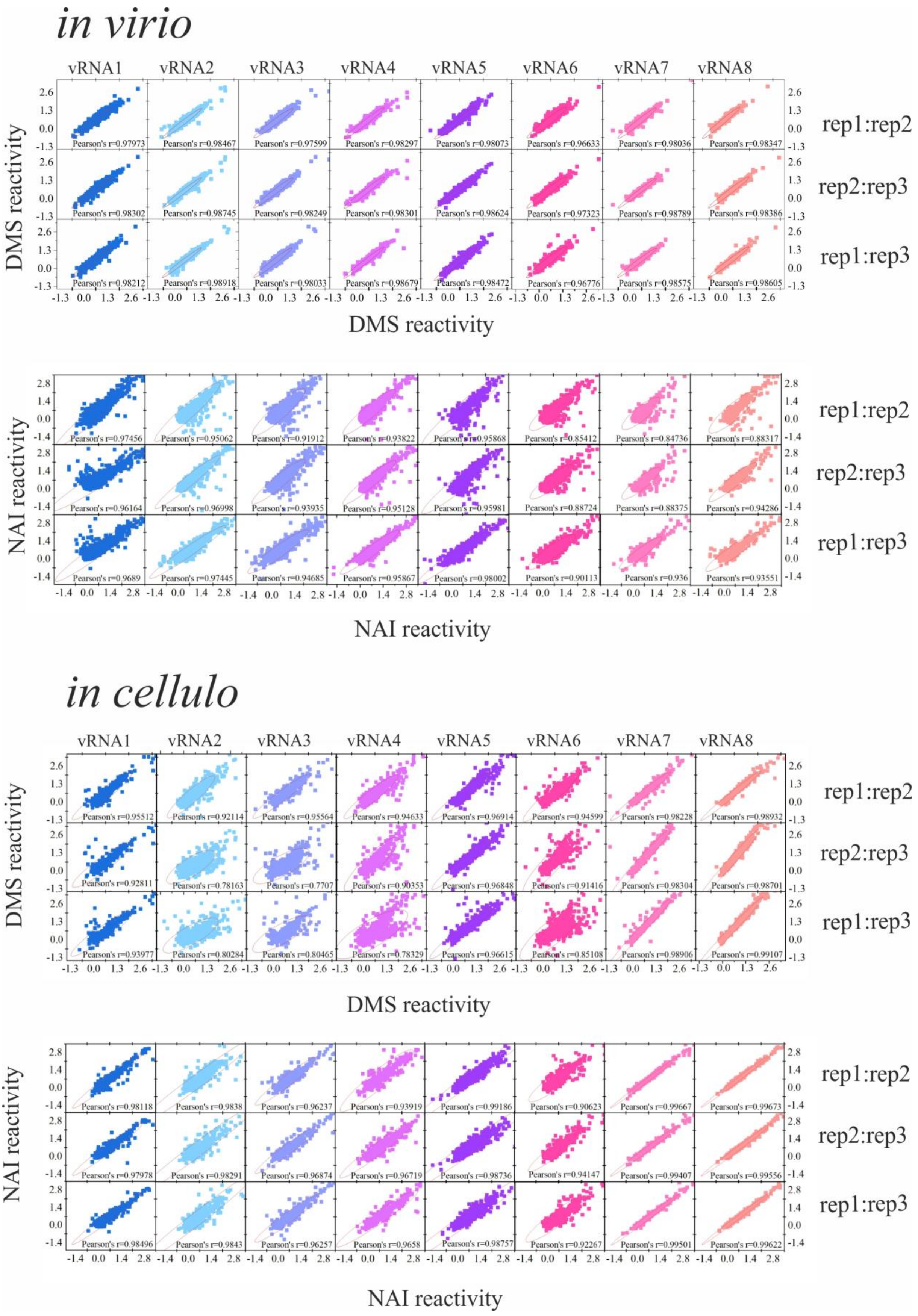

Figure S1. Pearson Correlation Coefficient (PCC) calculated between three independent biological replicates from the *in virio* and *in cellulo* experiments. The PCC r value is indicated on each plot.

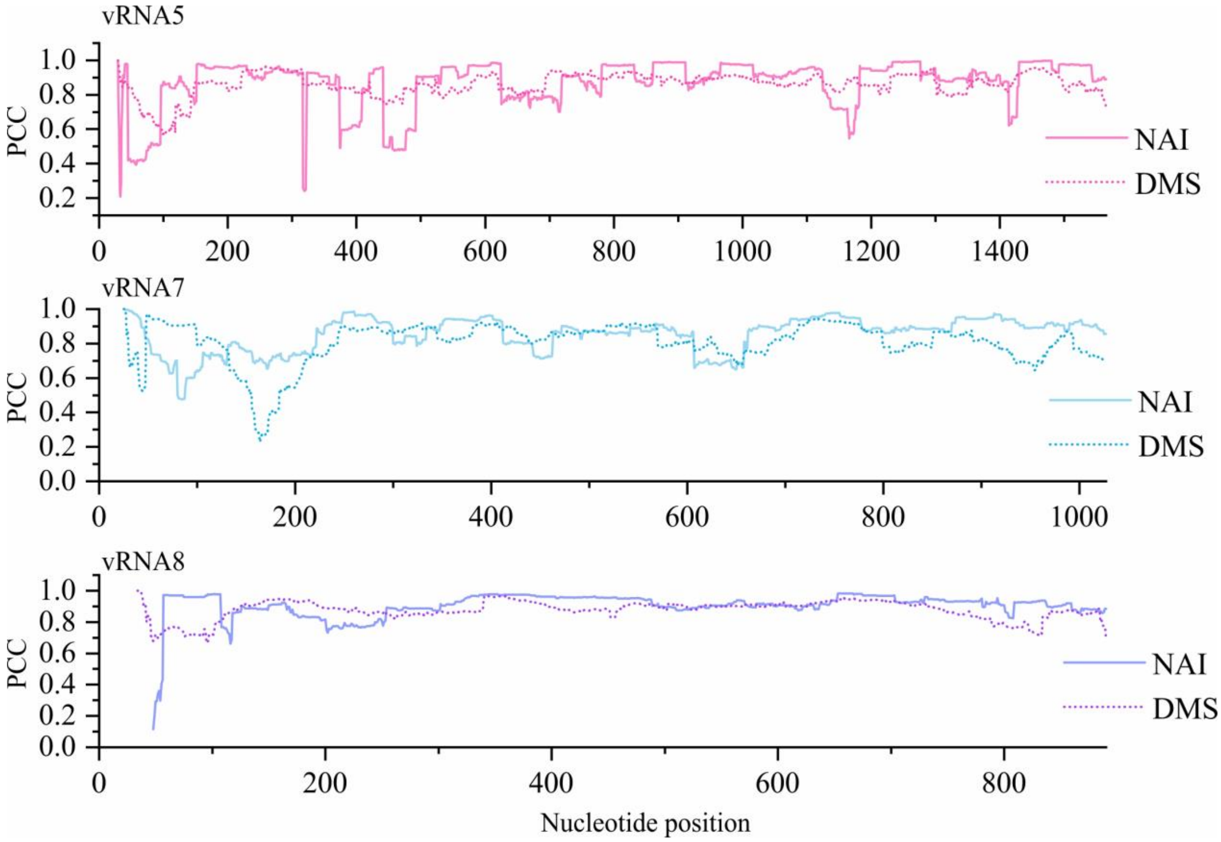

Figure S2. PCC calculated over 50 nt window, between *in virio* and *in cellulo* SHAPE and DMS reactivities. High coefficients indicate regions of possible conservation of the structure, while low coefficient regions of different structure *in virio* and *in cellulo*.

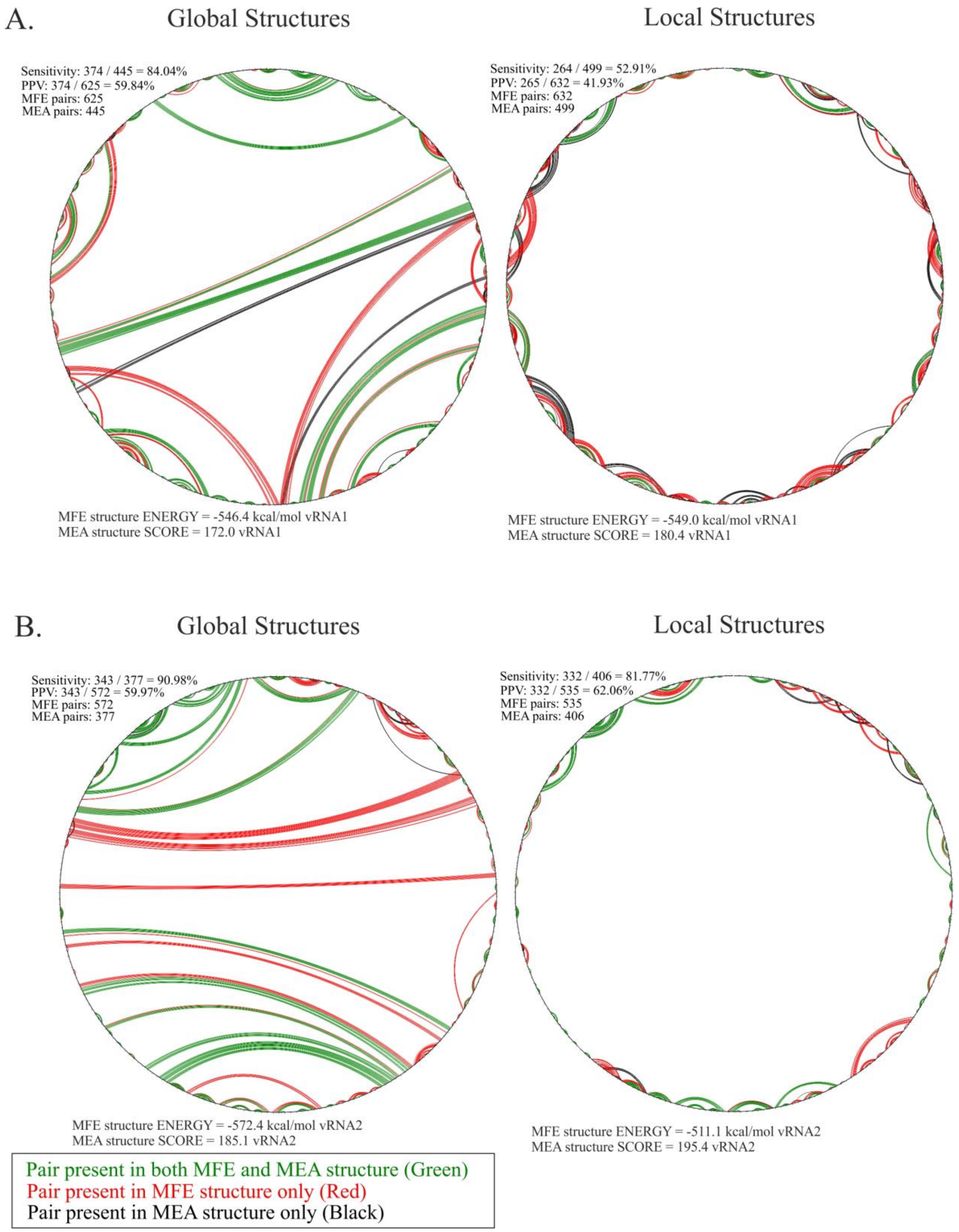

Figure S3. Comparison via CircleCompare tool (RNAstructure) between MFE and MEA structures of vRNA1 and vRNA2 predicted *in virio* in global and local context. The comparison show similarities (green) and differences (red) in the base-pairing between the MFE and MEA structure predictions. In MEA, the score is = 2*sum(Pij) + sum(Pk) where Pij is the probability for the base pair i-j for all pairs and Pk is the probability that k is unpaired for all unpaired nucleotides. **A.** Comparison between MFE and MEA global and local predictions for vRNA1 **B.** Comparison between MFE and MEA global and local predictions for vRNA2 .

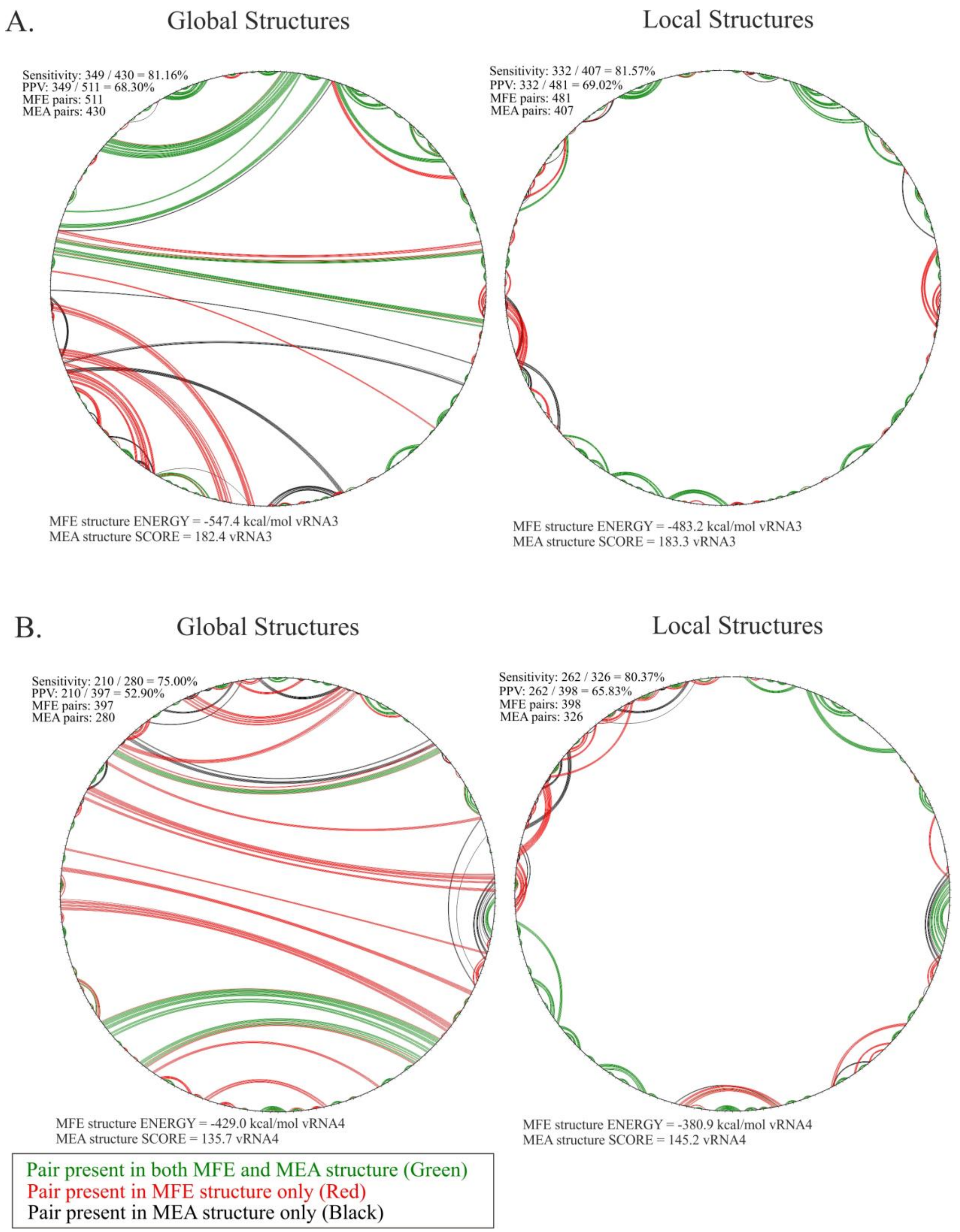

Figure S4. Comparison via CircleCompare tool (RNAstructure) between MFE and MEA structures of vRNA3 and vRNA4 predicted *in virio* in global and local context. The comparison show similarities (green) and differences (red) in the base-pairing between the MFE and MEA structure predictions. In MEA, the score is = 2*sum(Pij) + sum(Pk) where Pij is the probability for the base pair i-j for all pairs and Pk is the probability that k is unpaired for all unpaired nucleotides. **A**. Comparison between MFE and MEA global and local predictions for vRNA3 **B**. Comparison between MFE and MEA global and local predictions for vRNA4.

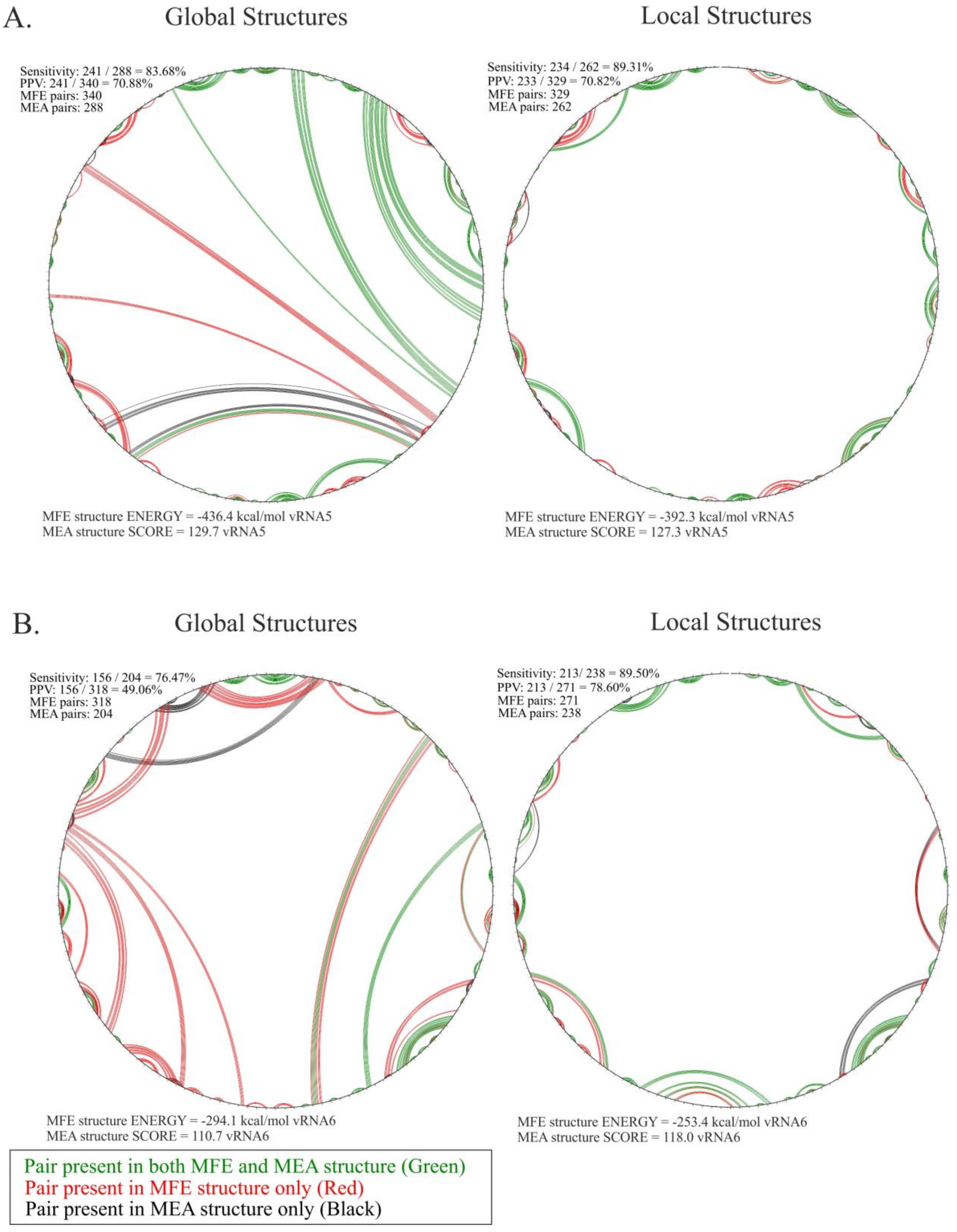

Figure S5. Comparison via CircleCompare tool (RNAstructure) between MFE and MEA structures of vRNA5 and vRNA6 predicted *in virio* in global and local context. The comparison show similarities (green) and differences (red) in the base-pairing between the MFE and MEA structure predictions. In MEA, the score is = 2*sum(Pij) + sum(Pk) where Pij is the probability for the base pair i-j for all pairs and Pk is the probability that k is unpaired for all unpaired nucleotides. **A.** Comparison between MFE and MEA global and local predictions for vRNA5 **B.** Comparison between MFE and MEA global and local predictions for vRNA6.

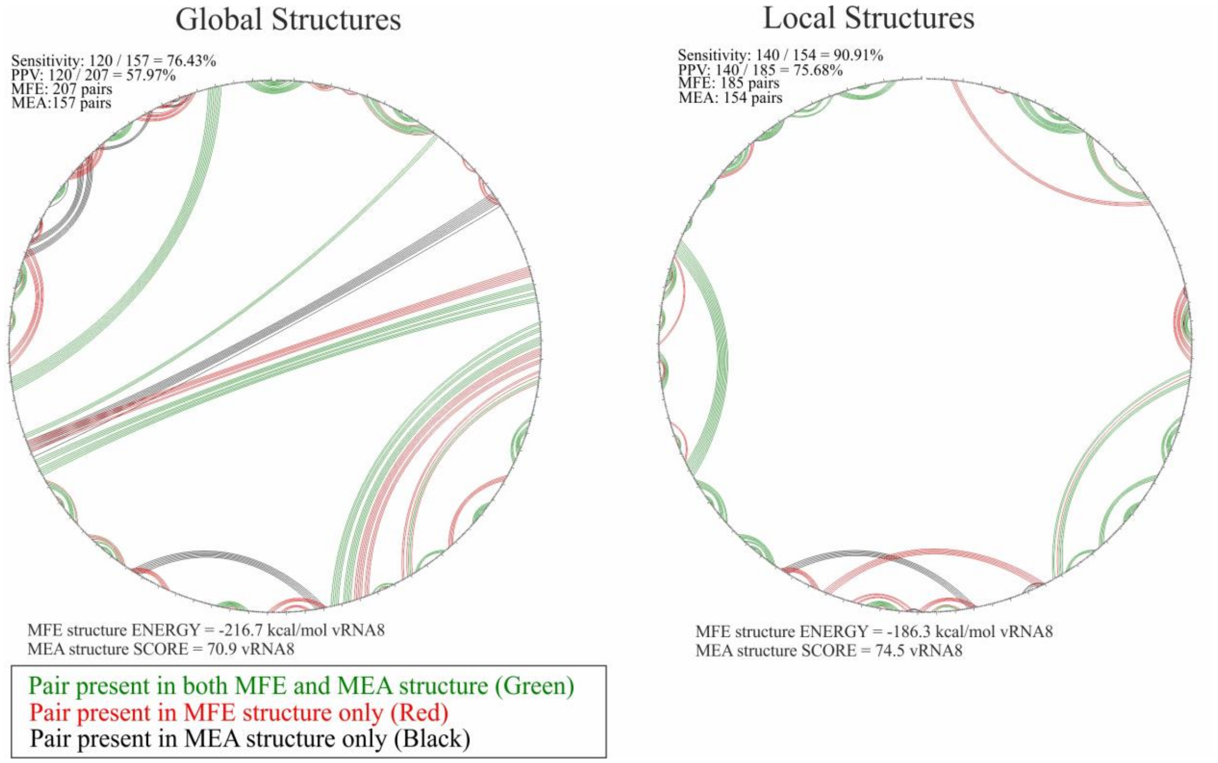

Figure S6. Comparison via CircleCompare tool (RNAstructure) between MFE and MEA structures of vRNA8 predicted *in virio* in global and local context. The comparison show similarities (green) and differences (red) in the base-pairing between the MFE and MEA structure predictions. In MEA, the score is = 2*sum(Pij) + sum(Pk) where Pij is the probability for the base pair i-j for all pairs and Pk is the probability that k is unpaired for all unpaired nucleotides.

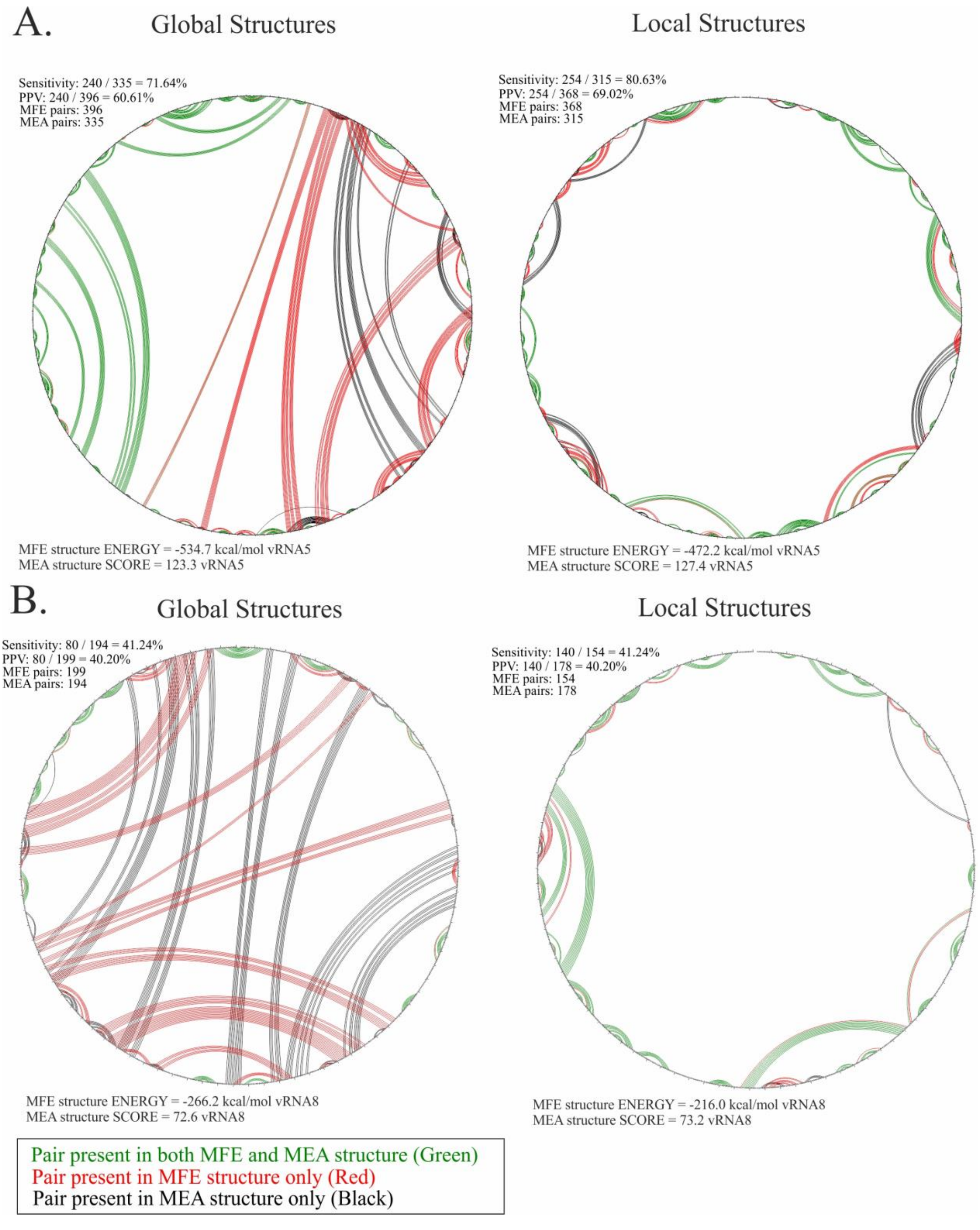

Figure S7. Comparison via CircleCompare tool (RNAstructure) between MFE and MEA structures of vRNA5 and vRNA8 predicted *in cellulo* in global and local context. The comparison show similarities (green) and differences (red) in the base-pairing between the MFE and MEA structure predictions. In MEA, the score is = 2*sum(Pij) + sum(Pk) where Pij is the probability for the base pair i-j for all pairs and Pk is the probability that k is unpaired for all unpaired nucleotides. **A**. Comparison between MFE and MEA global and local predictions for vRNA5 **B**. Comparison between MFE and MEA global and local predictions for vRNA8.

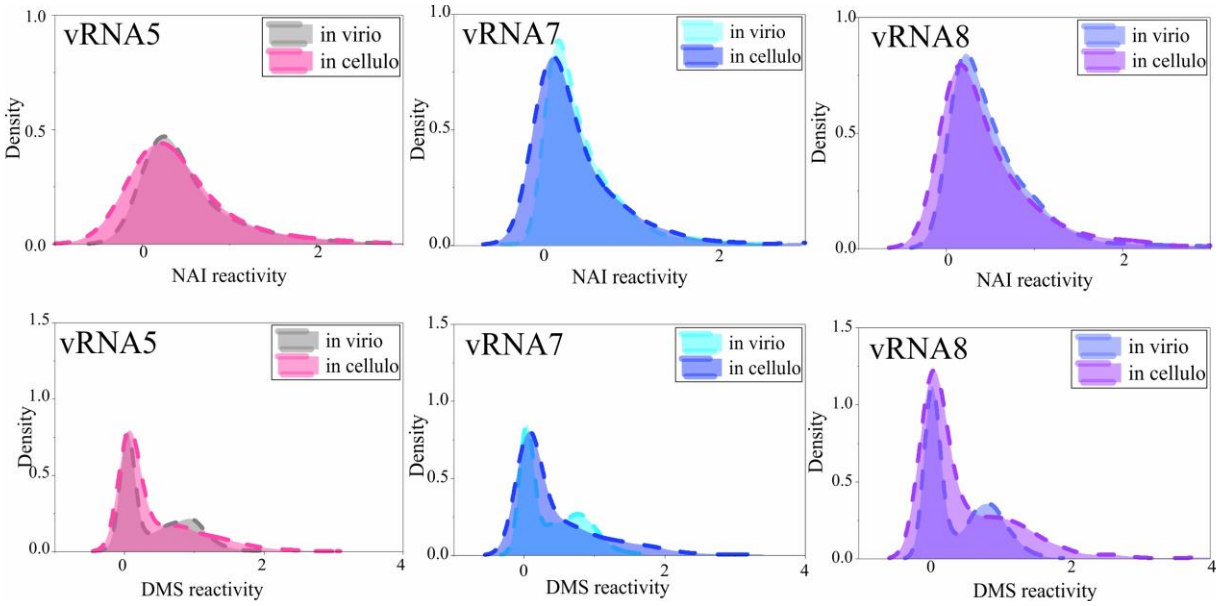

Figure S8. SHAPE and DMS reactivity density plots *in virio* and *in cellulo.*

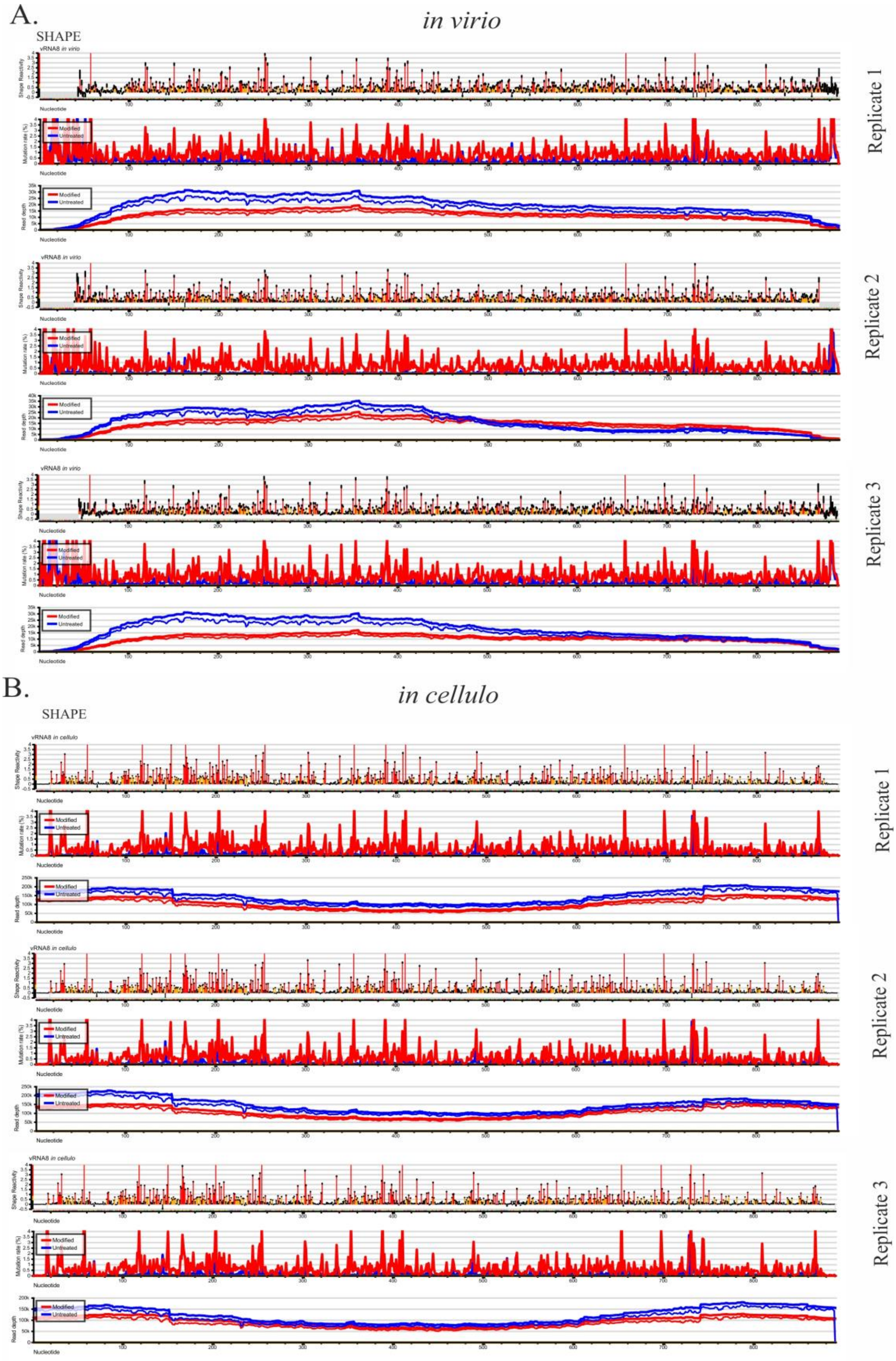

Figure S9. Reactivity profiles of NAI (SHAPE) probing of segment 8 vRNA in three independent biological replicates. **A**. Reactivity profiles (NAI) for vRNA8 from *in virio* chemical probing experiment **B.** Reactivity profiles (NAI) for vRNA8 from *in cellulo* chemical probing experiment.

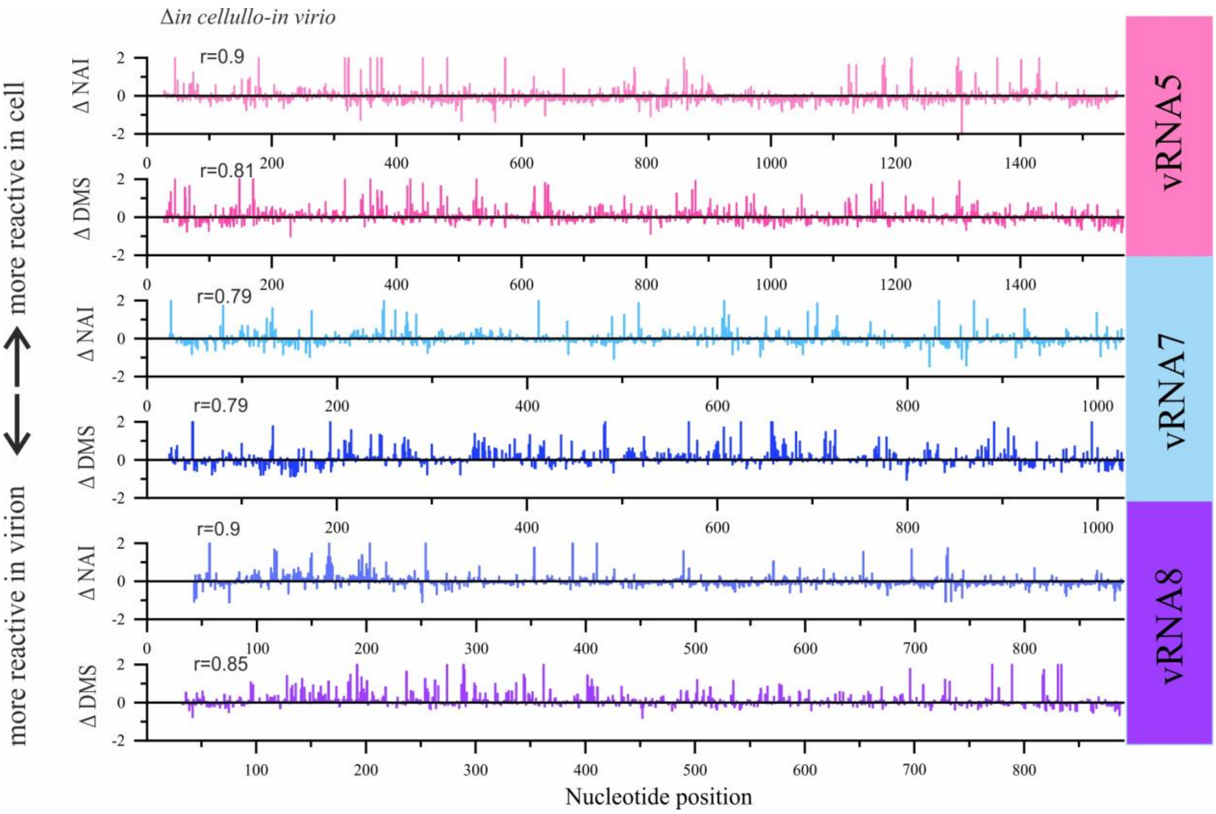

Figure S10. Calculation of single-nucleotide reactivity difference between *in cellulo* and *in virio* probing experiment for segments vRNA5, 7 and 8. The difference ΔDMS and ΔNAI were calculated by subtracting *in virio* reactivities from *in cellulo* reactivities. The same scale was used for every plot. The values above 0 indicates higher reactivities in *in cellulo*, while values below 0 indicate reactivities higher in *in virio* conditions.

18S rRNA

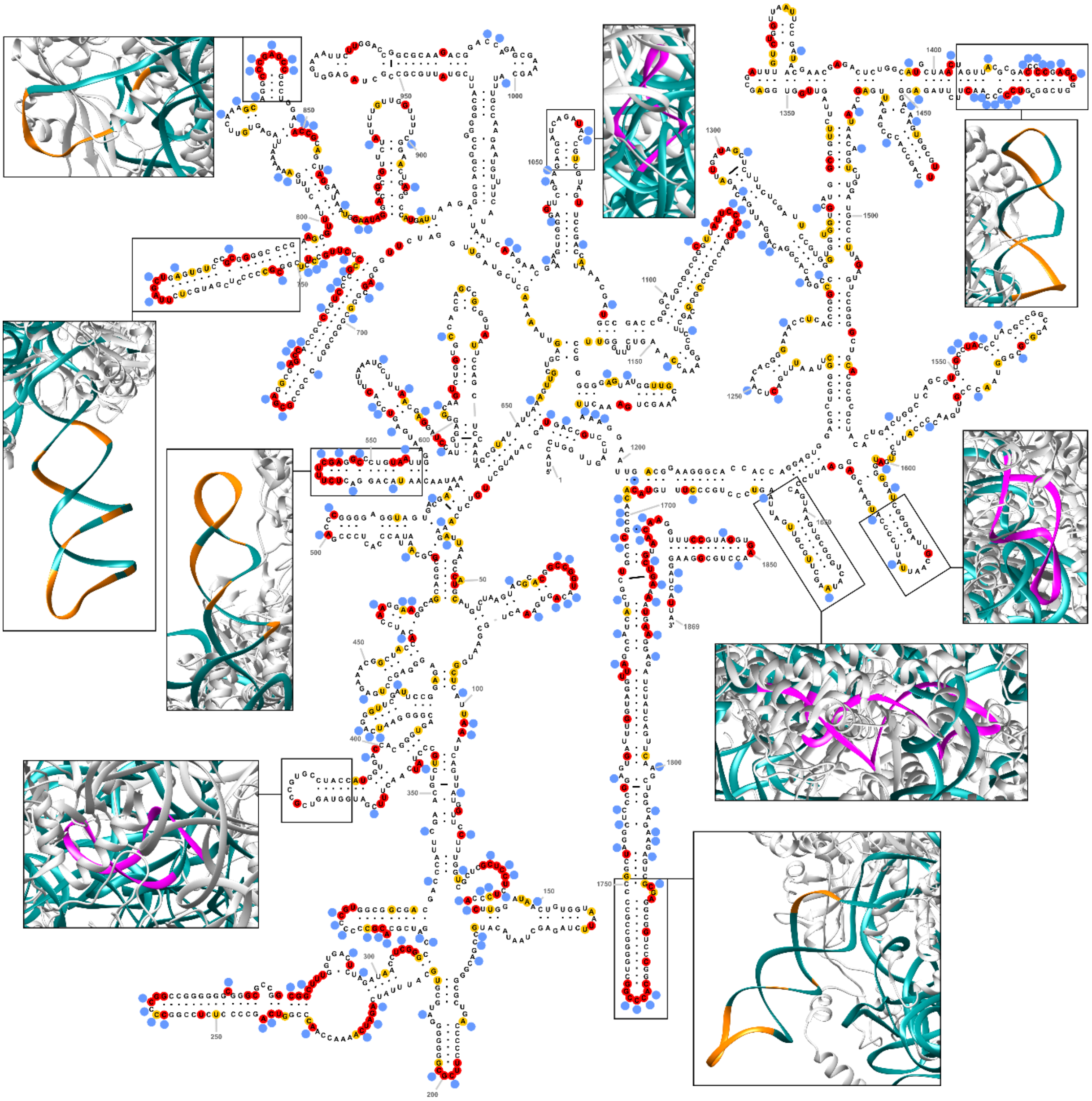

Figure S11. Correlation of chemical modifications in 18S rRNA (detected by SHAPE-MaP) with the 2D and 3D structures of 18S rRNA. The chemically mapped sites are marked on the secondary structure of 18S rRNA from the RNAcentral database, ID: URS00005A14E2_9606 (https://rnacentral.org/rna/URS00005A14E2/9606). Strong (reactivity>0.85)and medium (reactivity 0.5-0.85) SHAPE modifications are marked by red and yellow circles, respectively. Blue circles indicate strong DMS modifications (reactivity >0.85). Selected regions of the 18S rRNA secondary structure (black rectangles) are shown in 3D representation based on the Cryo-EM structure of the 80S ribosome (gray, PDB ID: 4v6x). 18S rRNA (chain B2) is sea green, whereas the modification sites (circles on the 2D structure) are marked in orange in the 3D structure. Solvent-inaccessible regions (single-stranded but buried in the 3D structure) are in magenta. The 3D structure was analyzed and presented using the Chimera software

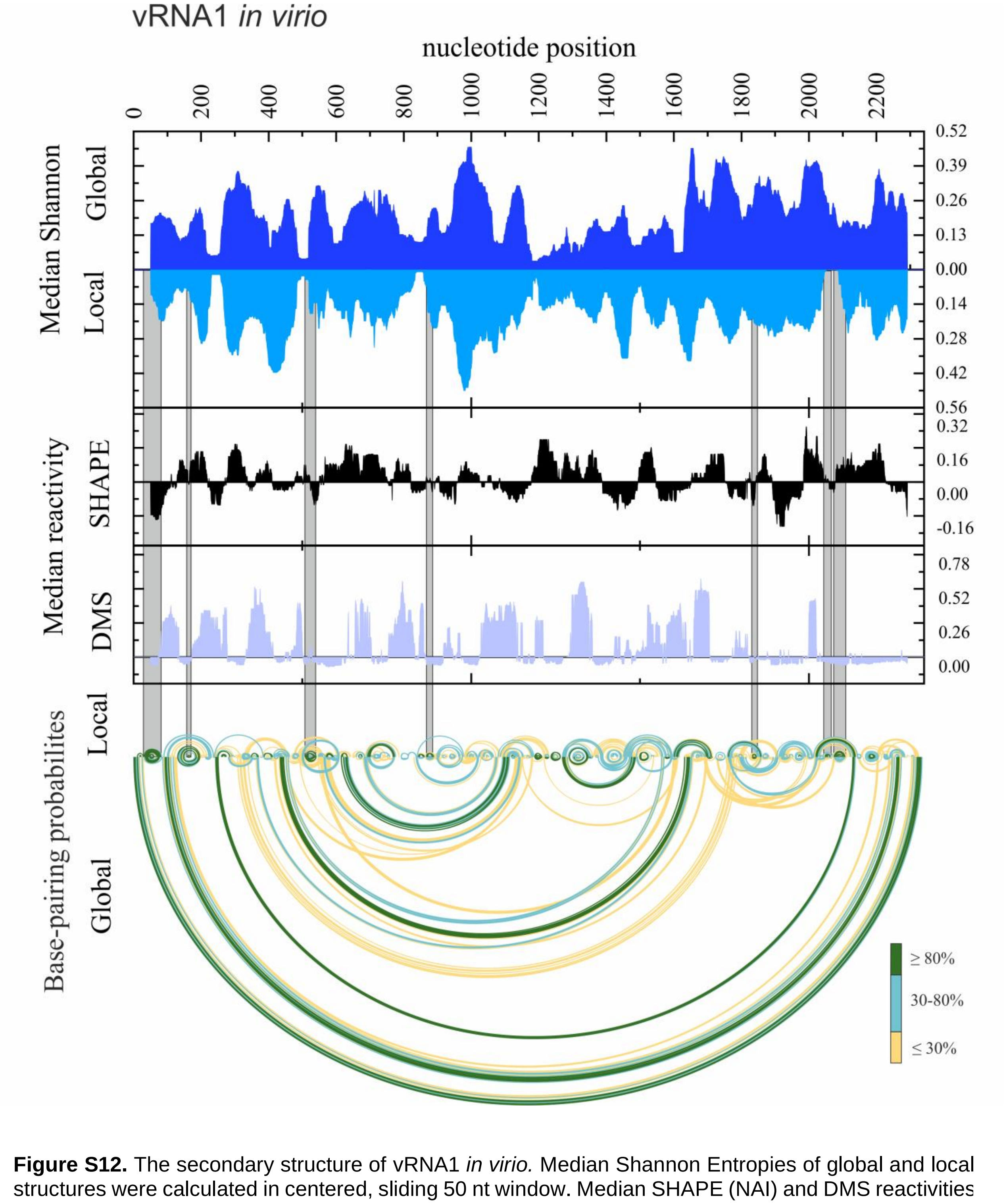

Figure S12. The secondary structure of vRNA1 *in virio.* Median Shannon Entropies of global and local structures were calculated in centered, sliding 50 nt window. Median SHAPE (NAI) and DMS reactivities were calculated in 50 nt window and plotted with respect to global median. Arc plots showing the base-pairing probabilities of predicted local (upper) and global (lower) structures were calculated using partition function (RNAstructure).Grey shadings indicate the low Shannon-SHAPE-DMS regions of the most probable one well-defined structural motifs predicted in both – global and local vRNA7 secondary structures. Regions of 50 nt from the beginning and the end of calculation (Median Shannon, SHAPE and DMS) were excluded from visualization.

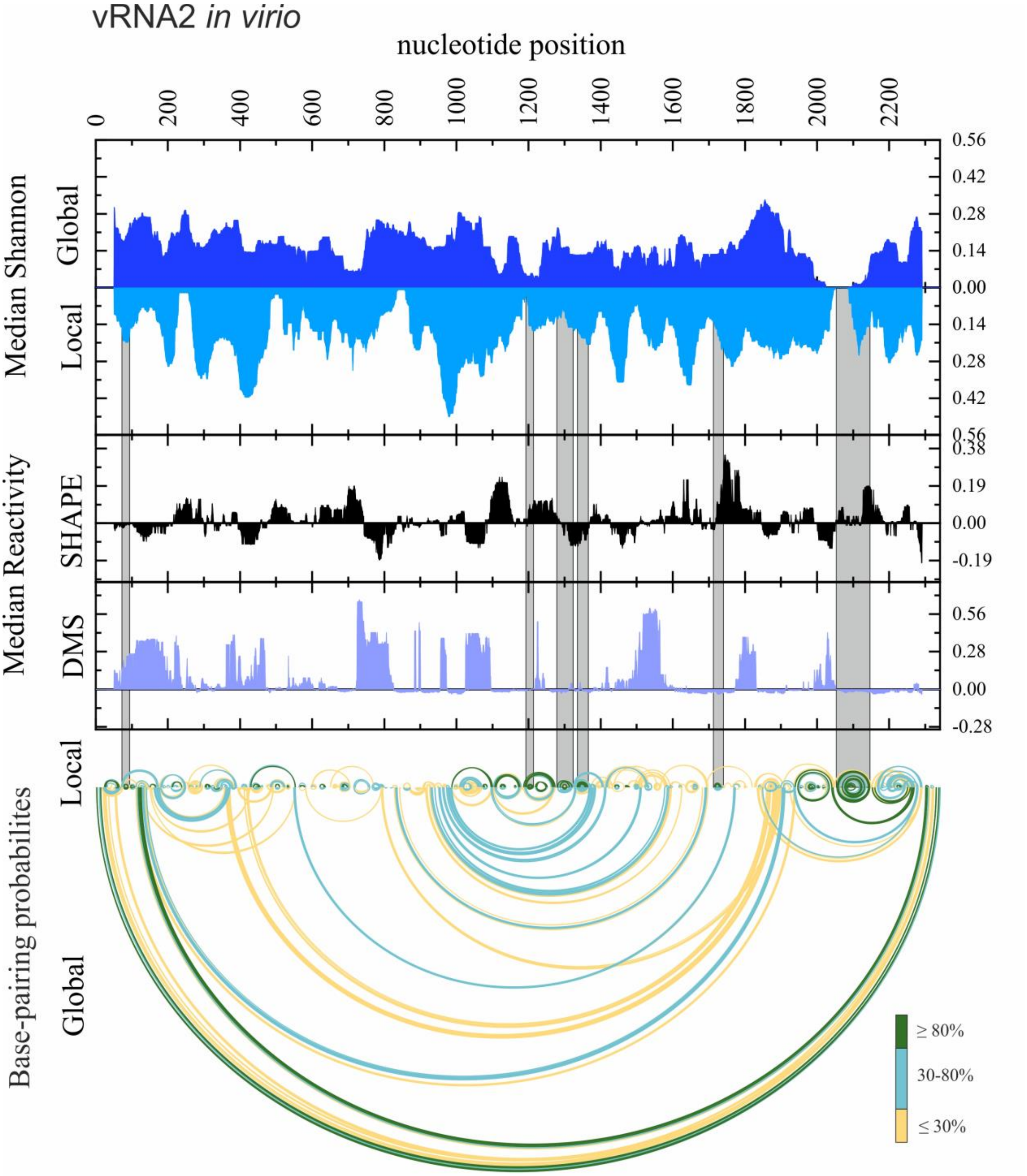

Figure S13. The secondary structure of vRNA2 *in virio.* Median Shannon Entropies of global and local structures were calculated in centered, sliding 50 nt window. Median SHAPE (NAI) and DMS reactivities were calculated in 50 nt window and plotted with respect to global median. Arc plots showing the base-pairing probabilities of predicted local (upper) and global (lower) structures were calculated using partition function (RNAstructure).Grey shadings indicate the low Shannon-SHAPE-DMS regions of the most probable one well-defined structural motifs predicted in both – global and local vRNA7 secondary structures. Regions of 50 nt from the beginning at the end of calculation (Median Shannon, SHAPE and DMS) were excluded from visualization.

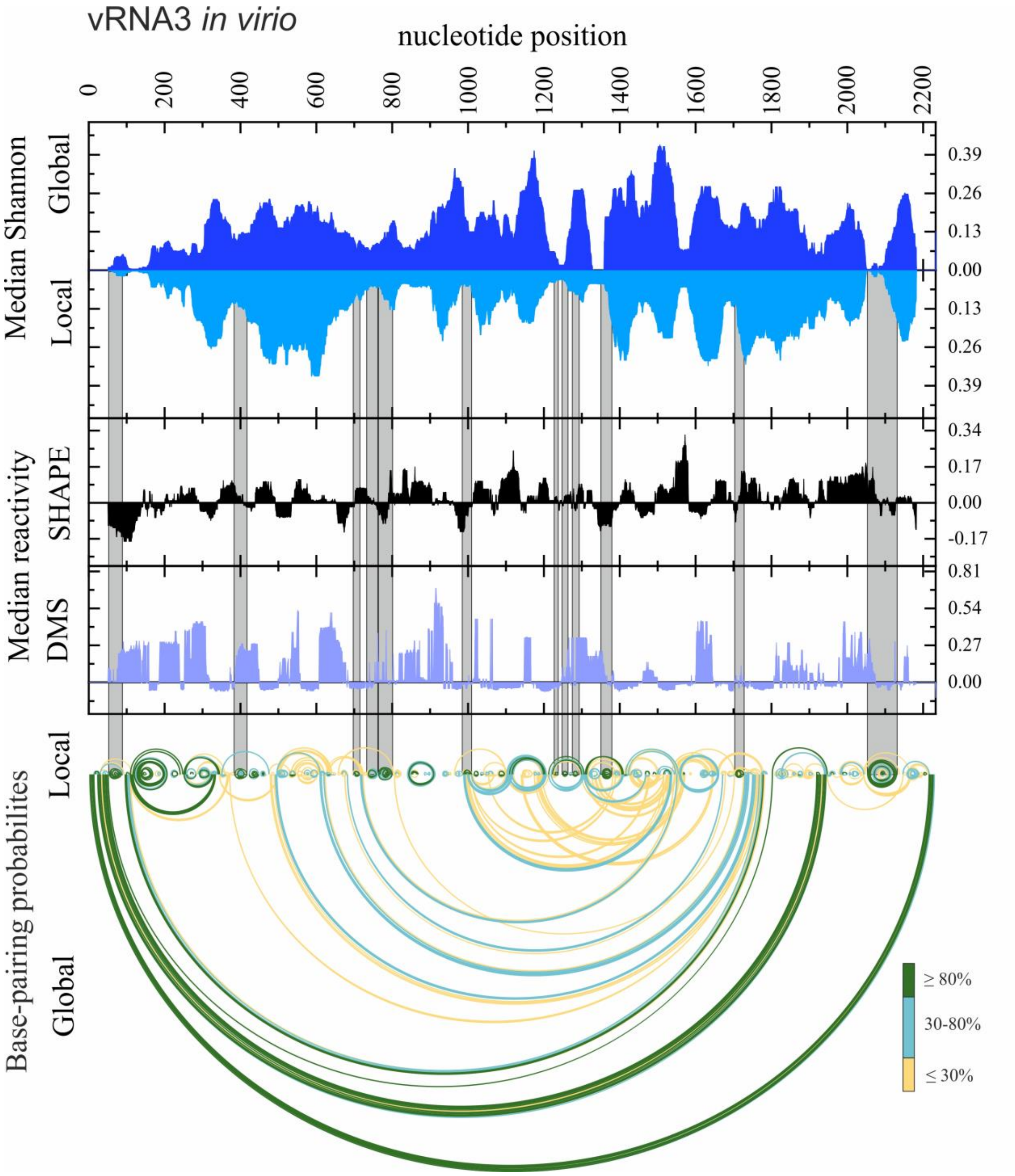

Figure S14. The secondary structure of vRNA2 *in virio.* Median Shannon Entropies of global and local structures were calculated in centered, sliding 50 nt window. Median SHAPE (NAI) and DMS reactivities were calculated in 50 nt window and plotted with respect to global median. Arc plots showing the base-pairing probabilities of predicted local (upper) and global (lower) structures were calculated using partition function (RNAstructure).Grey shadings indicate the low Shannon-SHAPE-DMS regions of the most probable one well-defined structural motifs predicted in both – global and local vRNA7 secondary structures. Regions of 50 nt from the beginning at the end of calculation (Median Shannon, SHAPE and DMS) were excluded from visualization.

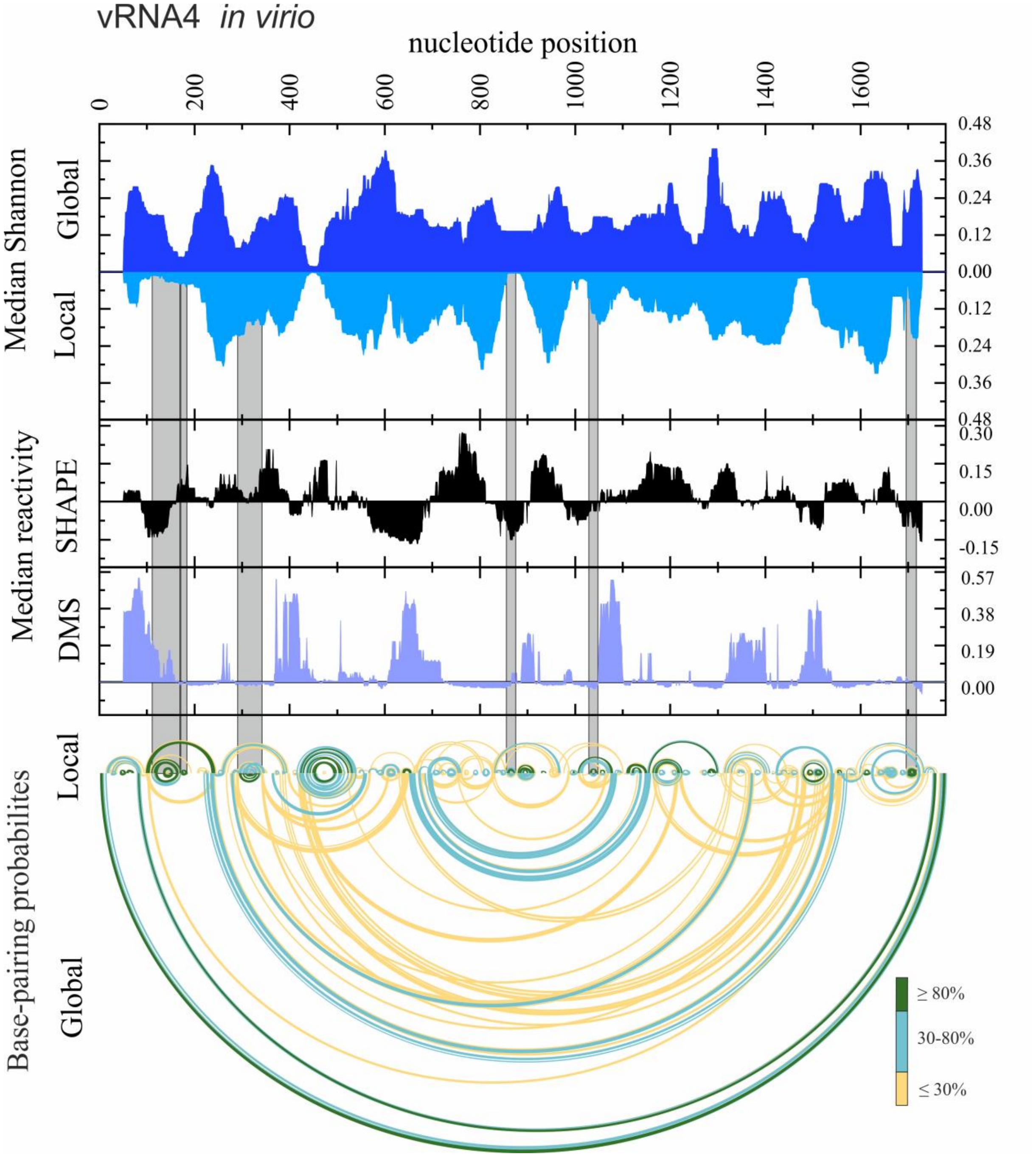

Figure S15. The secondary structure of vRNA4 *in virio.* Median Shannon Entropies of global and local structures were calculated in centered, sliding 50 nt window. Median SHAPE (NAI) and DMS reactivities were calculated in 50 nt window and plotted with respect to global median. Arc plots showing the base-pairing probabilities of predicted local (upper) and global (lower) structures were calculated using partition function (RNAstructure).Grey shadings indicate the low Shannon-SHAPE-DMS regions of the most probable one well-defined structural motifs predicted in both – global and local vRNA7 secondary structures. Regions of 50 nt from the beginning at the end of calculation (Median Shannon, SHAPE and DMS) were excluded from visualization.

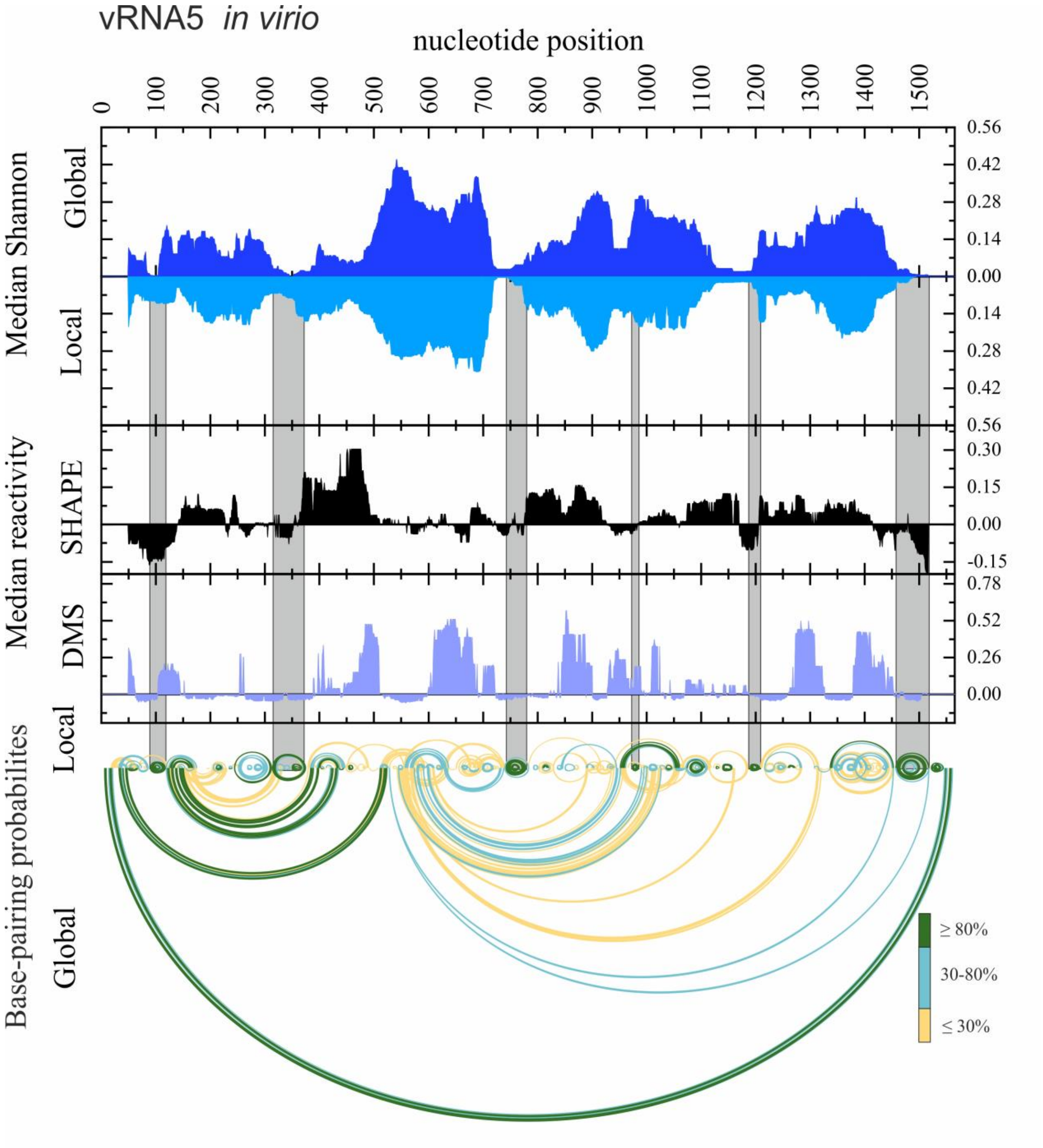

Figure S16. The secondary structure of vRNA5 *in virio.* Median Shannon Entropies of global and local structures were calculated in centered, sliding 50 nt window. Median SHAPE (NAI) and DMS reactivities were calculated in 50 nt window and plotted with respect to global median. Arc plots showing the base-pairing probabilities of predicted local (upper) and global (lower) structures were calculated using partition function (RNAstructure).Grey shadings indicate the low Shannon-SHAPE-DMS regions of the most probable one well-defined structural motifs predicted in both – global and local vRNA7 secondary structures. Regions of 50 nt from the beginning at the end of calculation (Median Shannon, SHAPE and DMaaS) were excluded from visualization.

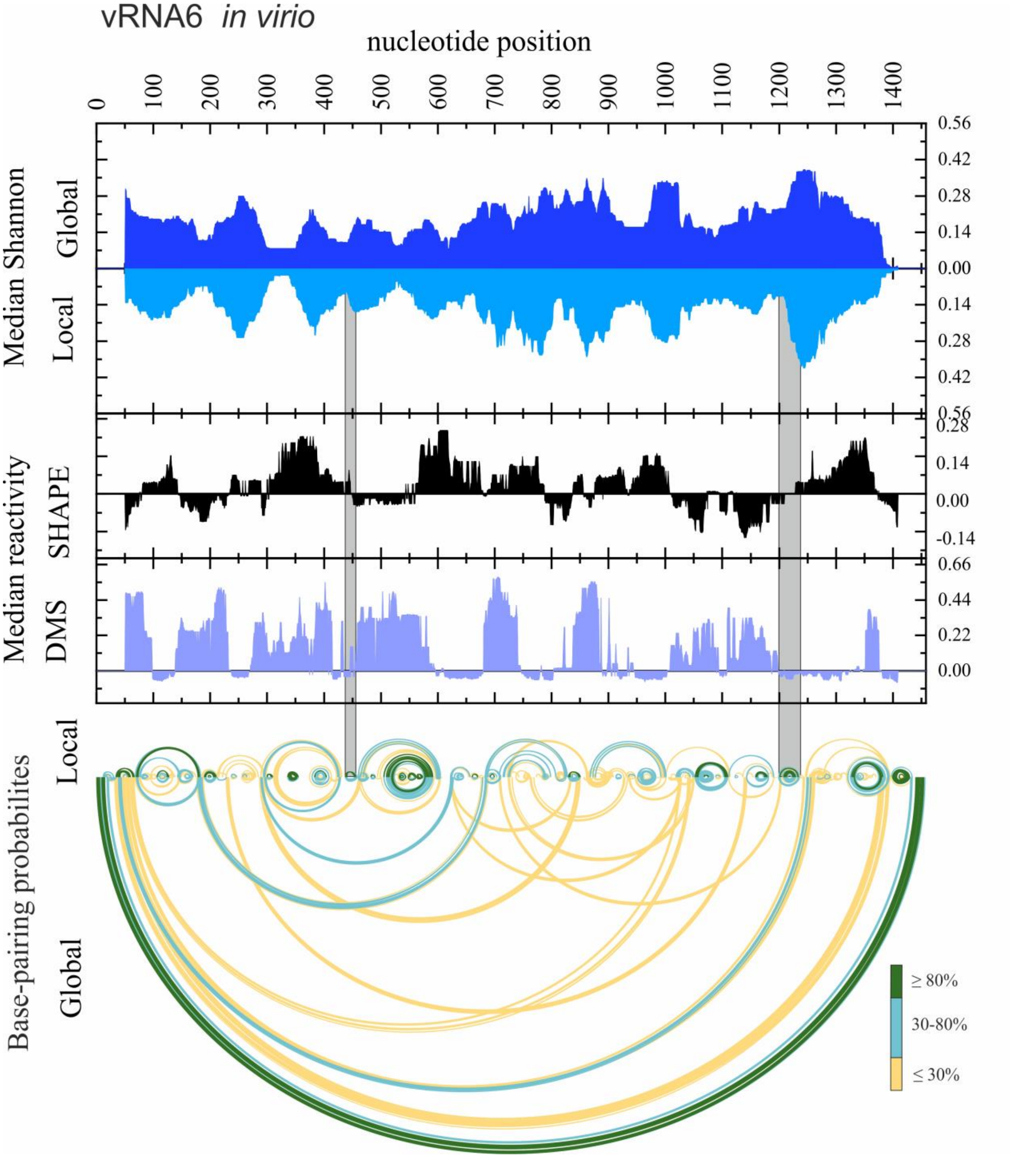

Figure S17. The secondary structure of vRNA6 *in virio.* Median Shannon Entropies of global and local structures were calculated in centered, sliding 50 nt window. Median SHAPE (NAI) and DMS reactivities were calculated in 50 nt window and plotted with respect to global median. Arc plots showing the base-pairing probabilities of predicted local (upper) and global (lower) structures were calculated using partition function (RNAstructure).Grey shadings indicate the low Shannon-SHAPE-DMS regions of the most probable one well-defined structural motifs predicted in both – global and local vRNA7 secondary structures. Regions of 50 nt from the beginning at the end of calculation (Median Shannon, SHAPE and DMS) were excluded from visualization.

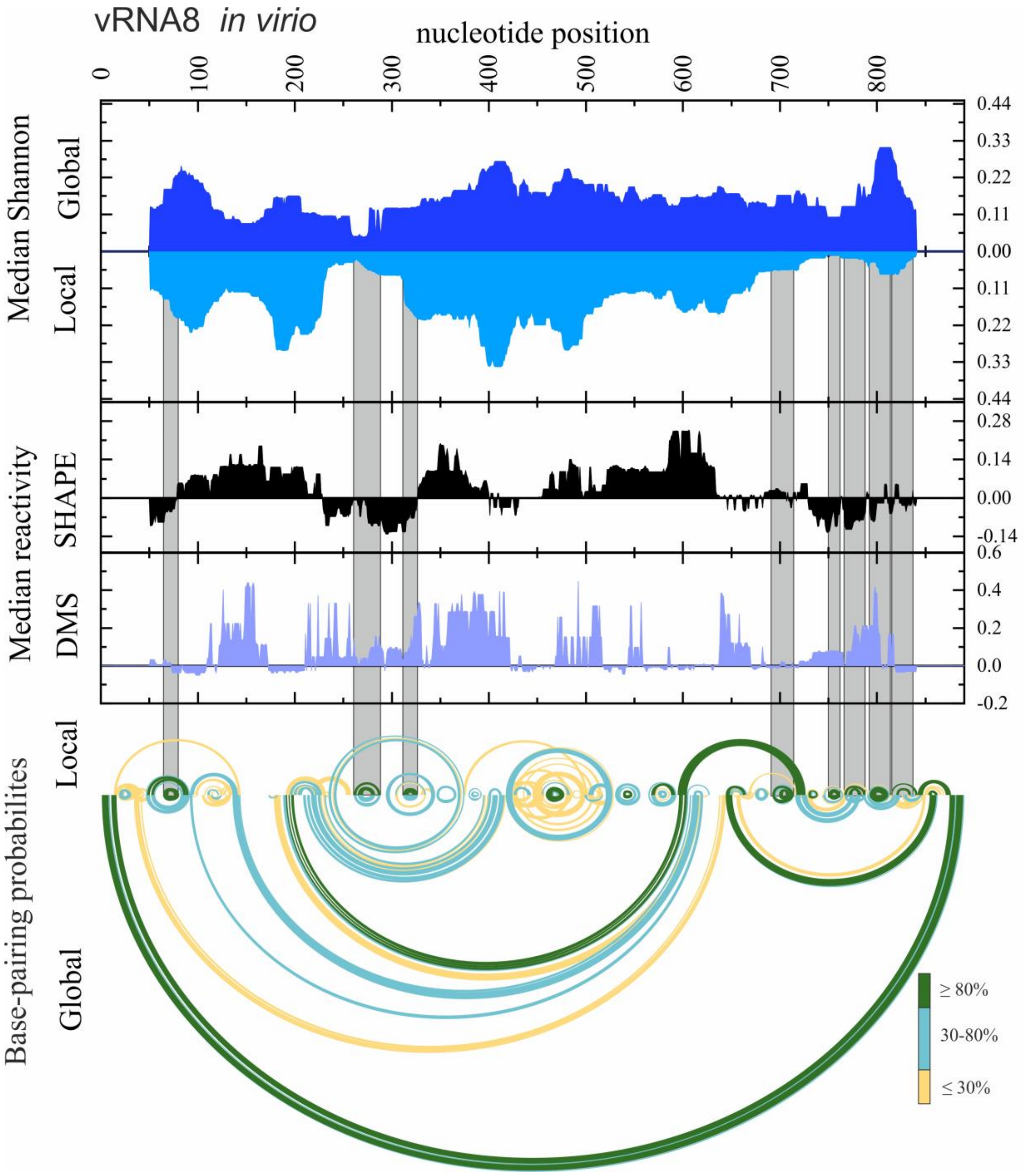

Figure S18. The secondary structure of vRNA8 *in virio.* Median Shannon Entropies of global and local structures were calculated in centered, sliding 50 nt window. Median SHAPE (NAI) and DMS reactivities were calculated in 50 nt window and plotted with respect to global median. Arc plots showing the base-pairing probabilities of predicted local (upper) and global (lower) structures were calculated using partition function (RNAstructure).Grey shadings indicate the low Shannon-SHAPE-DMS regions of the most probable one well-defined structural motifs predicted in both – global and local vRNA7 secondary structures. Regions of 50 nt from the beginning at the end of calculation (Median Shannon, SHAPE and DMS) were excluded from visualization.

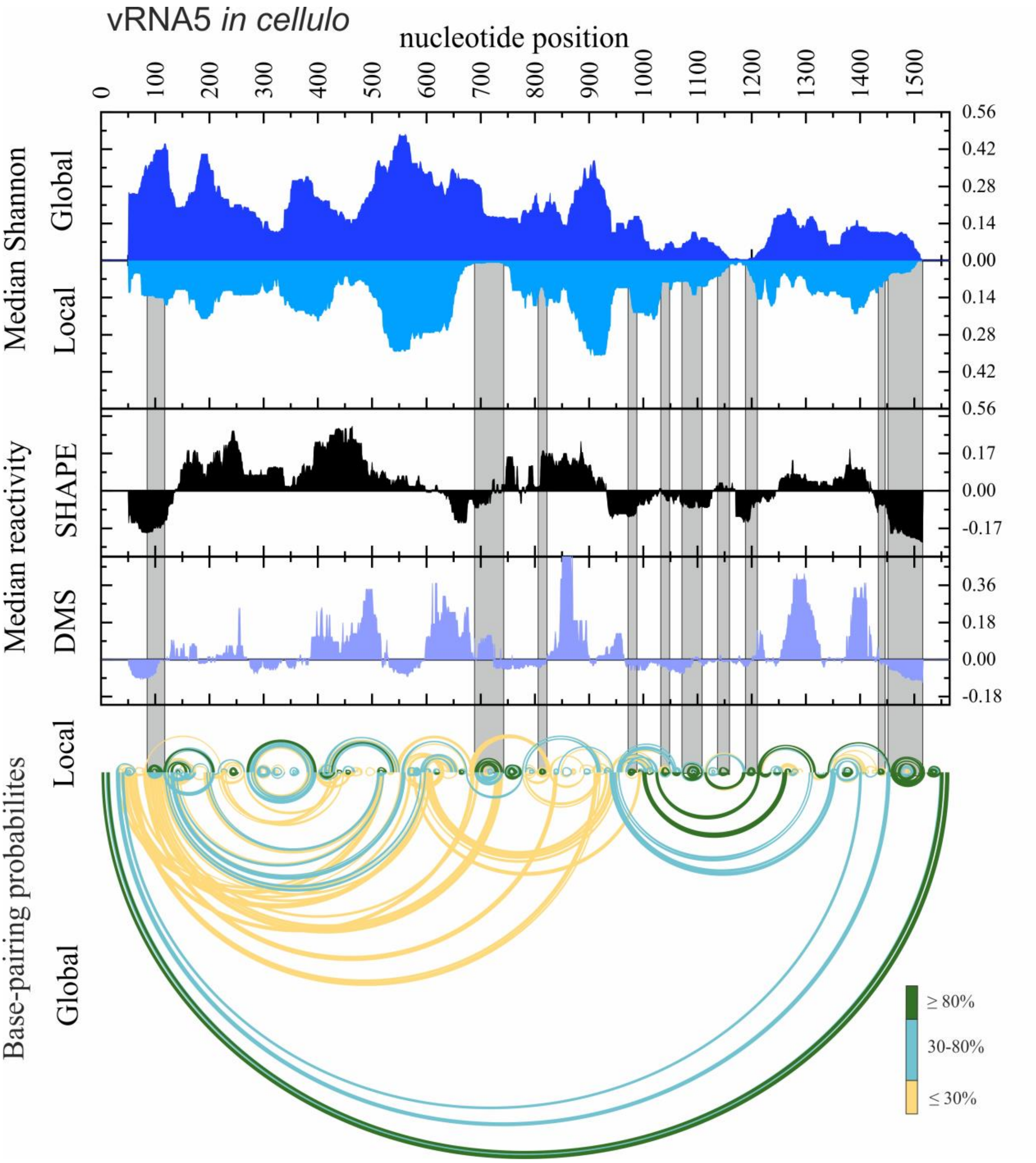

Figure S19. The secondary structure of vRNA5 *in cellulo.* Median Shannon Entropies of global and local structures were calculated in centered, sliding 50 nt window. Median SHAPE (NAI) and DMS reactivities were calculated in 50 nt window and plotted with respect to global median. Arc plots showing the base-pairing probabilities of predicted local (upper) and global (lower) structures were calculated using partition function (RNAstructure).Grey shadings indicate the low Shannon-SHAPE-DMS regions of the most probable one well-defined structural motifs predicted in both – global and local vRNA7 secondary structures. Regions of 50 nt from the beginning at the end of calculation (Median Shannon, SHAPE and DMS) were excluded from visualization.

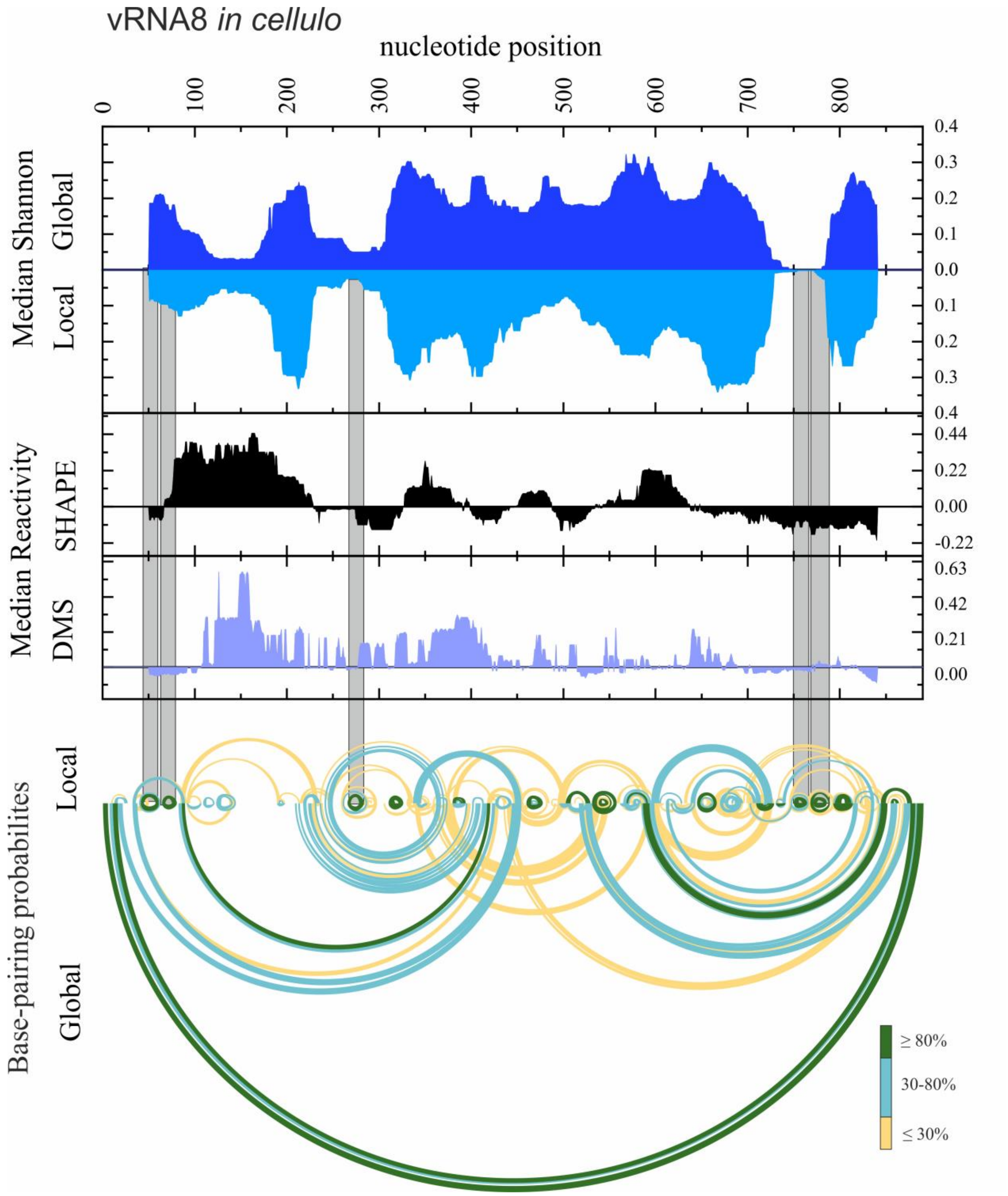

Figure S20. The secondary structure of vRNA8 *in cellulo.* Median Shannon Entropies of global and local structures were calculated in centered, sliding 50 nt window. Median SHAPE (NAI) and DMS reactivities were calculated in 50 nt window and plotted with respect to global median. Arc plots showing the base-pairing probabilities of predicted local (upper) and global (lower) structures were calculated using partition function (RNAstructure).Grey shadings indicate the low Shannon-SHAPE-DMS regions of the most probable one well-defined structural motifs predicted in both – global and local vRNA7 secondary structures. Regions of 50 nt from the beginning at the end of calculation (Median Shannon, SHAPE and DMS) were excluded from visualization.
