# Supplementary material for: In vivo secondary structural analysis of Influenza A virus genomic RNA
Source: Cell Mol Life Sci. 2023 May 2;80(5):136. doi: 10.1007/s00018-023-04764-1 (PMC10153785; doi:10.1007/s00018-023-04764-1)
Supplement: Supplementary file 9 — Supplementary file9 (PDF 17363 KB) [file 18_2023_4764_MOESM9_ESM.pdf]

## Supplementary F2

# *In vivo* secondary structural analysis of Influenza A virus genomic RNA

Barbara Mirska[1], Tomasz Woźniak[2], Dagny Lorent[1], Agnieszka Ruszkowska[1], Jake M. Peterson[3], Walter N. Moss[3], David H. Mathews[4], Ryszard Kierzek[1] and Elzbieta Kierzek[1*]

[1] Institute of Bioorganic Chemistry, Polish Academy of Sciences, Noskowskiego 12/14, 61-704 Poznan, Poland

[2] Institute of Human Genetics, Polish Academy of Sciences, Strzeszynska 32, 60-479 Poznan, Poland

[3] Roy J. Carver Department of Biophysics, Biochemistry and Molecular Biology, Iowa State University, Ames, IA 50011, USA

[4] Department of Biochemistry & Biophysics and Center for RNA Biology, 601 Elmwood Avenue, Box 712, School of Medicine and Dentistry, University of Rochester, Rochester, NY 14642, USA

*elzbieta.kierzek@ibch.poznan.pl

**Supplementary F2** material contains the influenza A virus (A/California/04/2009) vRNA secondary structures predictions from *in virio* and *in cellulo* experiments.

The structures were predicted using the same experimental data originating from chemical probing experiments with NAI (SHAPE reagent) and DMS, but with different folding algorithms. Minimum Free Energy (MFE structure) as well as Maximum Expected Accuracy (MEA structure) were calculated using RNAstructure software for vRNAs. Additionally, the structures obtained using aforementioned algorithms were predicted with 150 nt (local structure) and without (global structure) maximum allowed base-pairing distance constraints.

The information concerning base-pairing conservation across type A influenza is indicated on each structure using color-based code. The colors indicate percentage range of canonical base pairing preserved among IAV sequences of presented vRNA and are described in each figure legend. The conservation values of each predicted structure are included in Supplementary E2-E5 files.

The vRNA structures are presented in the following order:

*In virio* vRNA structures

1. vRNA1 *in virio* MEA global structure
2. vRNA1 *in virio* MFE global structure
3. vRNA1 *in virio* MEA local structure
4. vRNA1 *in virio* MFE local structure
5. vRNA2 *in virio* MEA global structure
6. vRNA2 *in virio* MFE global structure
7. vRNA2 *in virio* MEA local structure
8. vRNA2 *in virio* MFE local structure
9. vRNA3 *in virio* MEA global structure
10. vRNA3 *in virio* MFE global structure
11. vRNA3 *in virio* MEA local structure
12. vRNA3 *in virio* MFE local structure
13. vRNA4 *in virio* MEA global structure
14. vRNA4 *in virio* MFE global structure
15. vRNA4 *in virio* MEA local structure
16. vRNA4 *in virio* MFE local structure
17. vRNA5 *in virio* MEA global structure
18. vRNA5 *in virio* MFE global structure
19. vRNA5 *in virio* MEA local structure
20. vRNA5 *in virio* MFE local structure
21. vRNA6 *in virio* MEA global structure
22. vRNA6 *in virio* MFE global structure
23. vRNA6 *in virio* MEA local structure
24. vRNA6 *in virio* MFE local structure
25. vRNA7 *in virio* MEA global structure
26. vRNA7 *in virio* MFE global structure
27. vRNA7 *in virio* MEA local structure
28. vRNA7 *in virio* MFE local structure
29. vRNA8 *in virio* MEA global structure
30. vRNA8 *in virio* MFE global structure
31. vRNA8 *in virio* MEA local structure
32. vRNA8 *in virio* MFE local structure

*In cellulo* vRNA structures

33. vRNA5 *in cellulo* global MEA structure
34. vRNA5 *in cellulo* global MFE structure
35. vRNA5 *in cellulo* local MEA structure
36. vRNA5 *in cellulo* local MFE structure
37. vRNA7 *in cellulo* global MEA structure
38. vRNA7 *in cellulo* global MFE structure
39. vRNA7 *in cellulo* local MEA structure
40. vRNA7 *in cellulo* local MFE structure
41. vRNA8 *in cellulo* global MEA structure
42. vRNA8 *in cellulo* global MFE structure
43. vRNA8 *in cellulo* local MEA structure
44. vRNA8 *in cellulo* local MFE structure

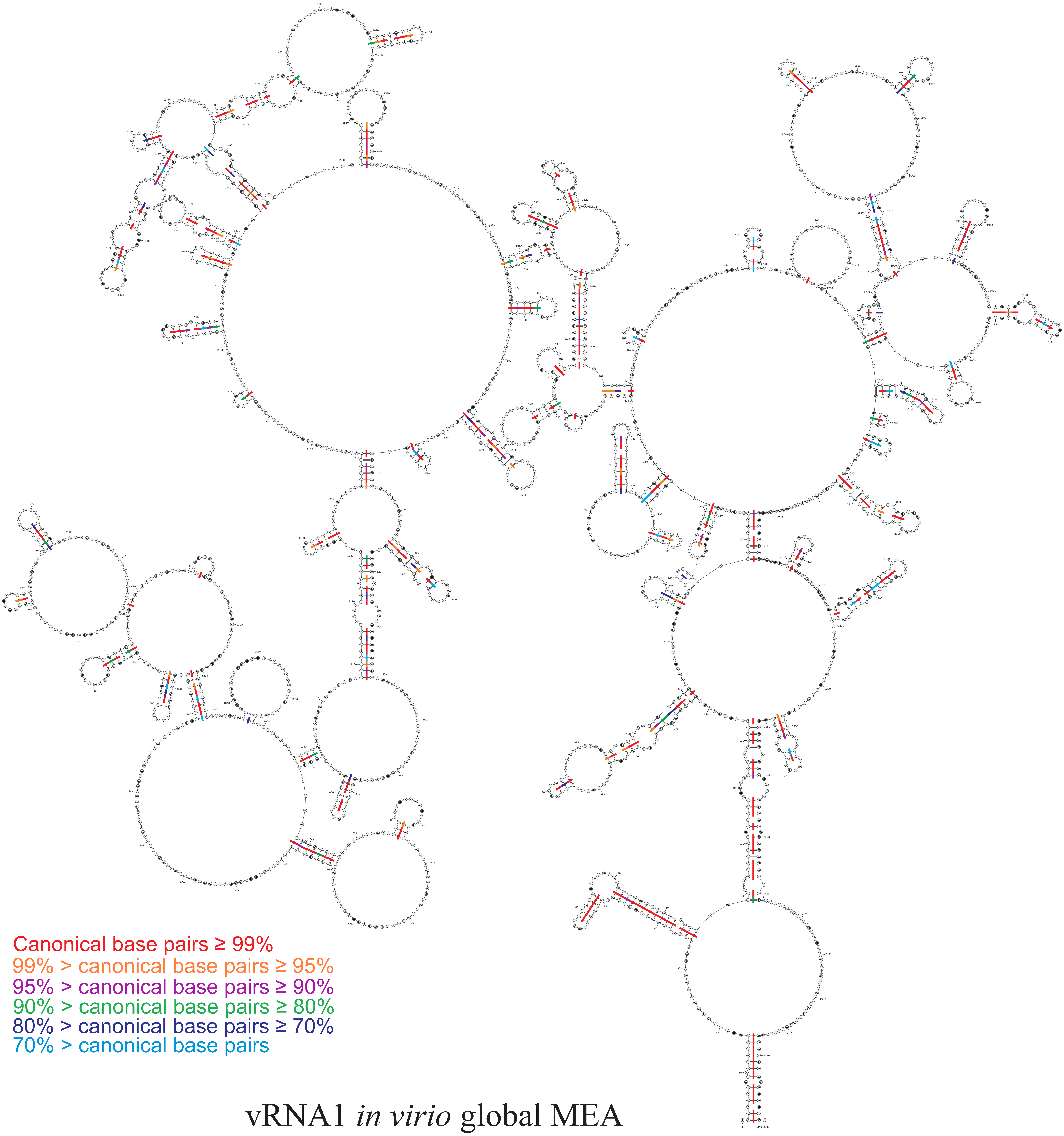


vRNA1 *in virio* global MEA

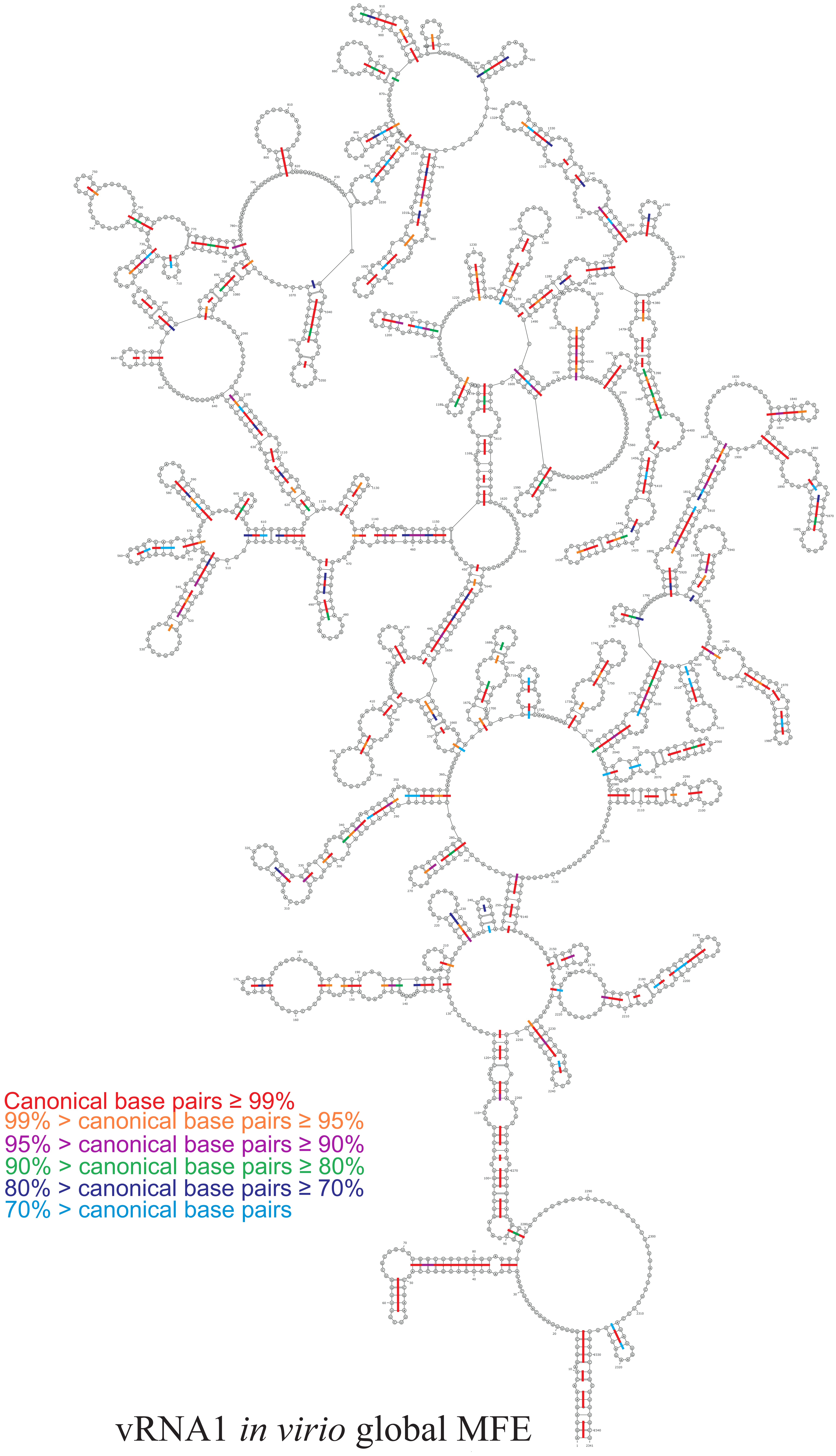


vRNA1 *in virio* global MFE

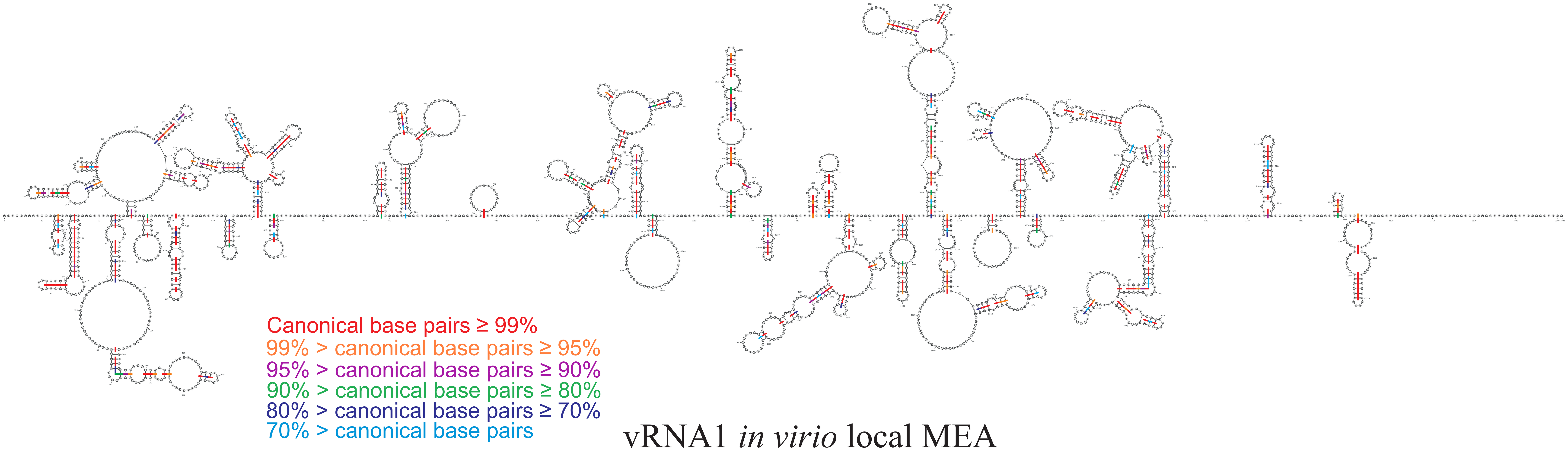


vRNA1 *in virio* local MEA

Canonical base pairs ≥ 99%
99% > canonical base pairs ≥ 95%
95% > canonical base pairs ≥ 90%
90% > canonical base pairs ≥ 80%
80% > canonical base pairs ≥ 70%
70% > canonical base pairs

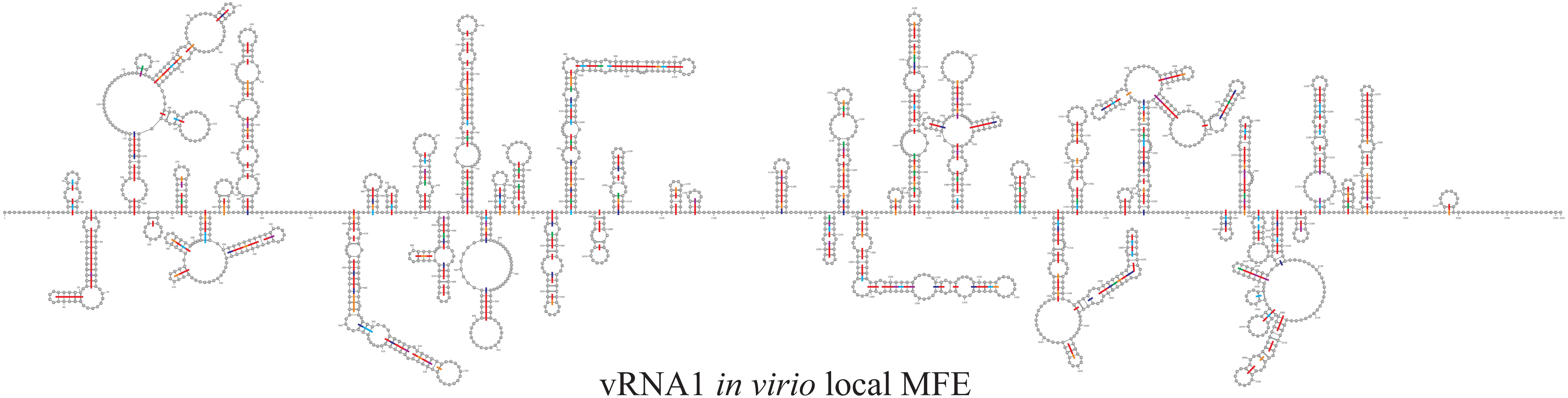

vRNA1 *in virio* local MFE

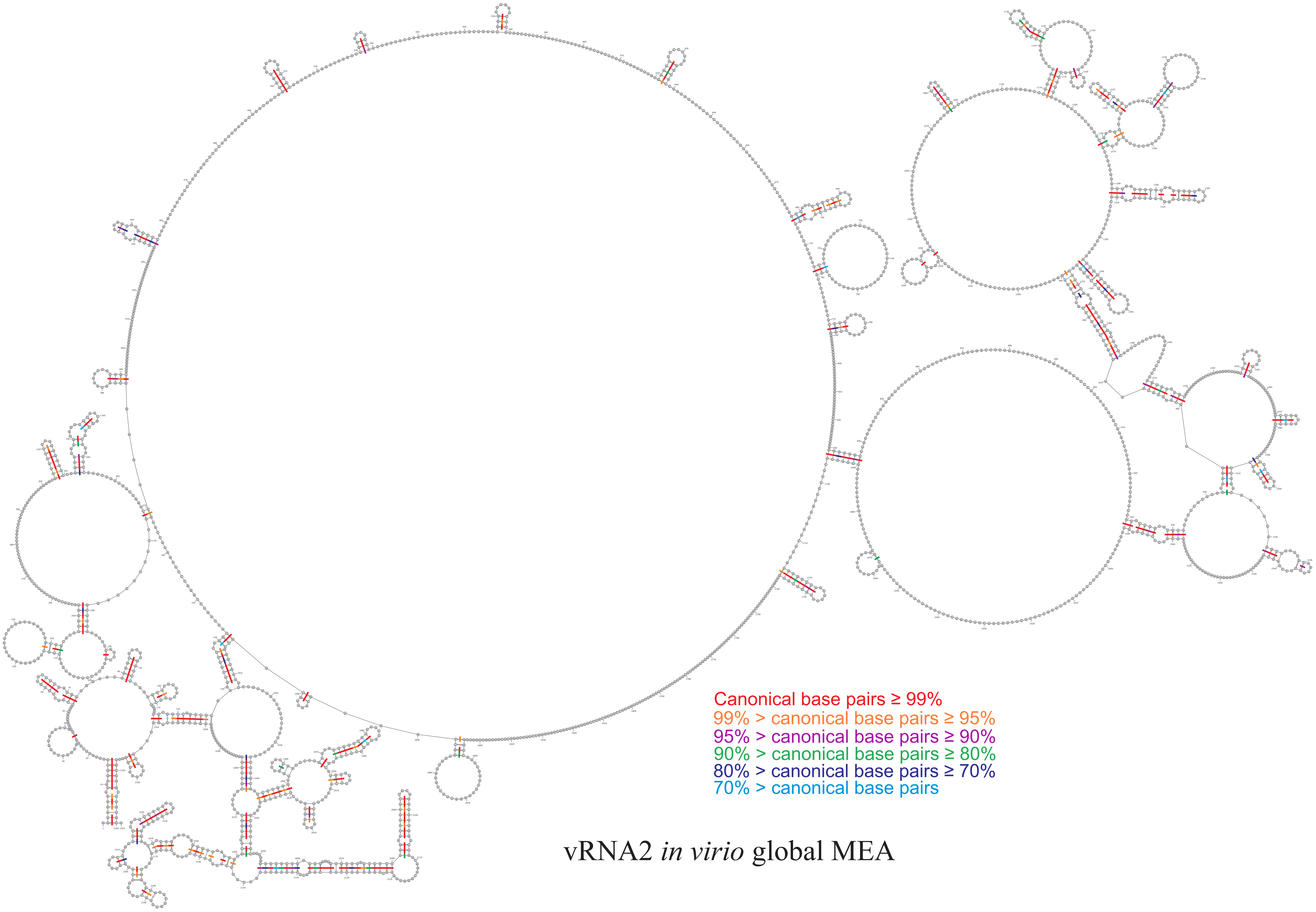


vRNA2 *in virio* global MEA

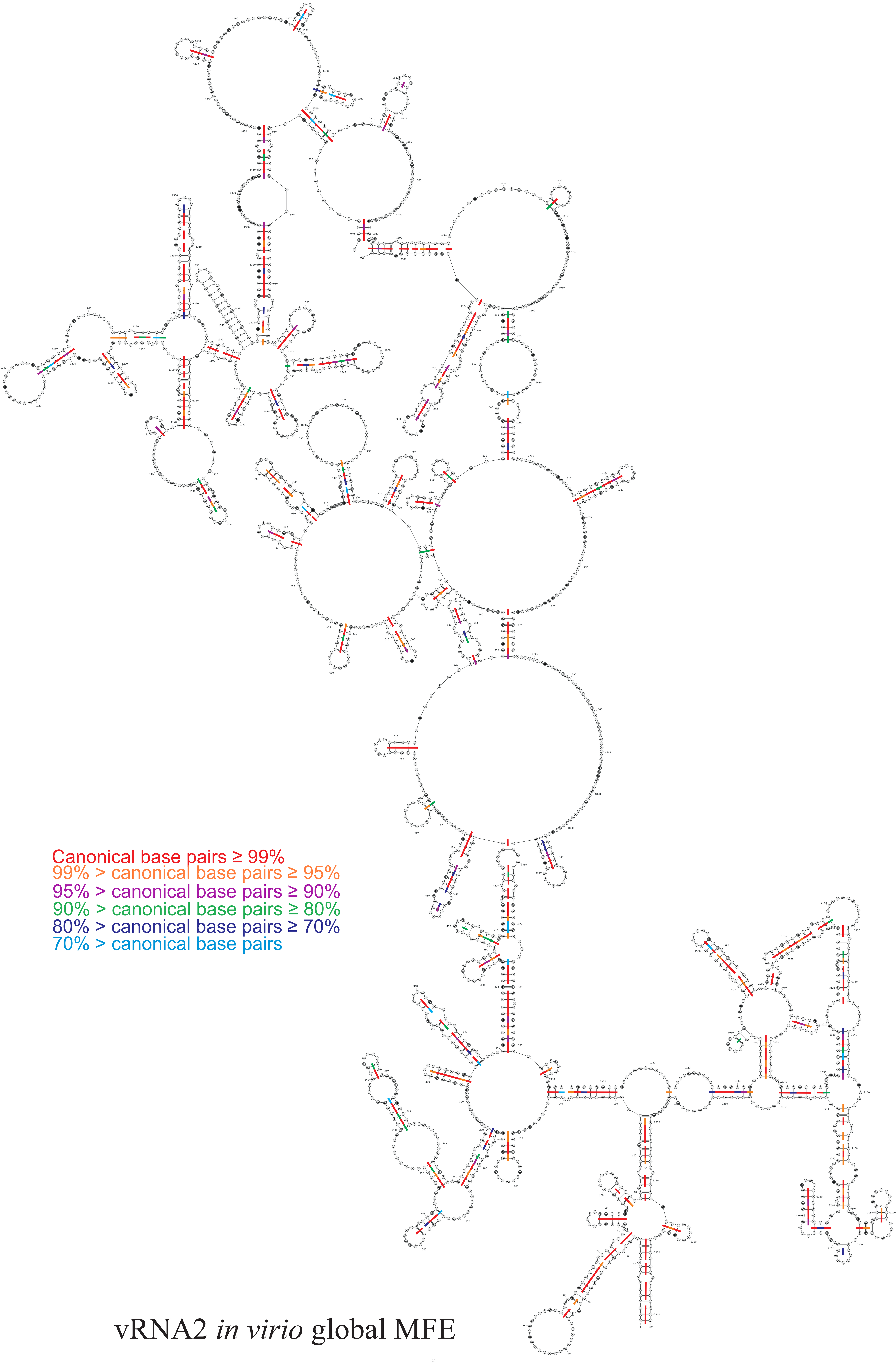


vRNA2 *in virio* global MFE

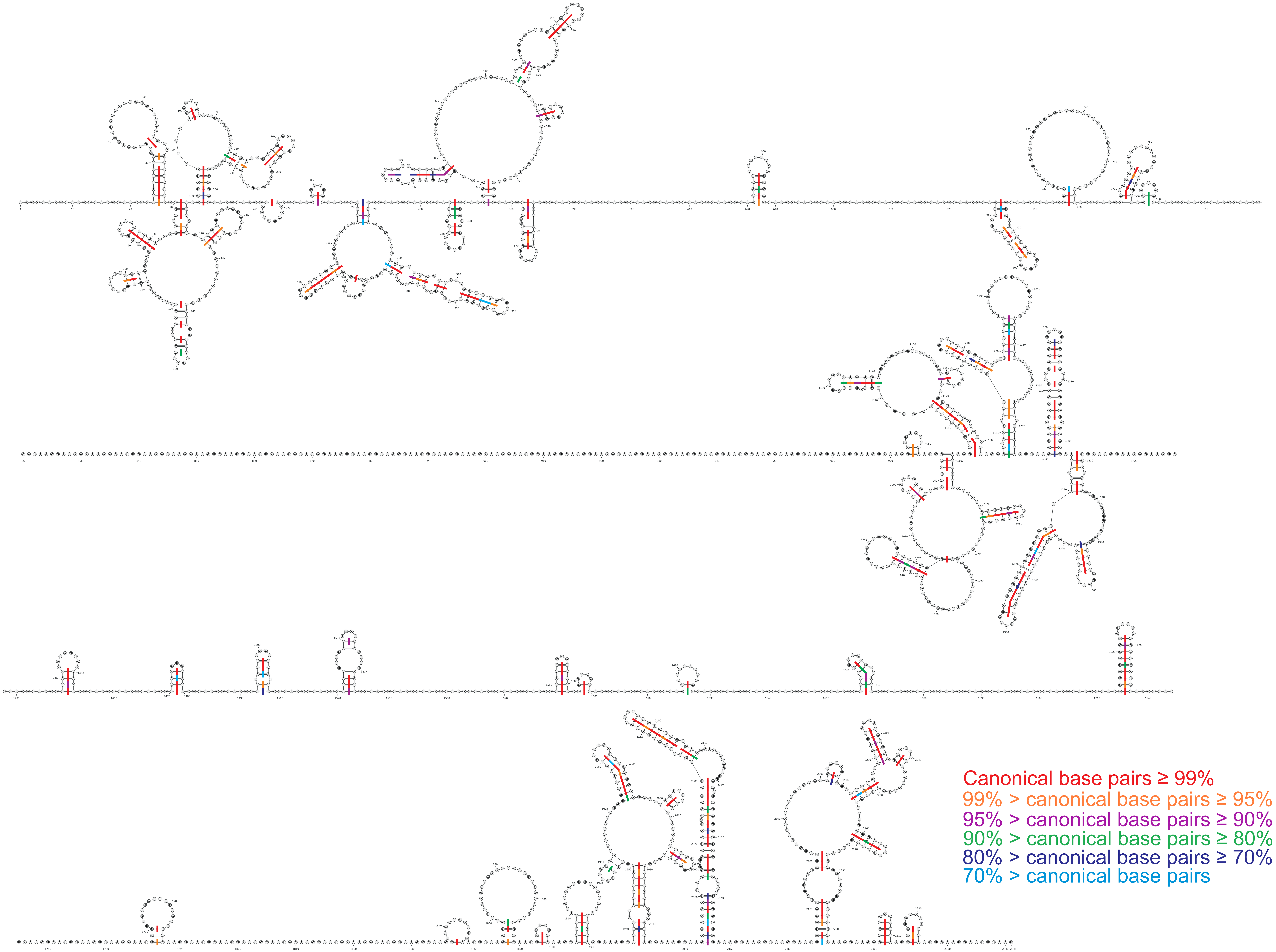


vRNA2 *in virio* local MEA

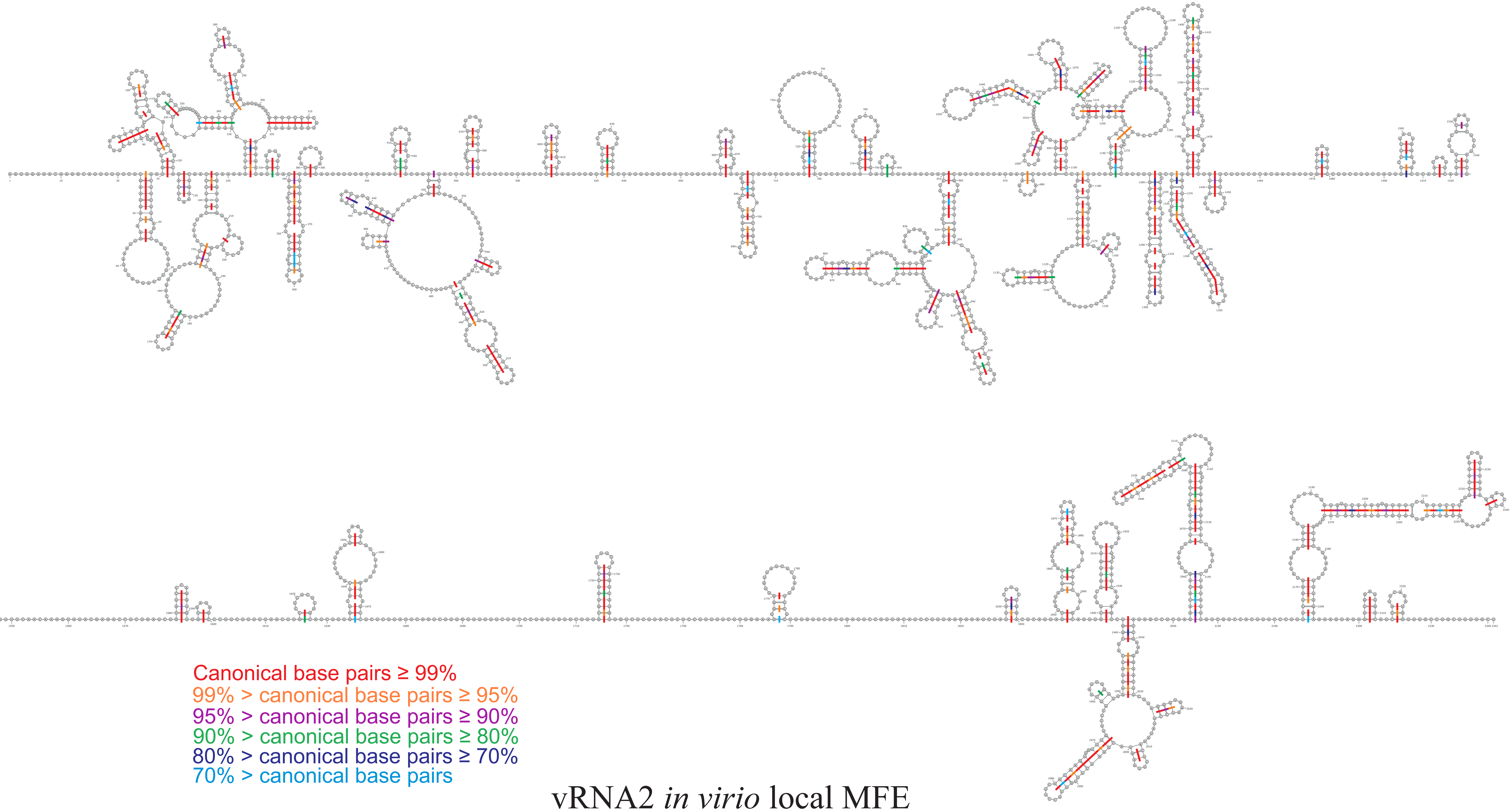


vRNA2 *in virio* local MFE

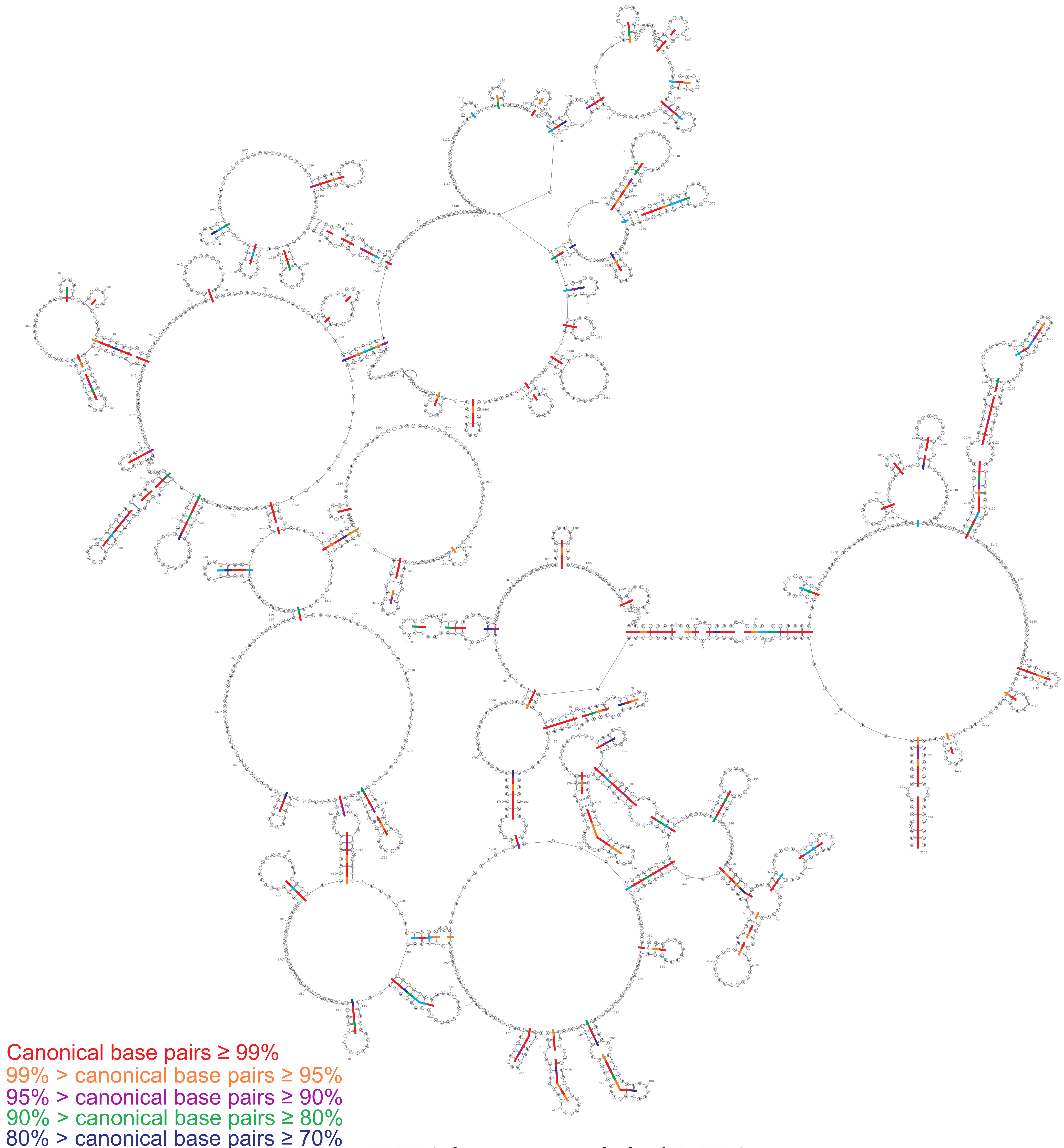


vRNA3 *in virio* global MEA

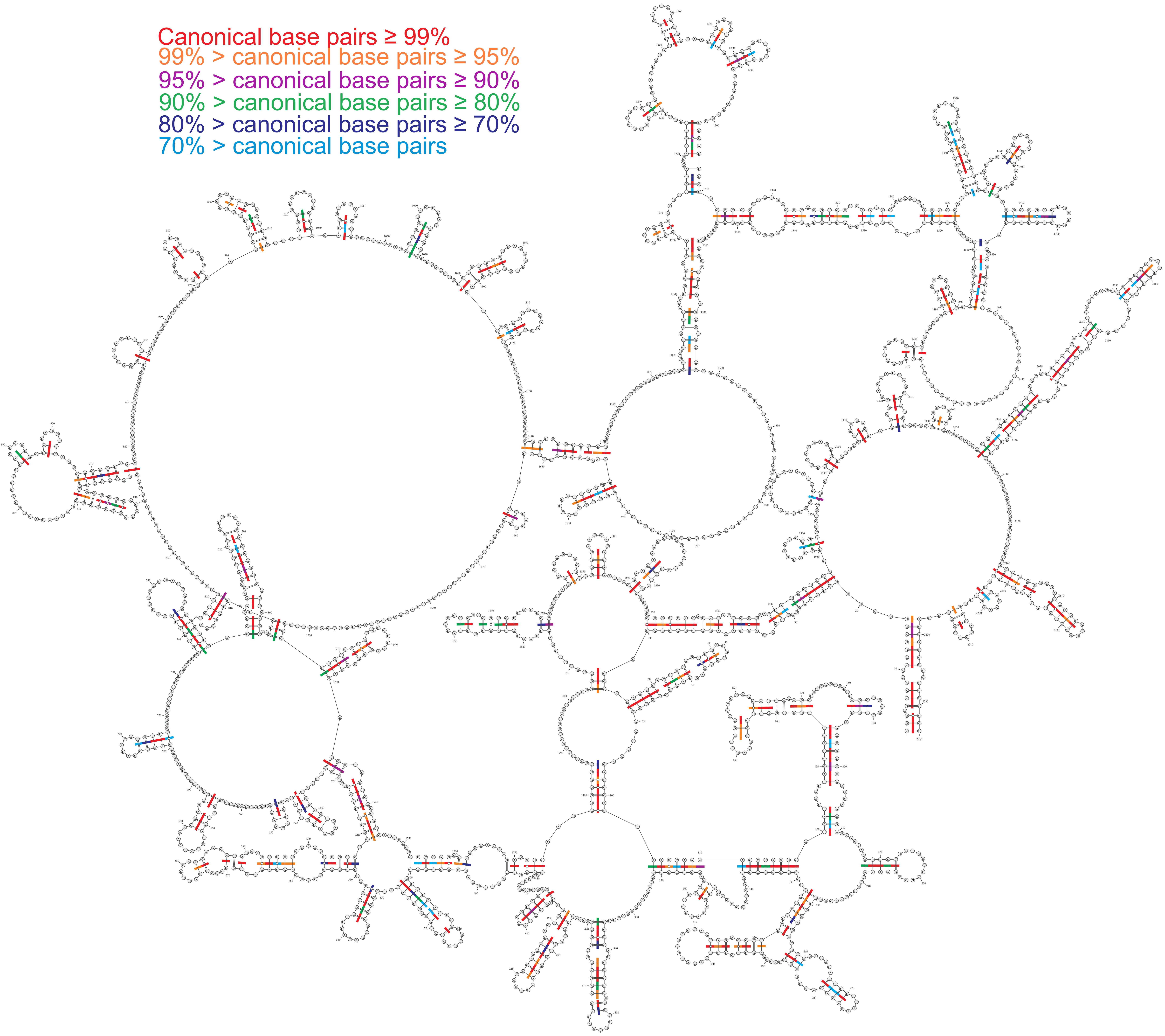


vRNA3 *in virio* global MFE

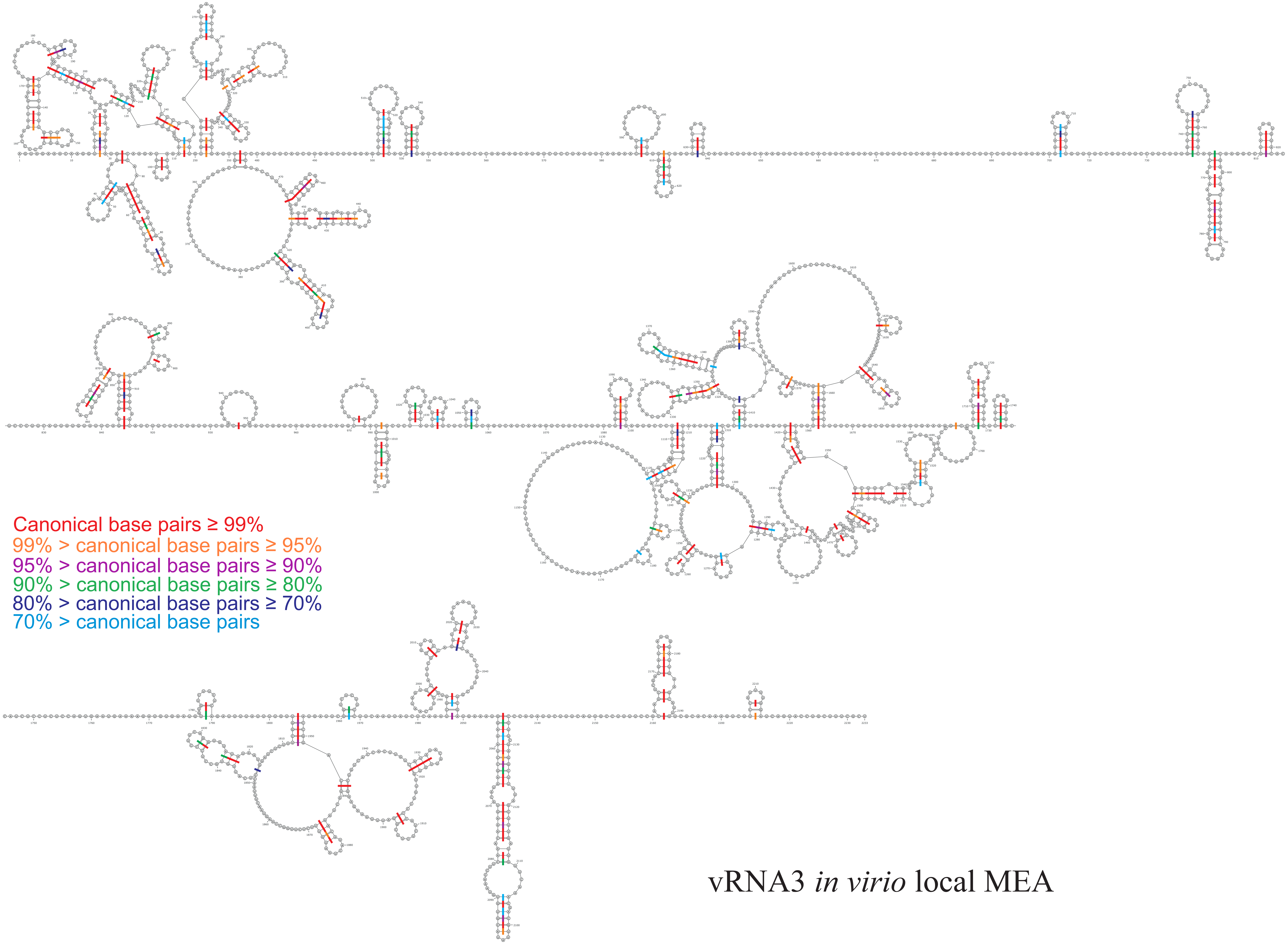


vRNA3 *in virio* local MEA

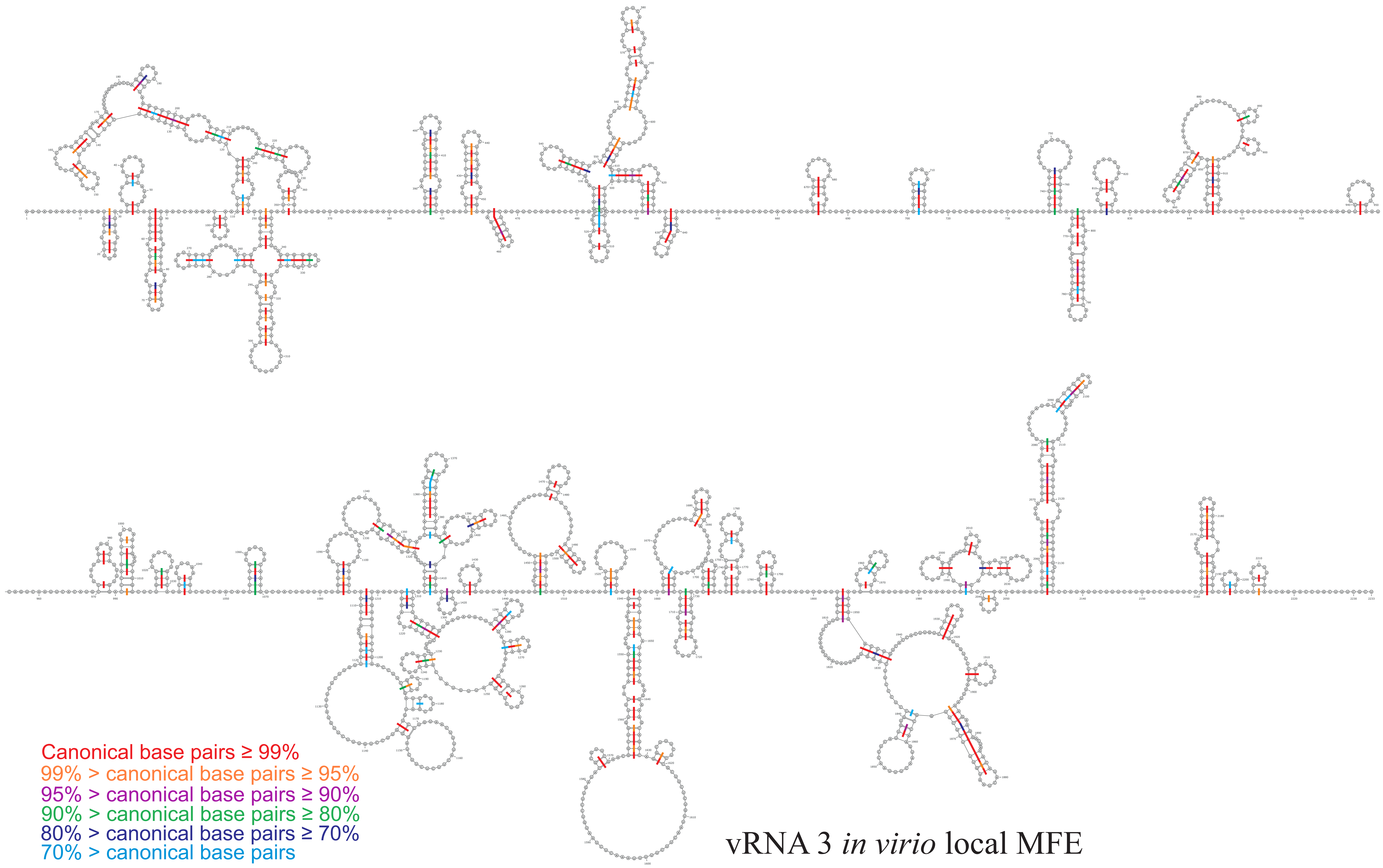


vRNA 3 *in virio* local MFE

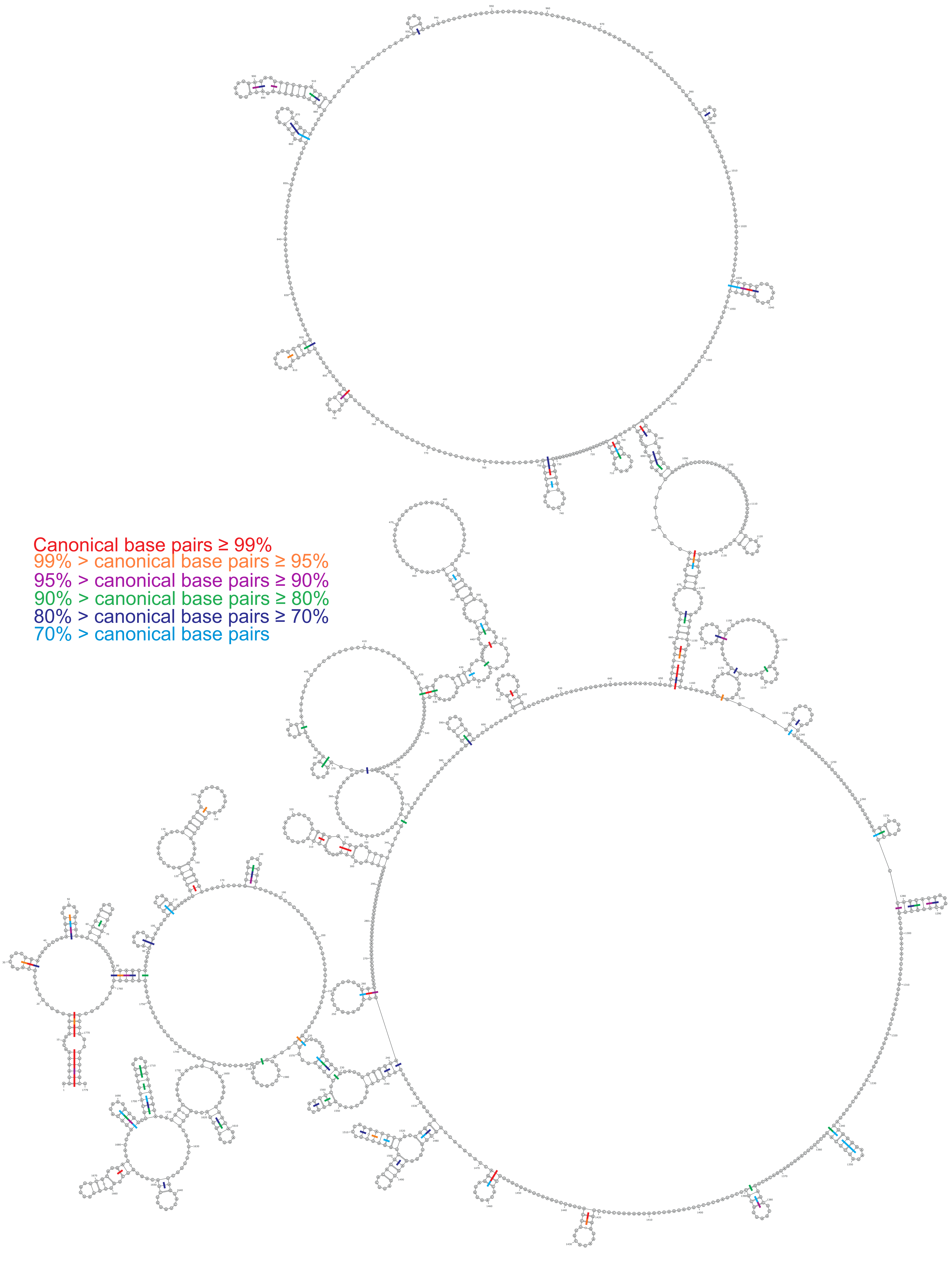


vRNA4 *in virio* global MEA

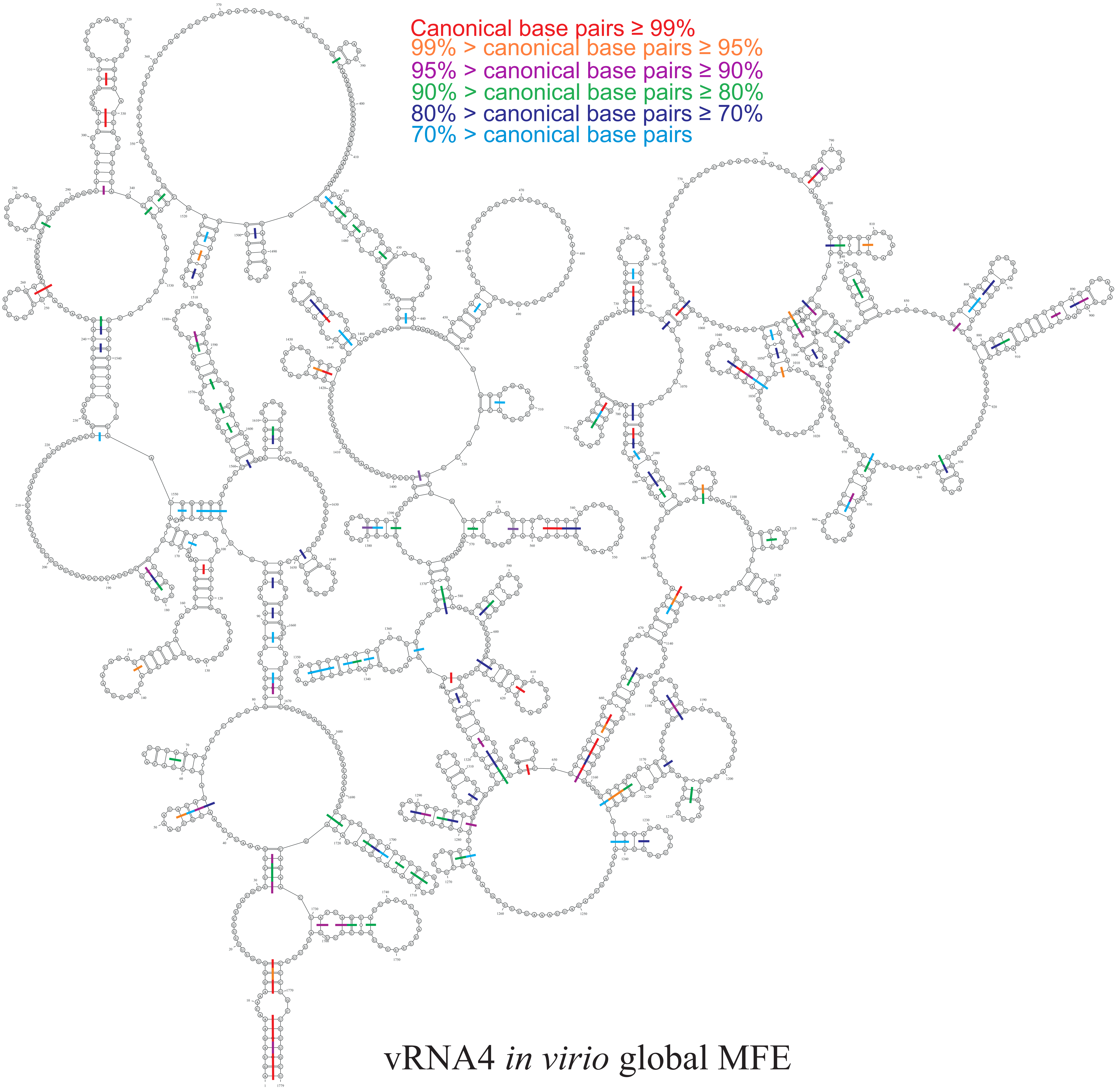


vRNA4 *in virio* global MFE

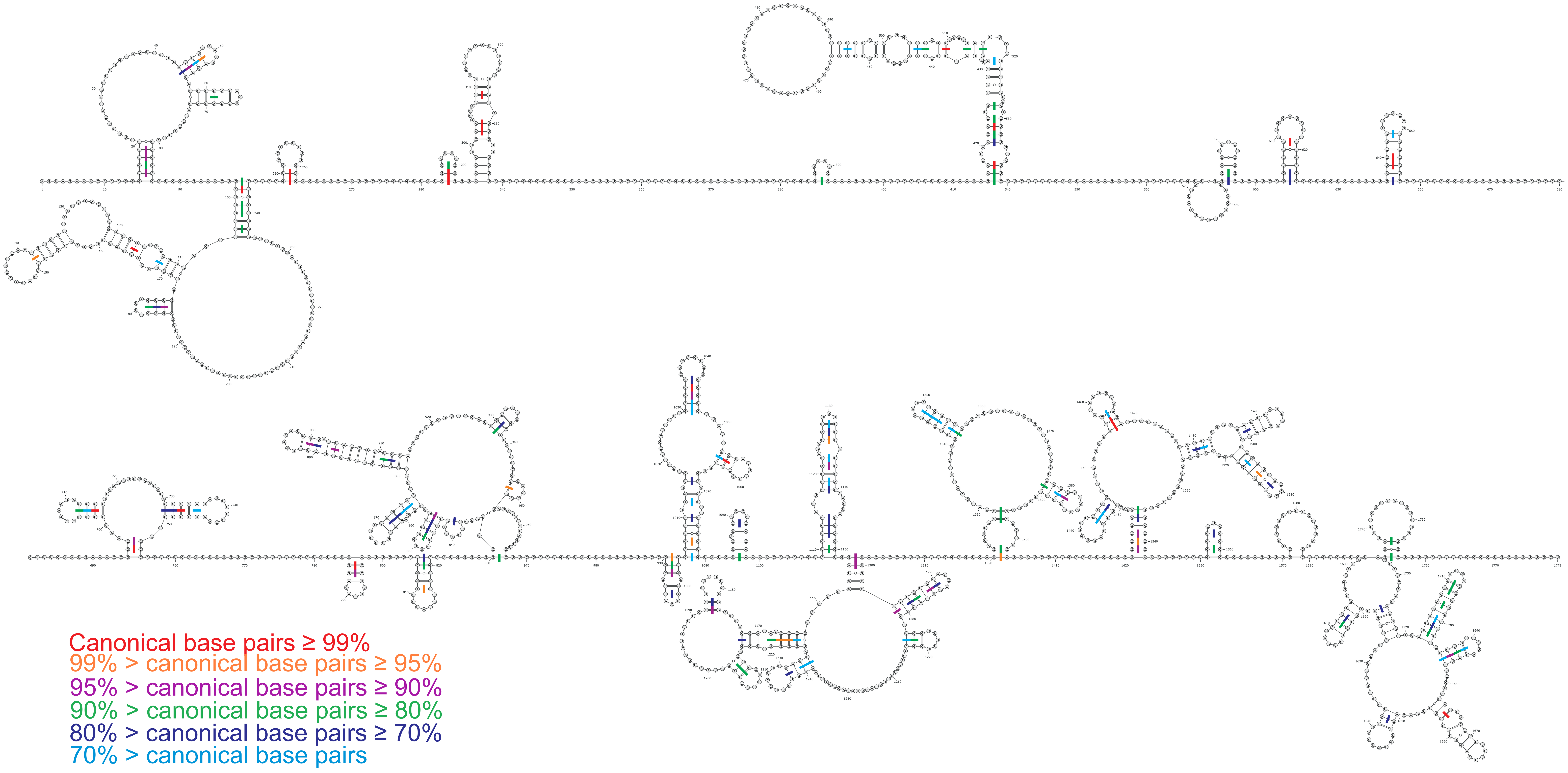


vRNA4 *in virio* local MEA

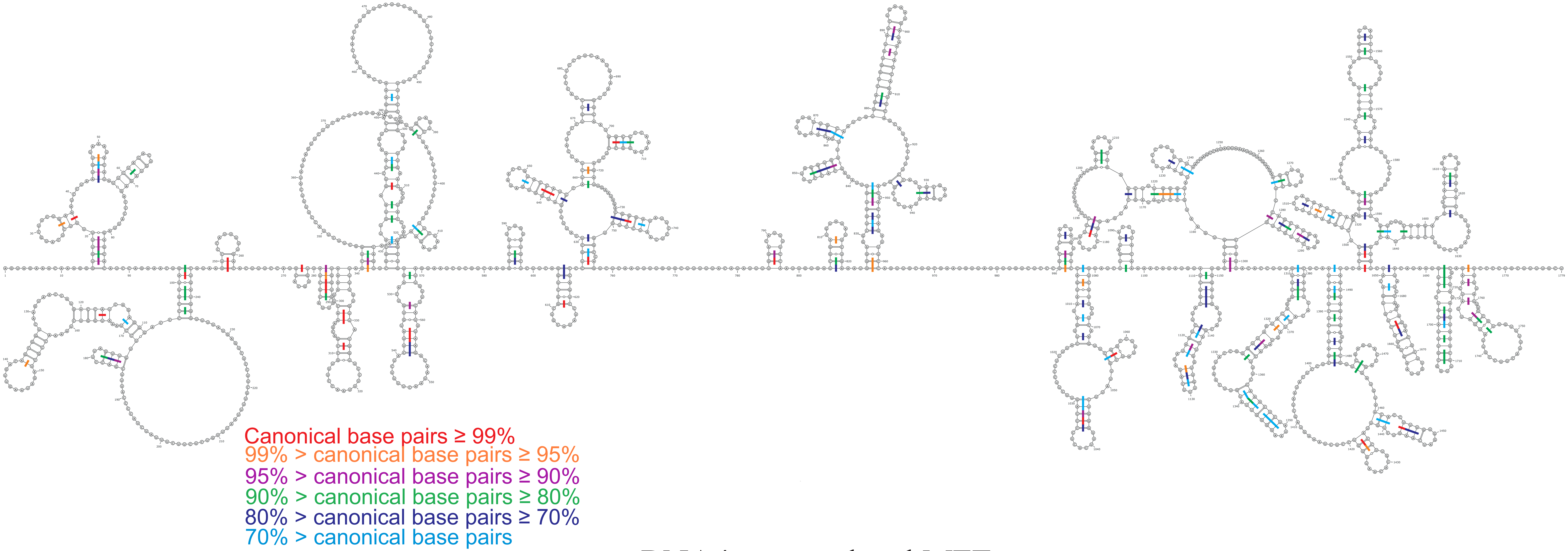


vRNA4 *in virio* local MFE

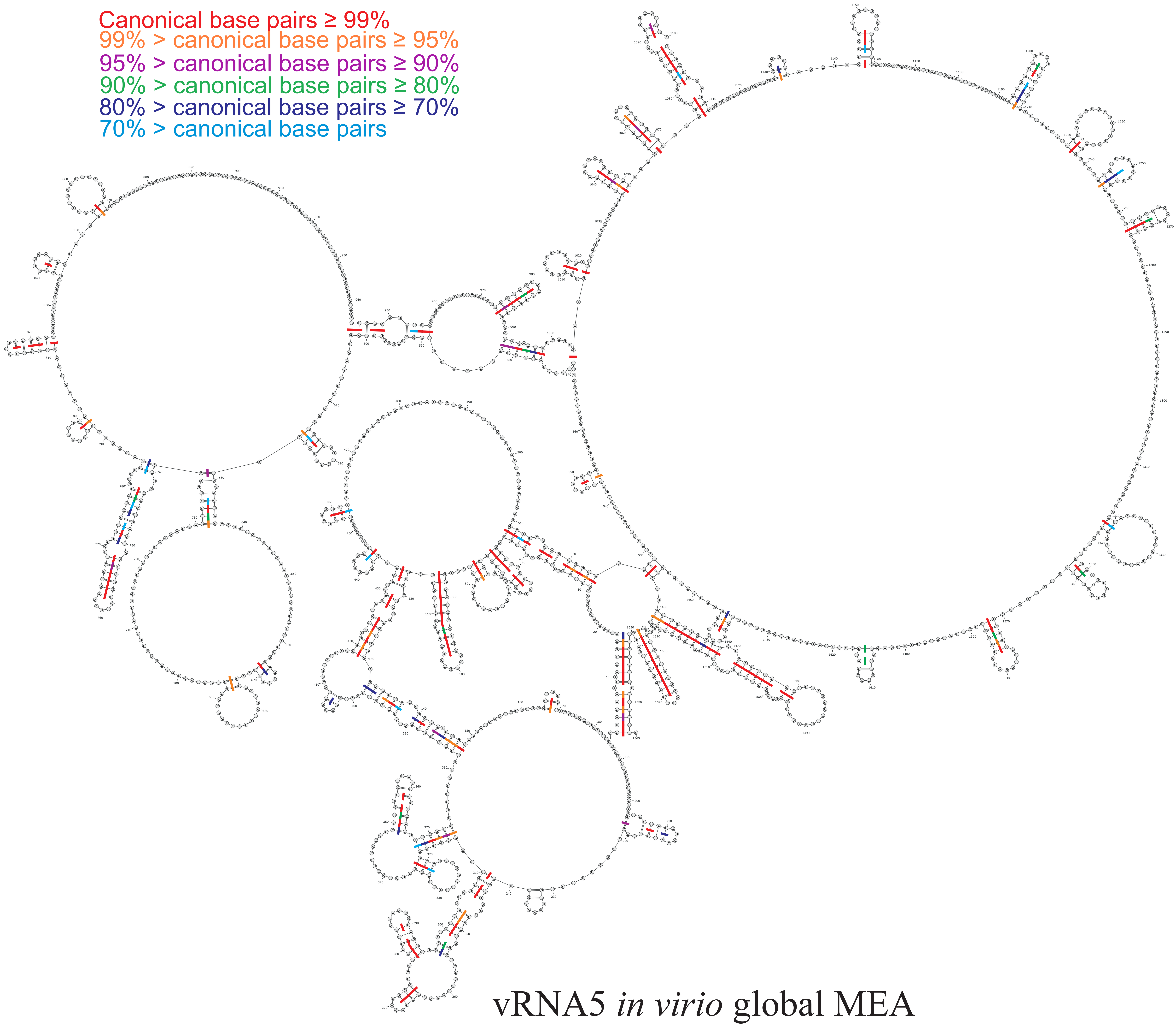


vRNA5 *in virio* global MEA

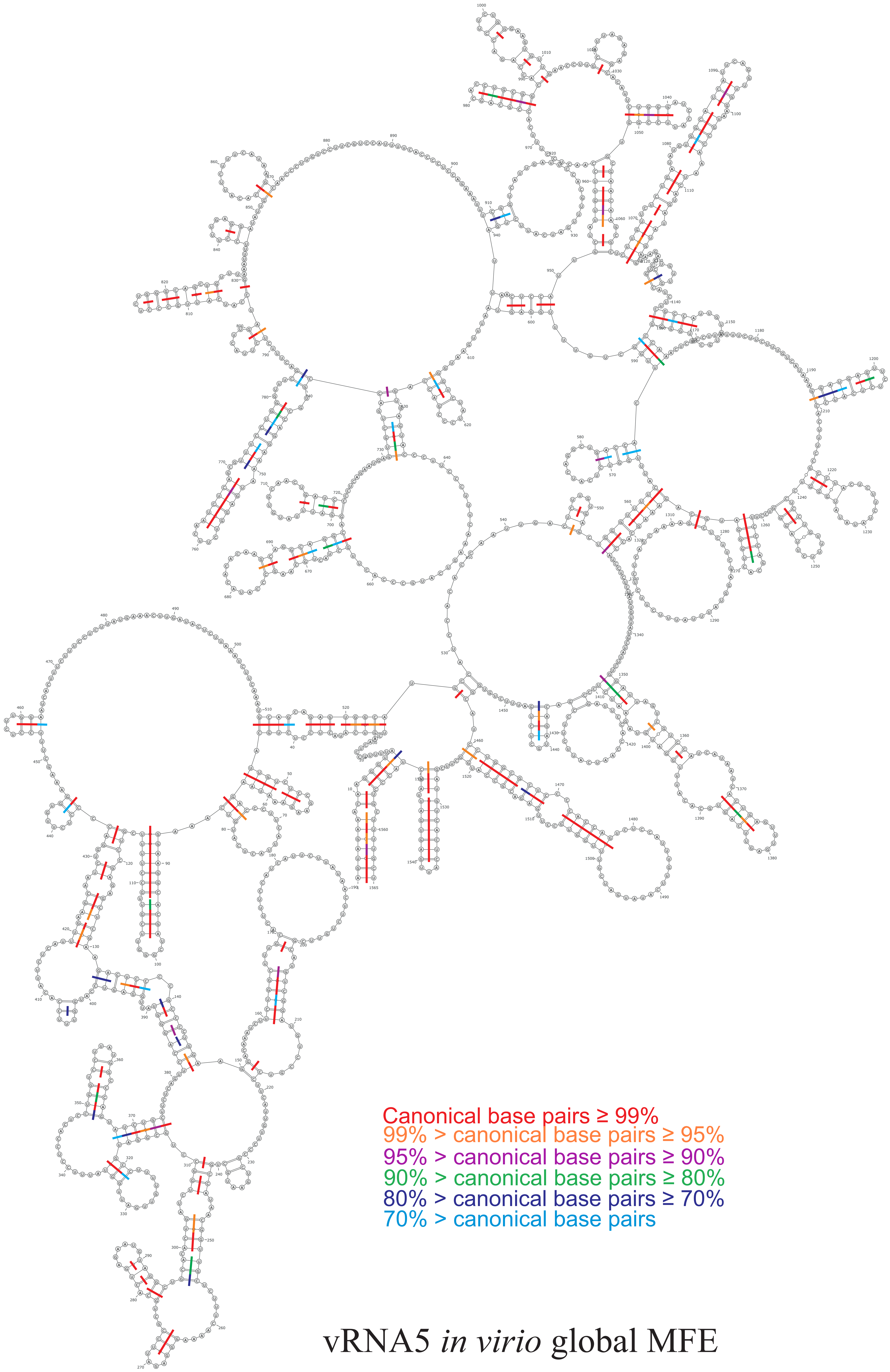


vRNA5 *in virio* global MFE

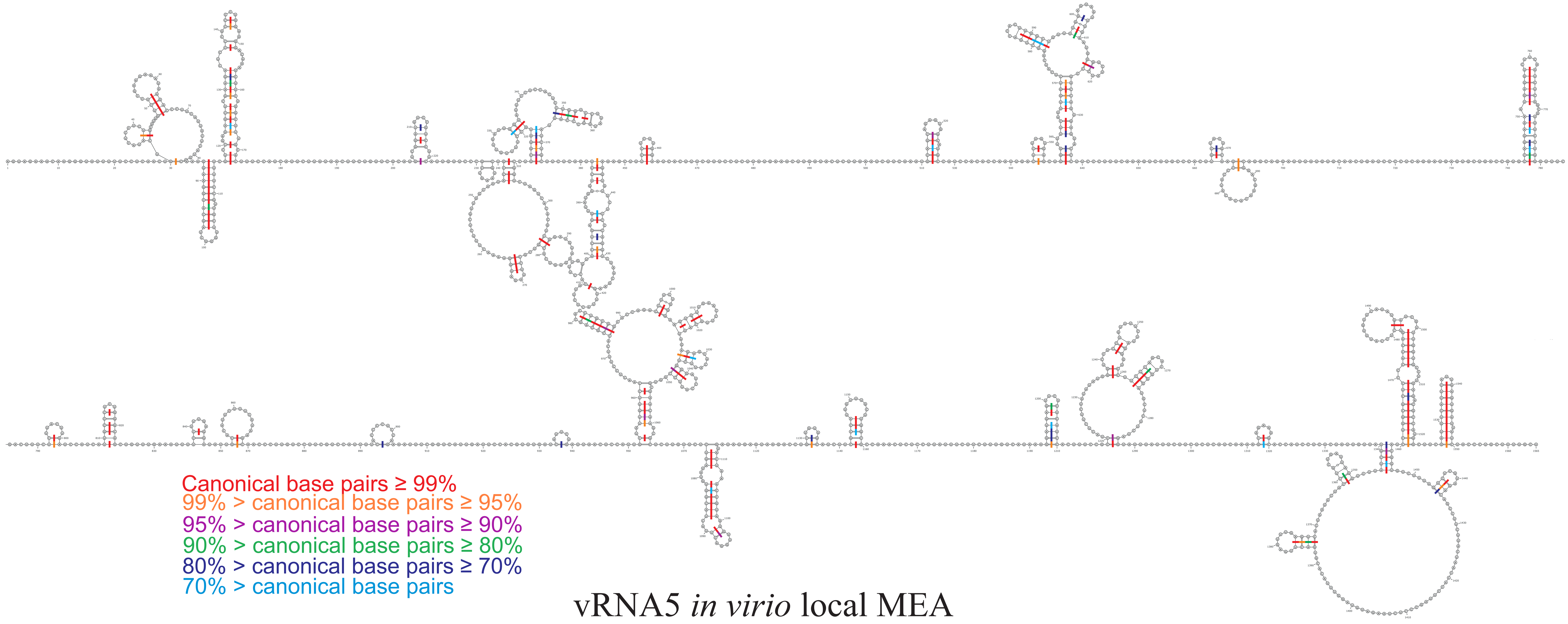


vRNA5 *in virio* local MEA

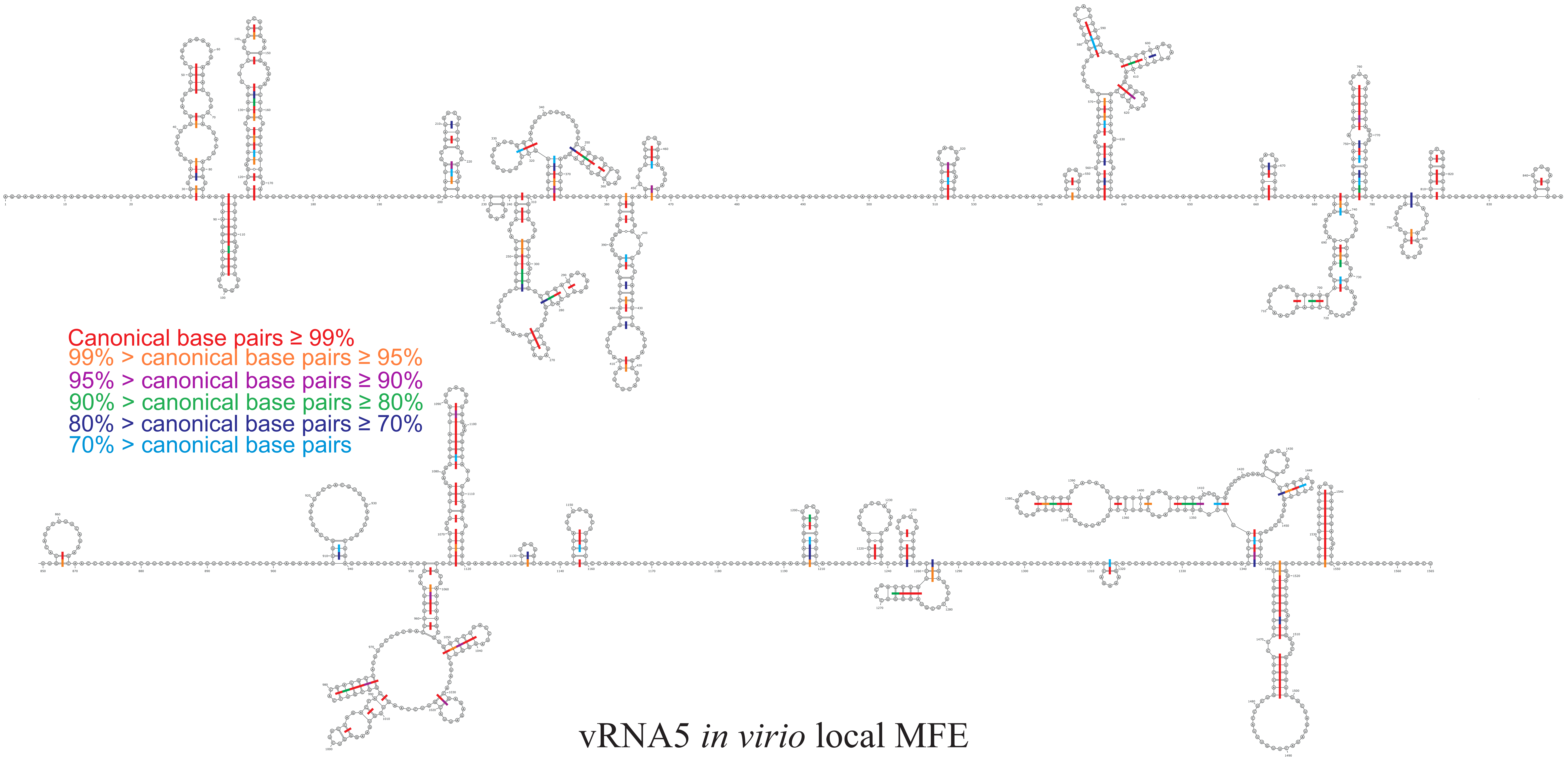


vRNA5 *in virio* local MFE

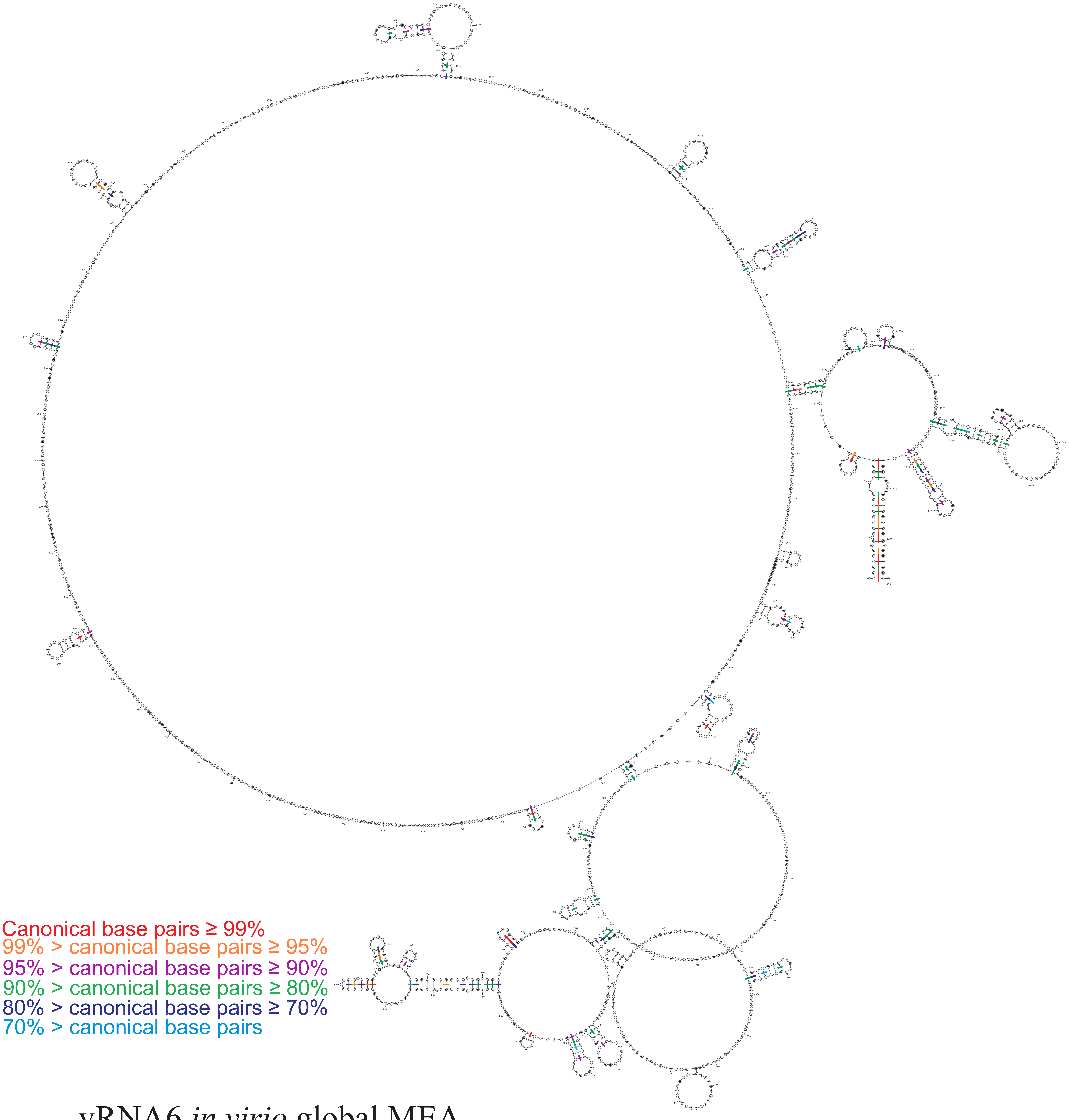


vRNA6 *in virio* global MEA

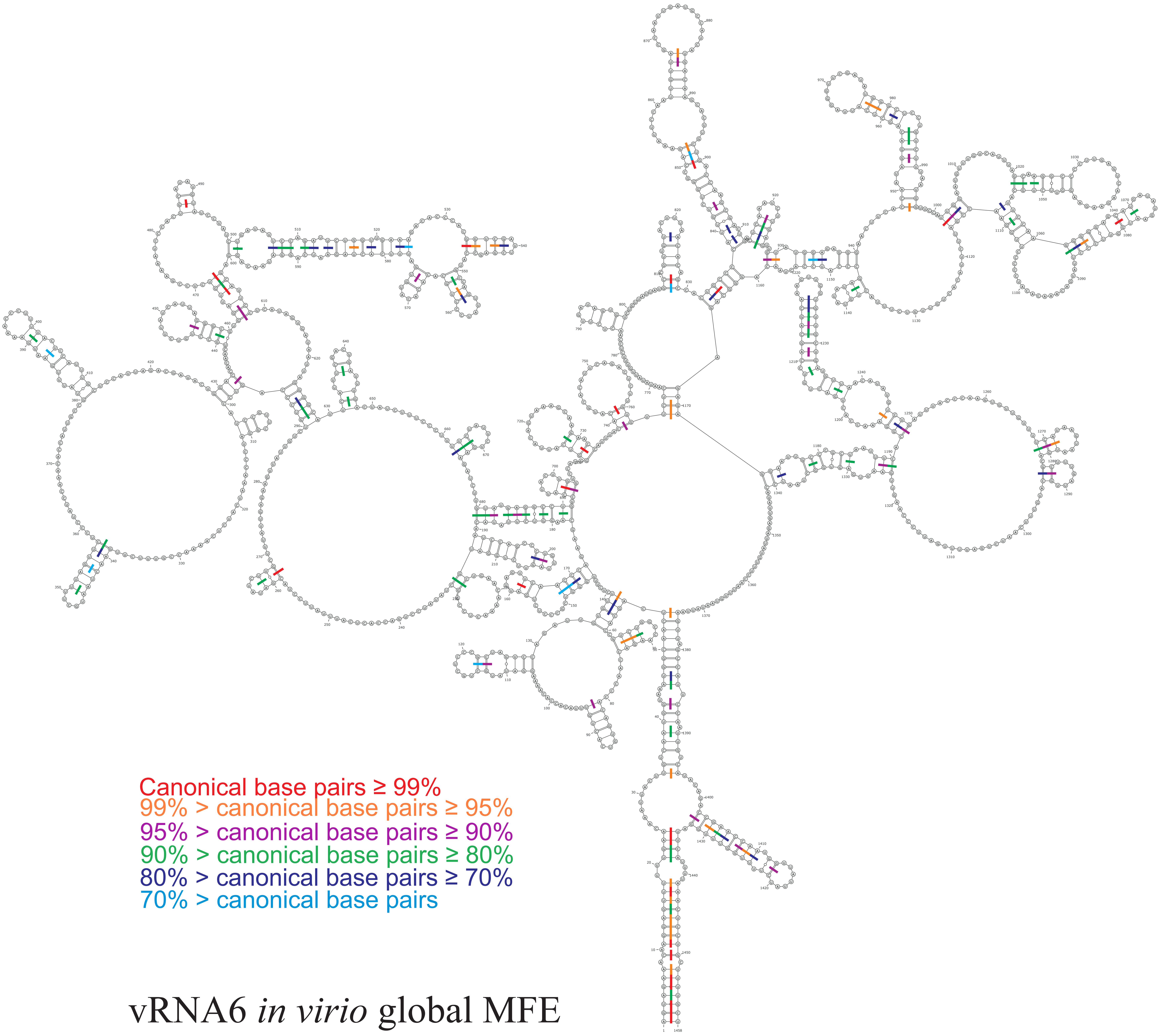


vRNA6 *in virio* global MFE

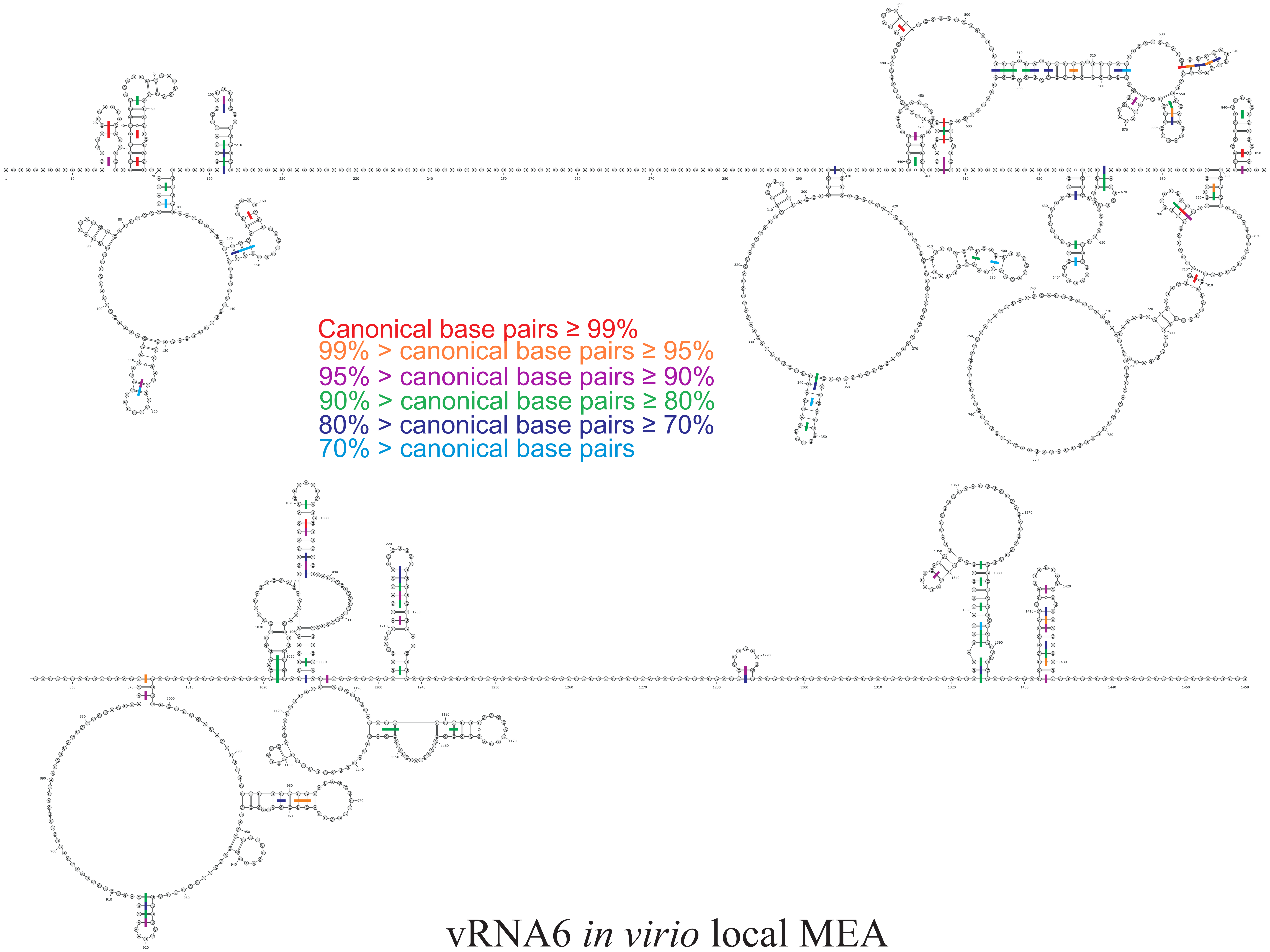


vRNA6 *in virio* local MEA

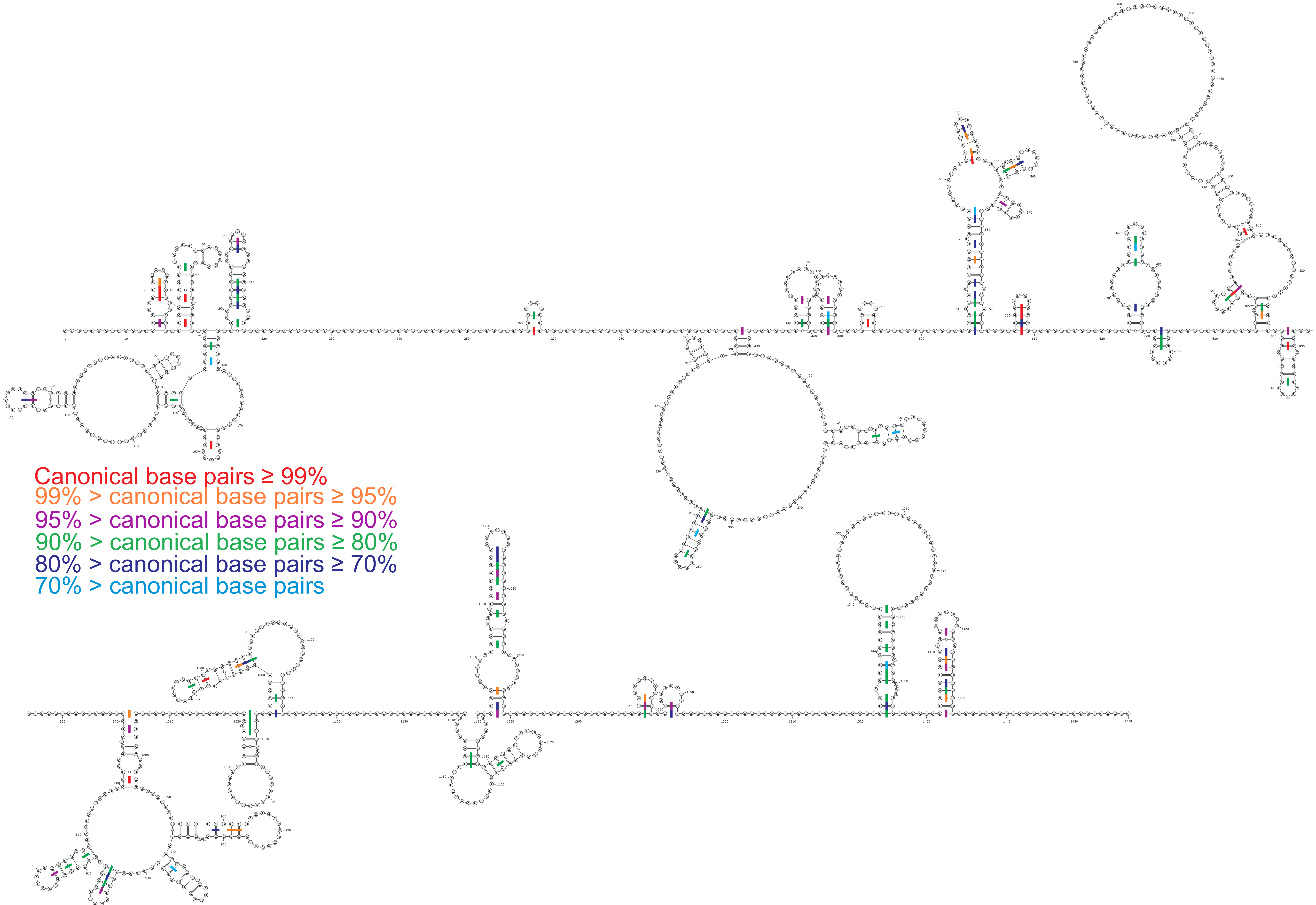


vRNA6 *in virio* local MFE

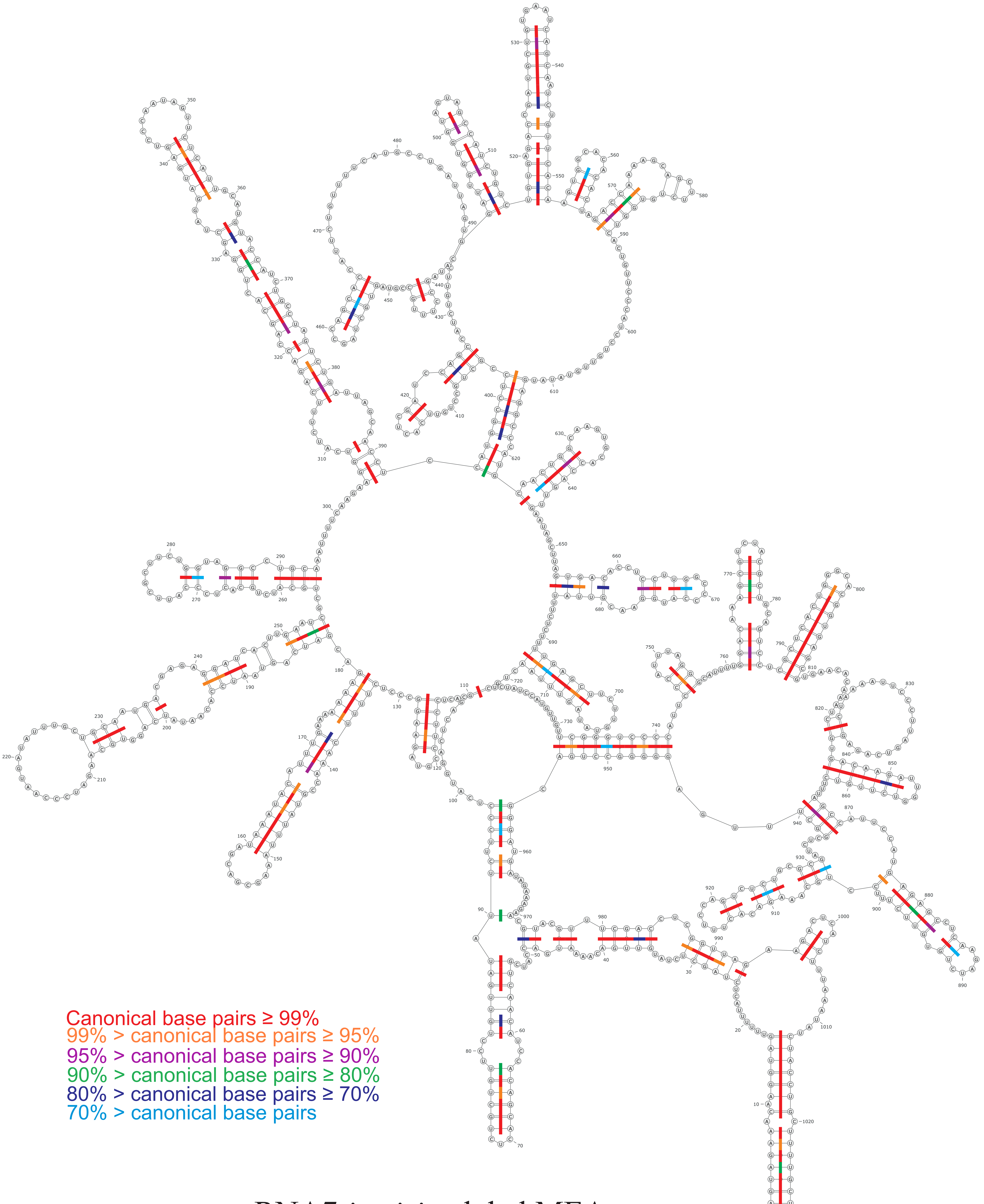


vRNA7 *in virio* global MEA

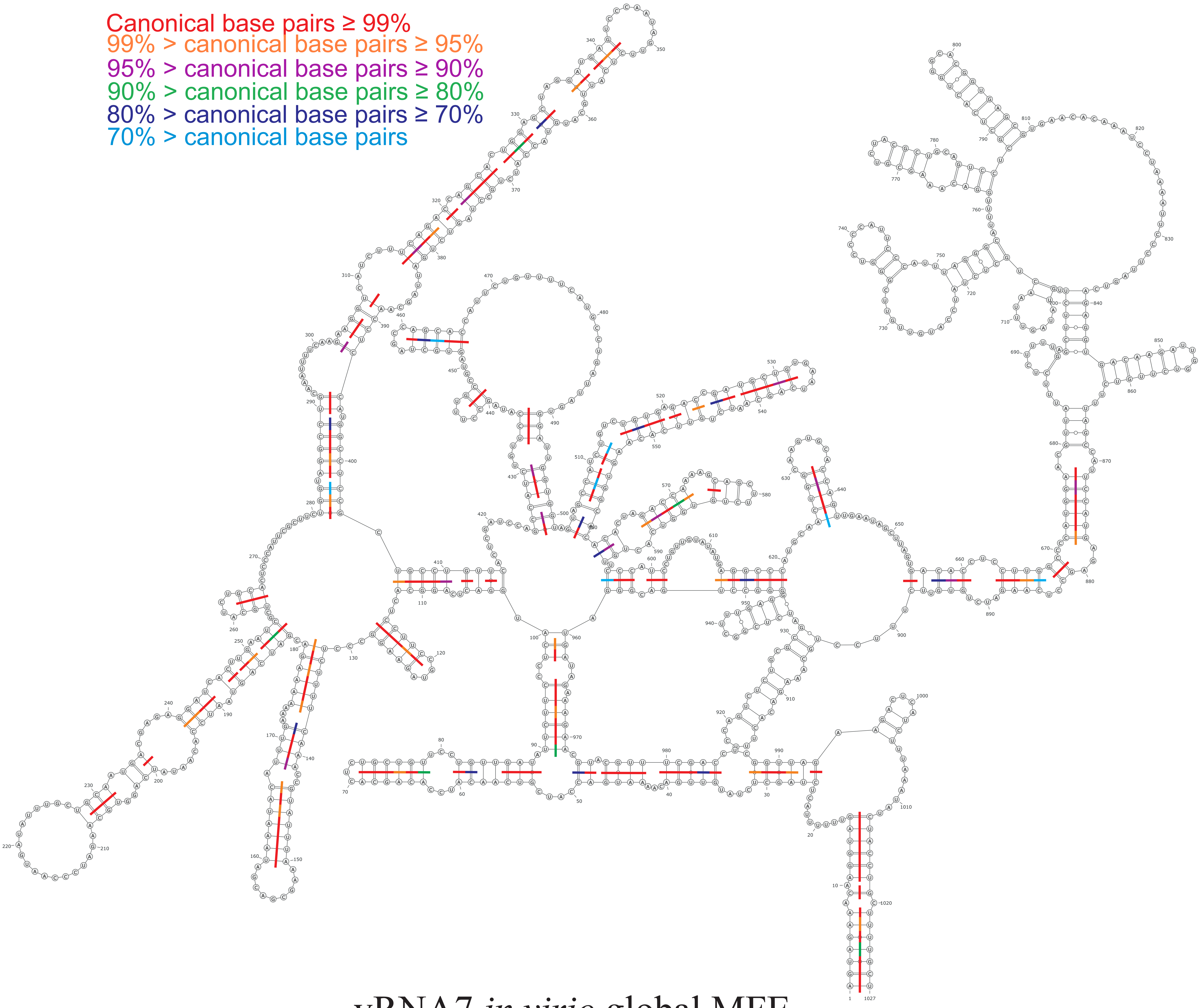


vRNA7 *in virio* global MFE

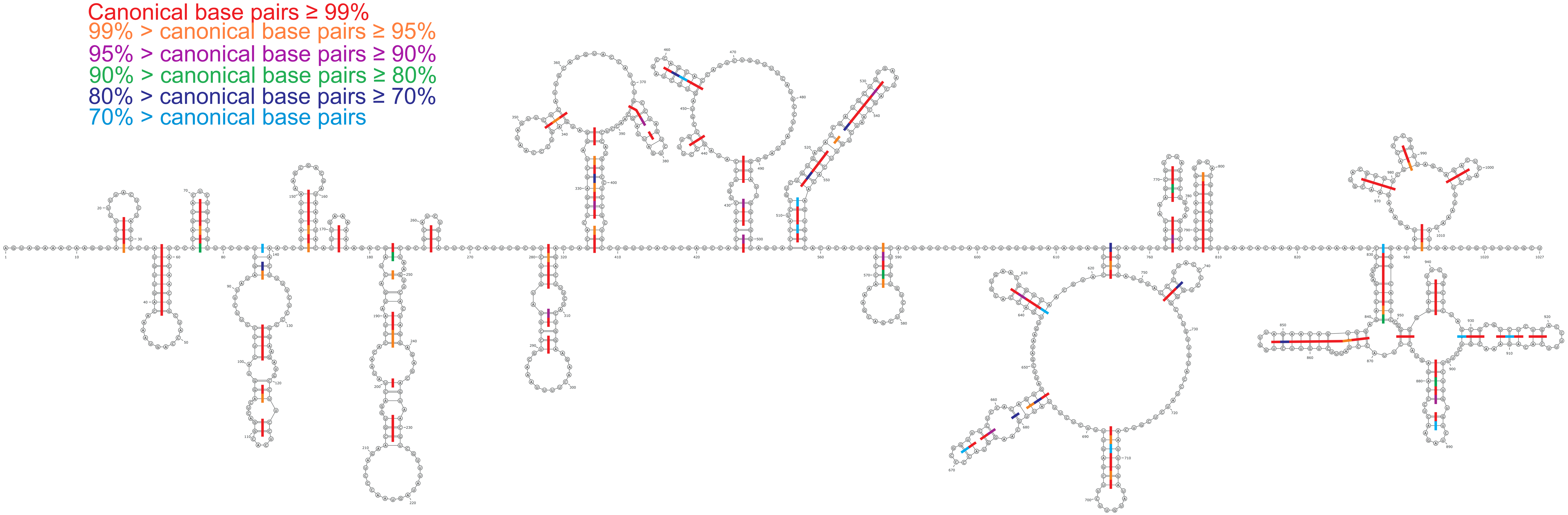


vRNA7 *in virio* local MEA

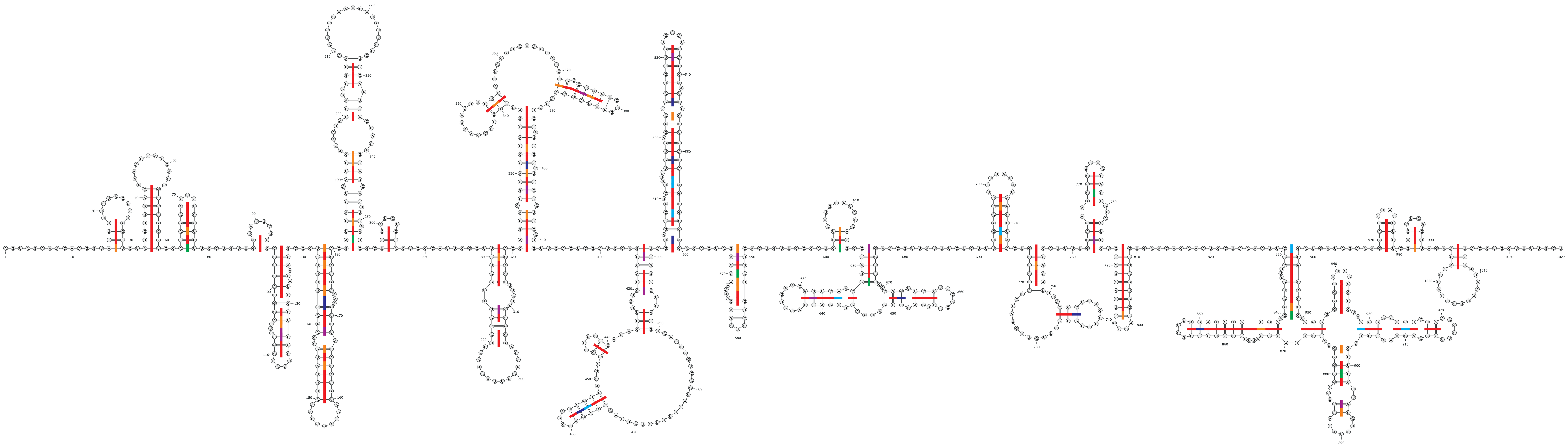

Canonical base pairs ≥ 99%
99% > canonical base pairs ≥ 95%
95% > canonical base pairs ≥ 90%
90% > canonical base pairs ≥ 80%
80% > canonical base pairs ≥ 70%
70% > canonical base pairs

vRNA7 *in virio* local MFE

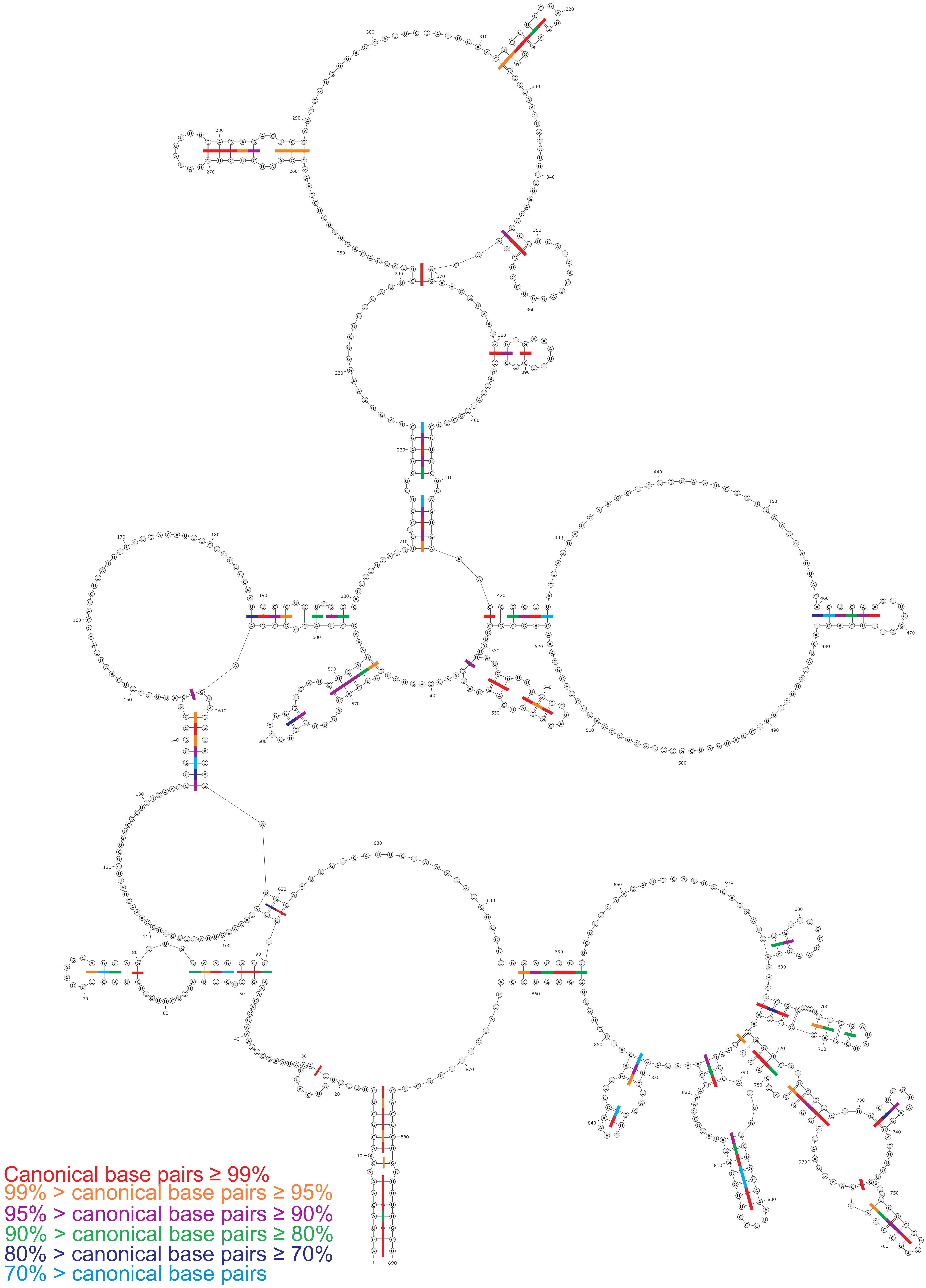


vRNA8 *in virio* global MEA

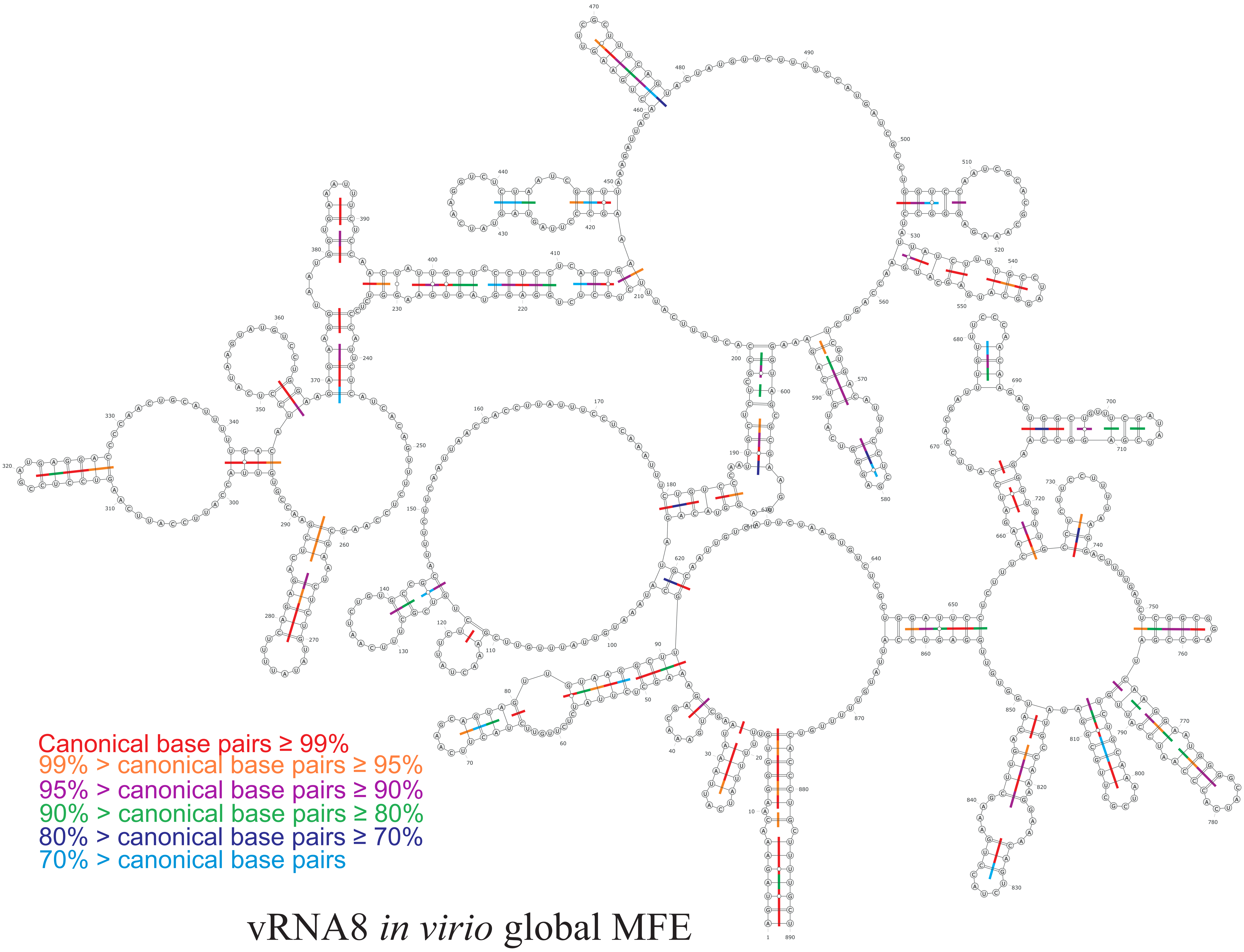


vRNA8 *in virio* global MFE

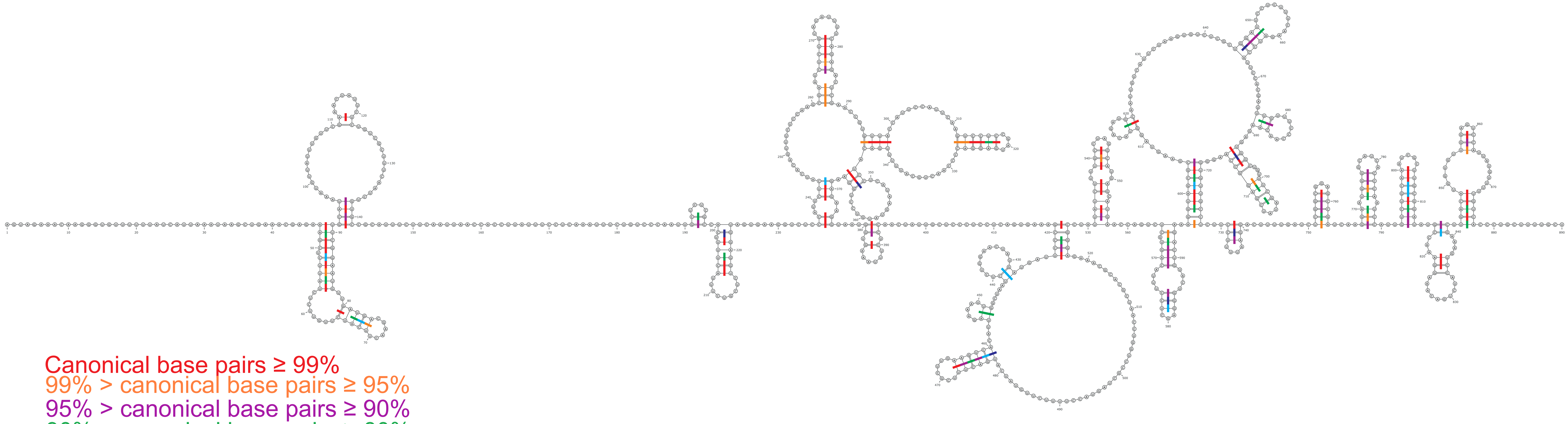

Canonical base pairs ≥ 99%
99% > canonical base pairs ≥ 95%
95% > canonical base pairs ≥ 90%
90% > canonical base pairs ≥ 80%
80% > canonical base pairs ≥ 70%
70% > canonical base pairs

vRNA8 *in virio* local MEA

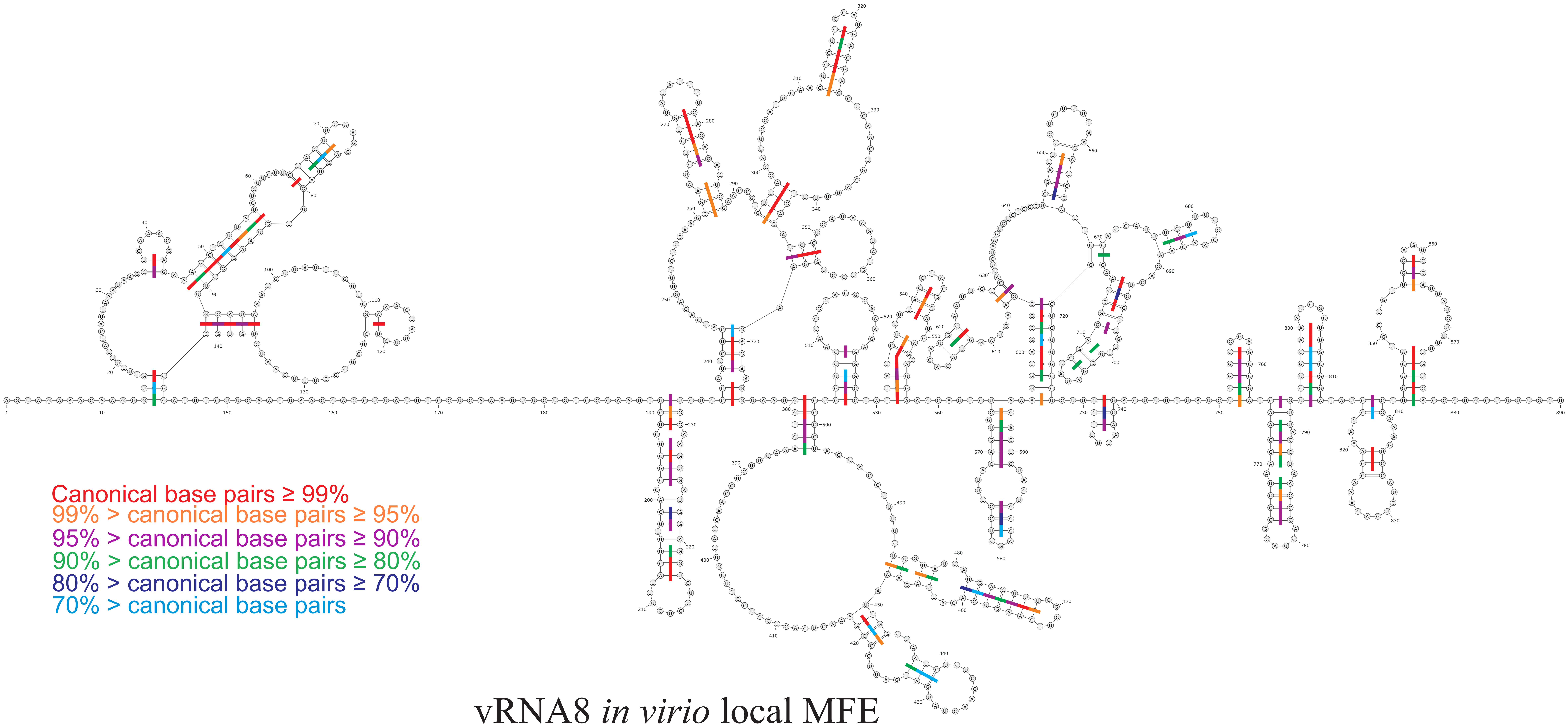


vRNA8 *in virio* local MFE

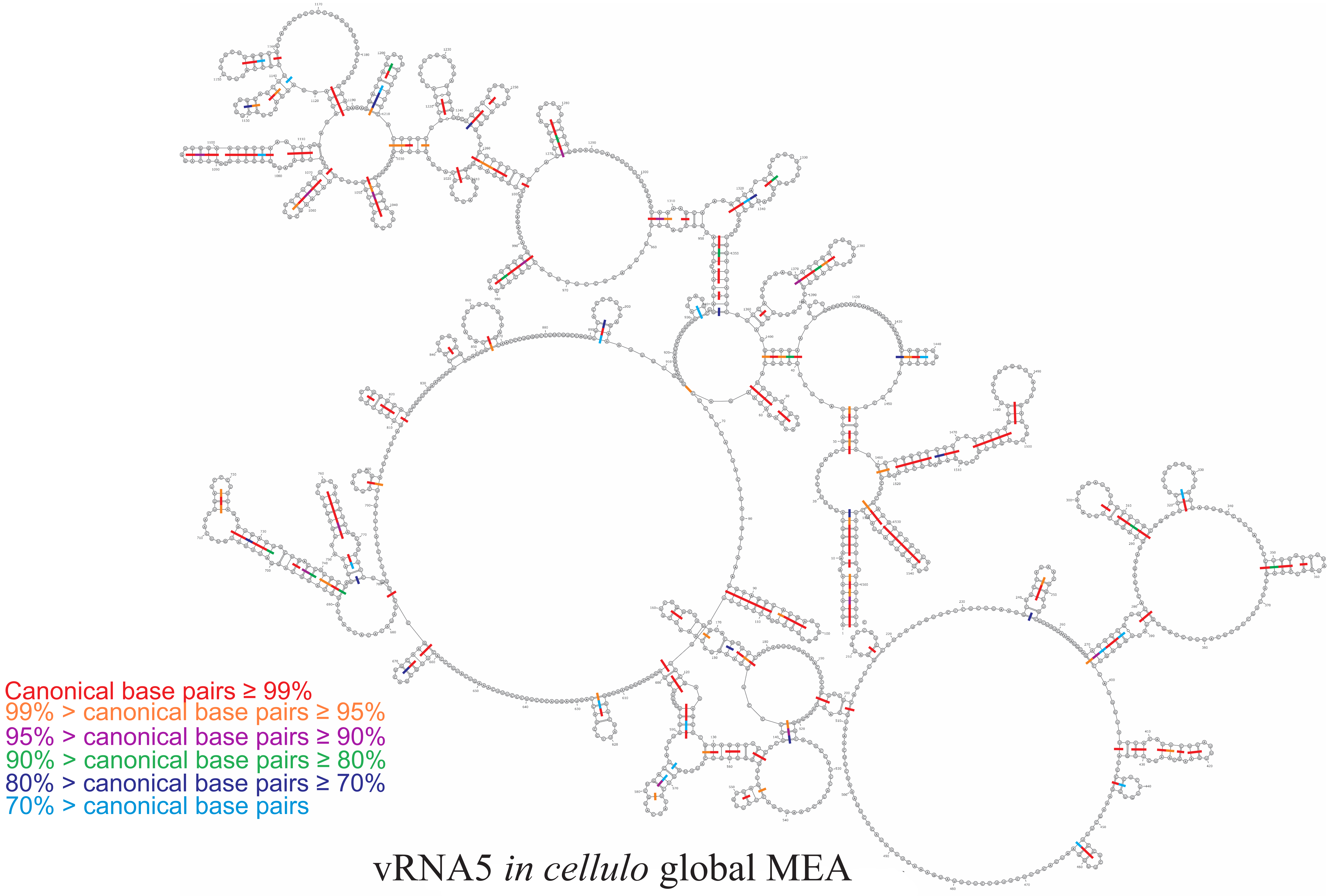


vRNA5 *in cellulo* global MEA

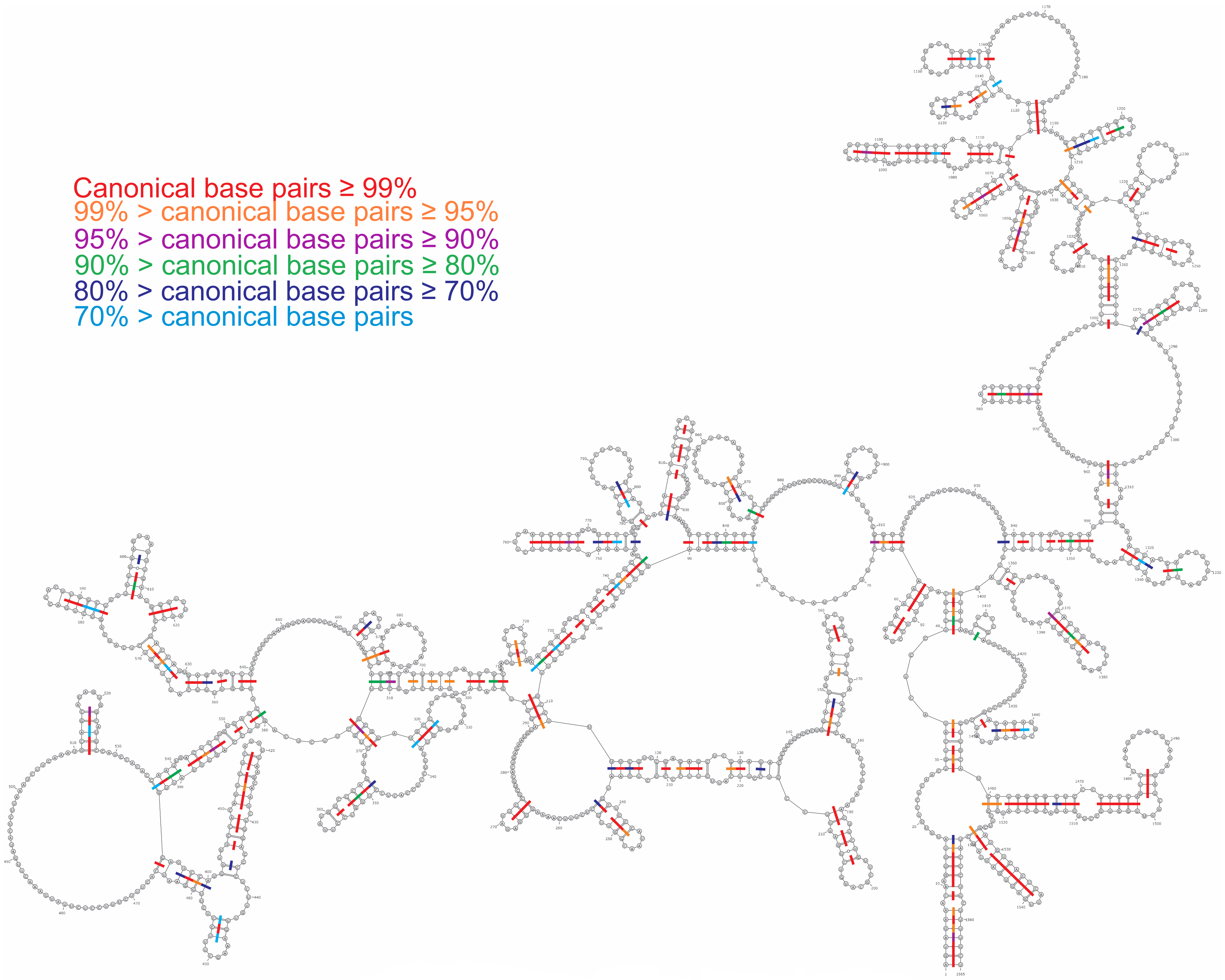


vRNA5 *in cellulo* global MFE

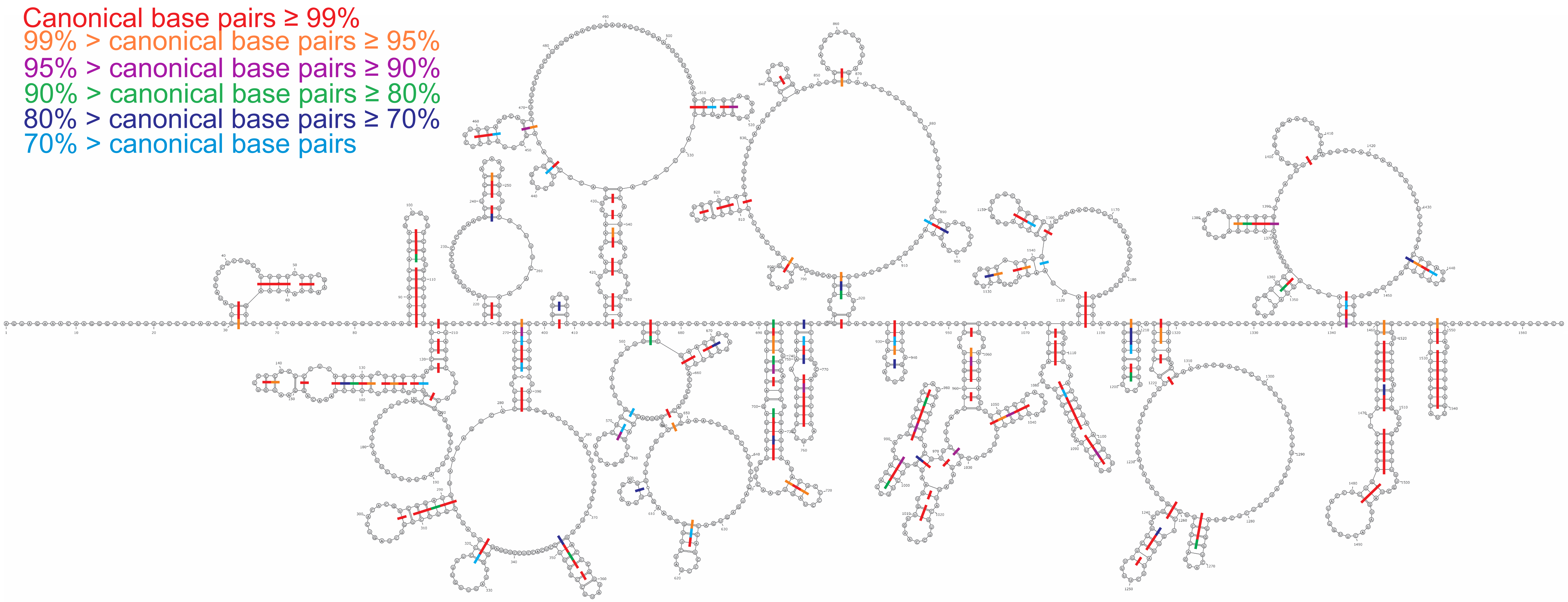


vRNA5 *in cellulo* local MEA

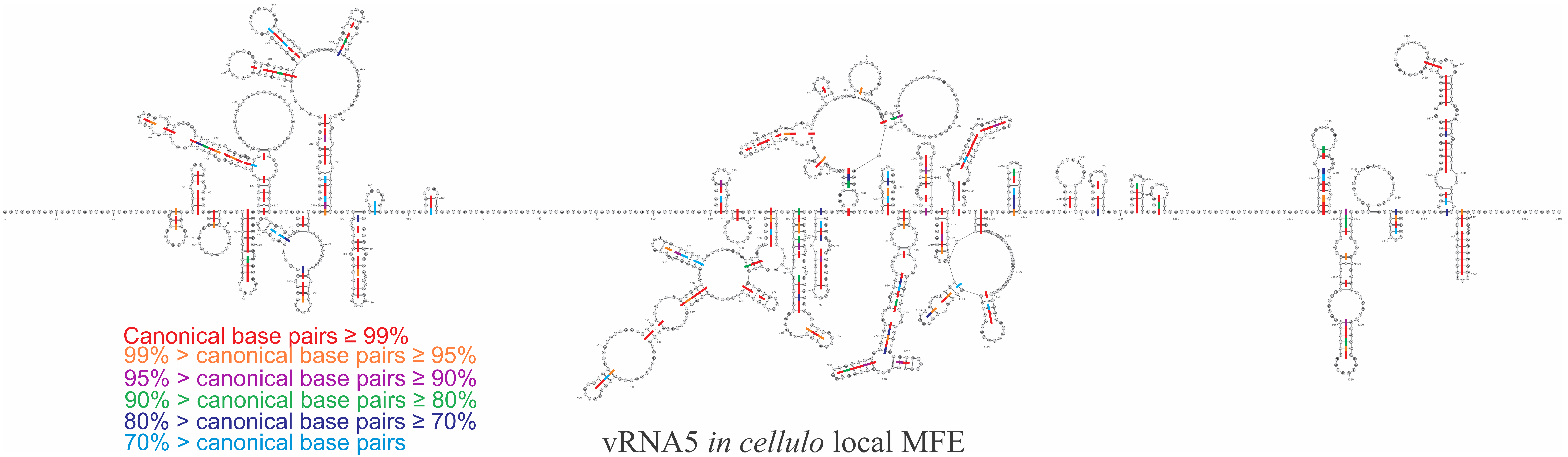


vRNA5 *in cellulo* local MFE

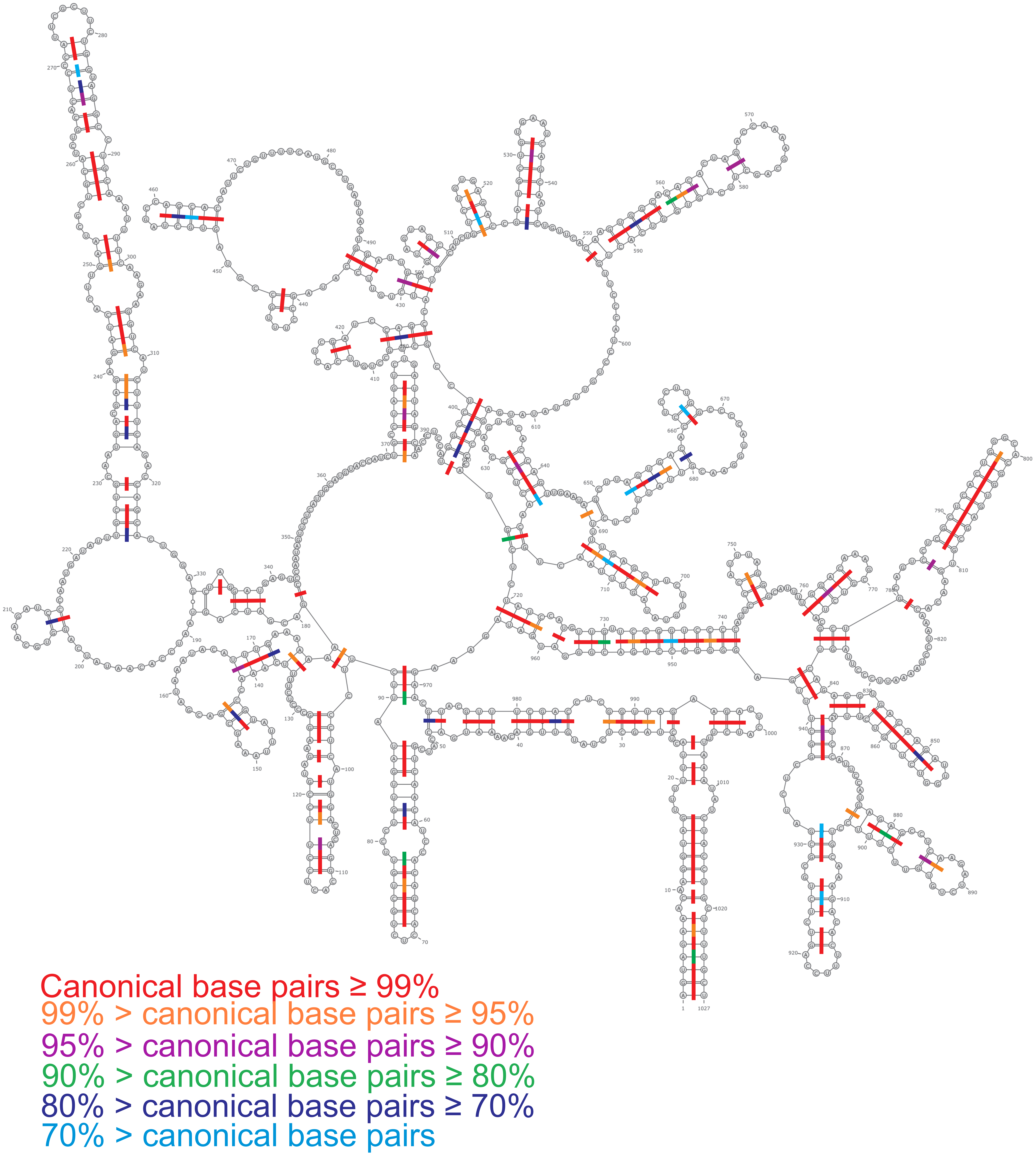


vRNA7 *in cellulo* global MEA

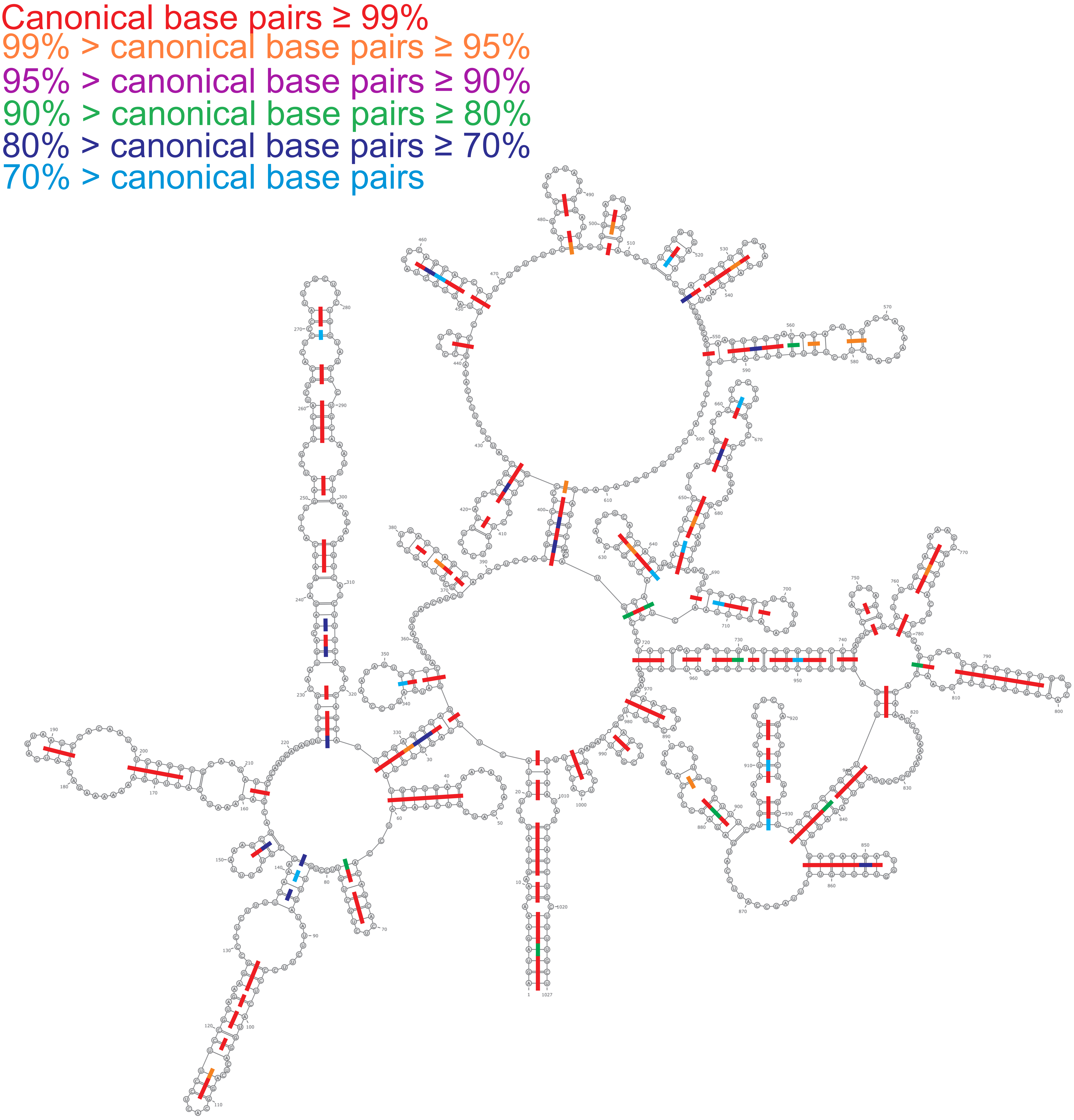


vRNA7 *in cellulo* global MFE

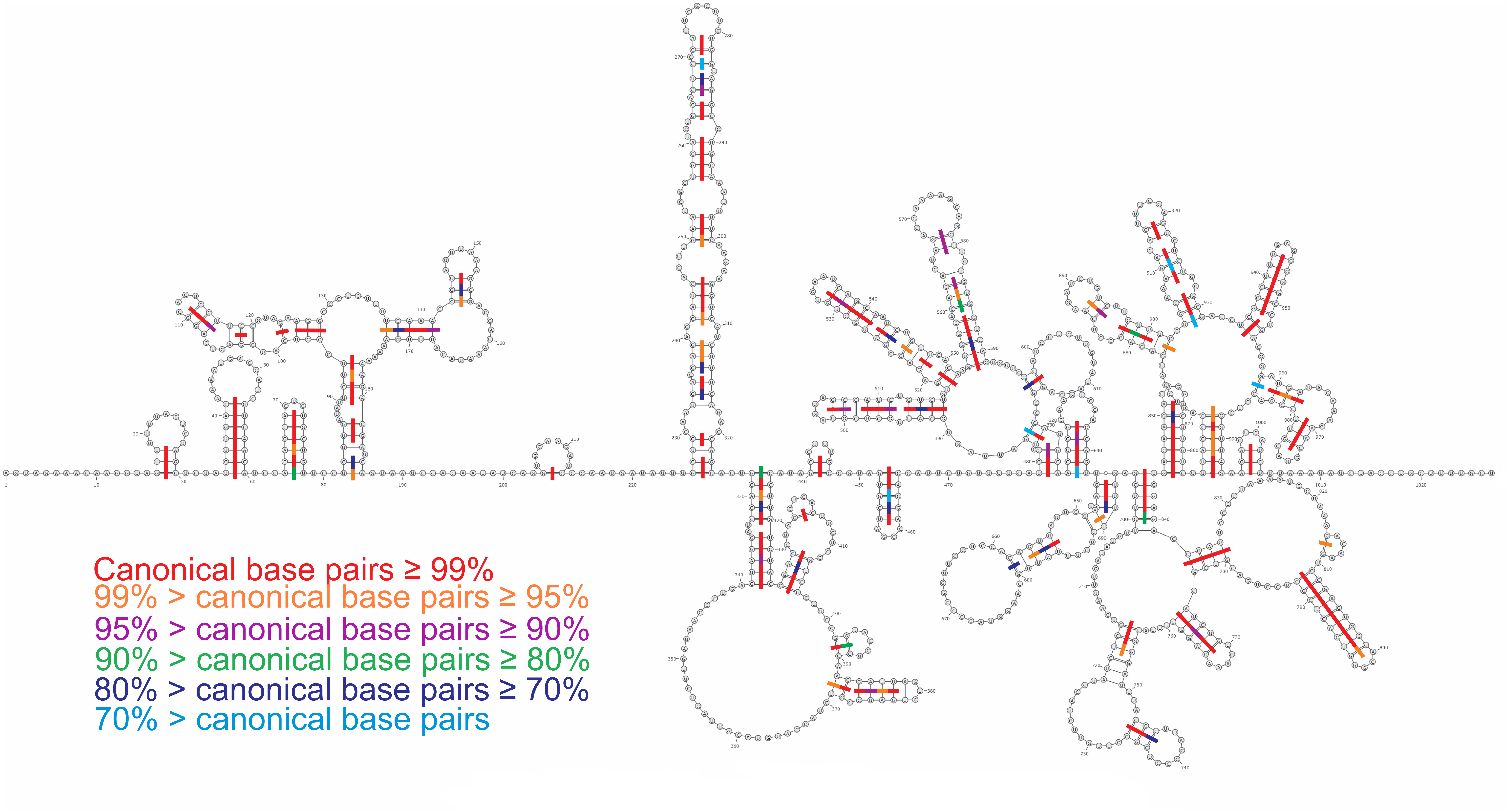


vRNA7 *in cellulo* local MFE

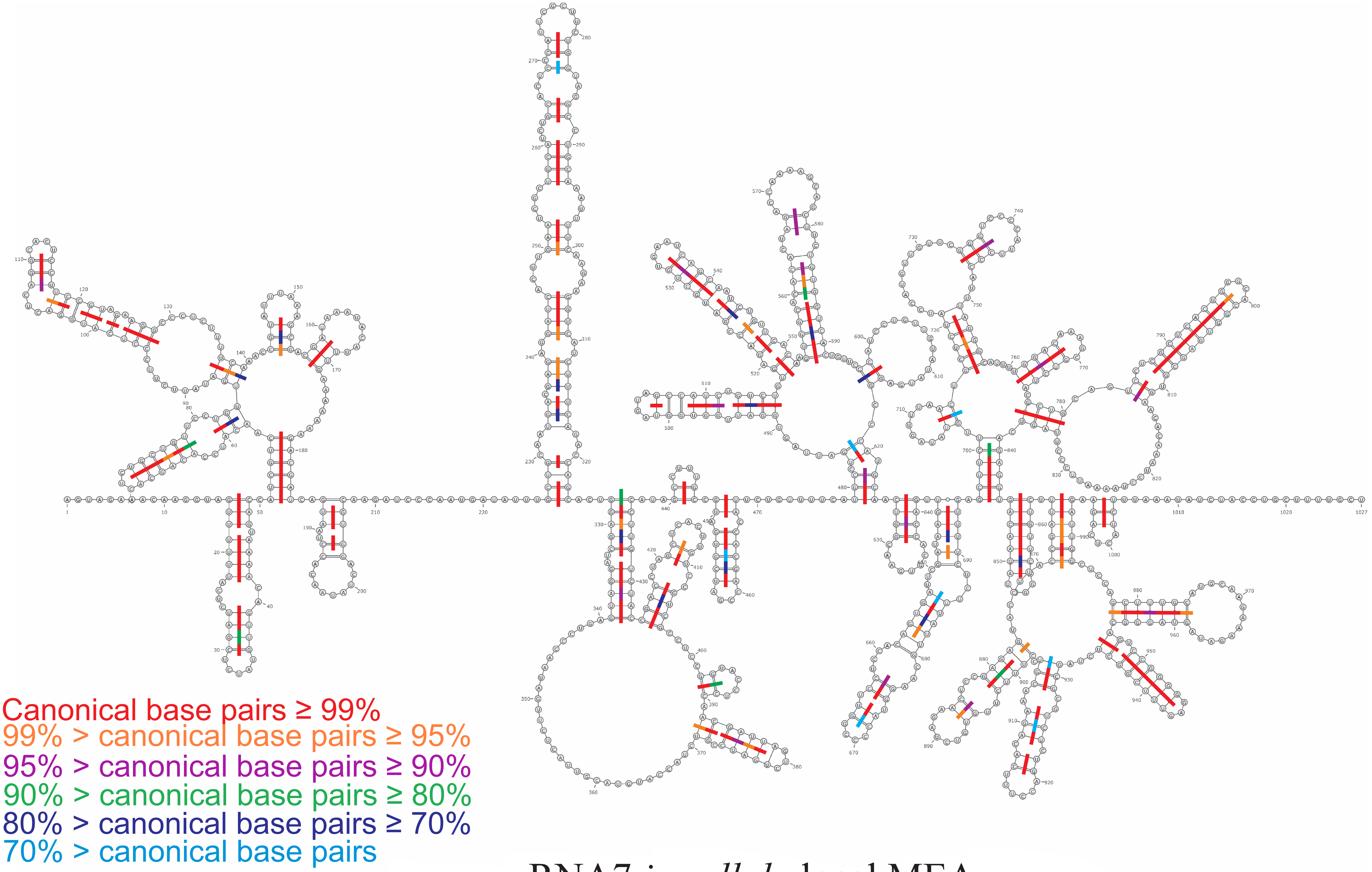


vRNA7 *in cellulo* local MEA

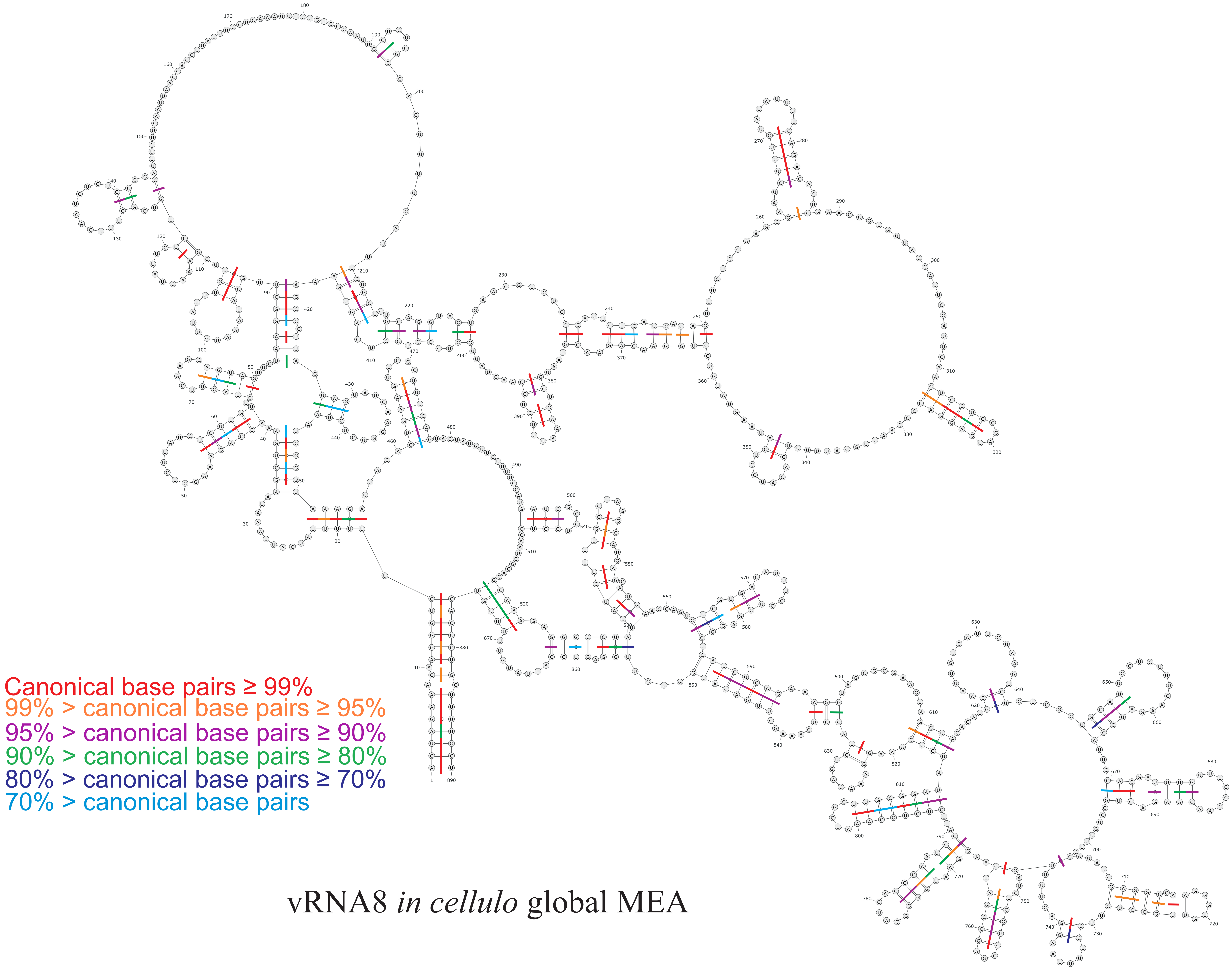


vRNA8 *in cellulo* global MEA

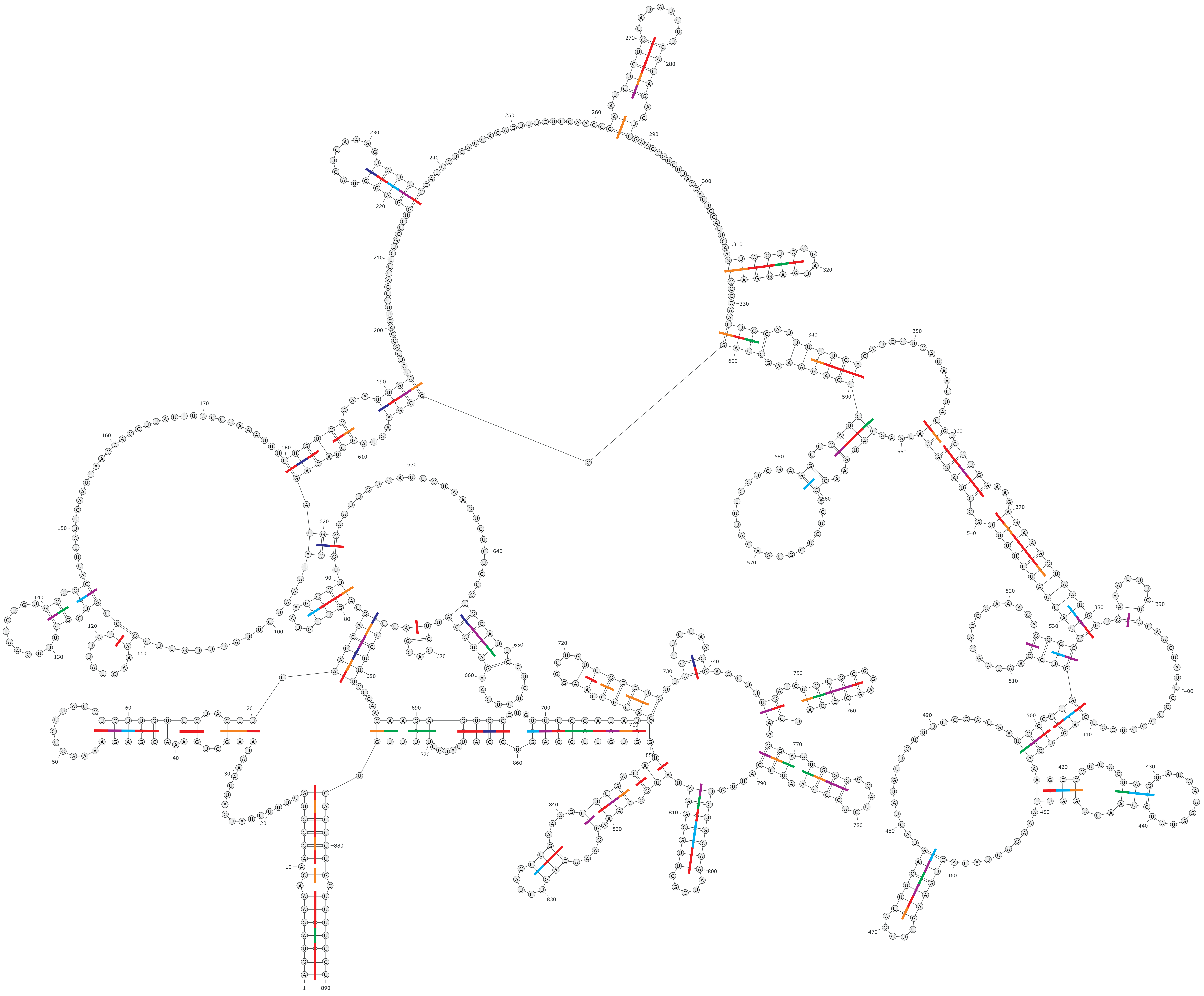

Canonical base pairs ≥ 99%
99% > canonical base pairs ≥ 95%
95% > canonical base pairs ≥ 90%
90% > canonical base pairs ≥ 80%
80% > canonical base pairs ≥ 70%
70% > canonical base pairs

vRNA8 *in cellulo* global MFE

Canonical base pairs ≥ 99%
99% > canonical base pairs ≥ 95%
95% > canonical base pairs ≥ 90%
90% > canonical base pairs ≥ 80%
80% > canonical base pairs ≥ 70%
70% > canonical base pairs

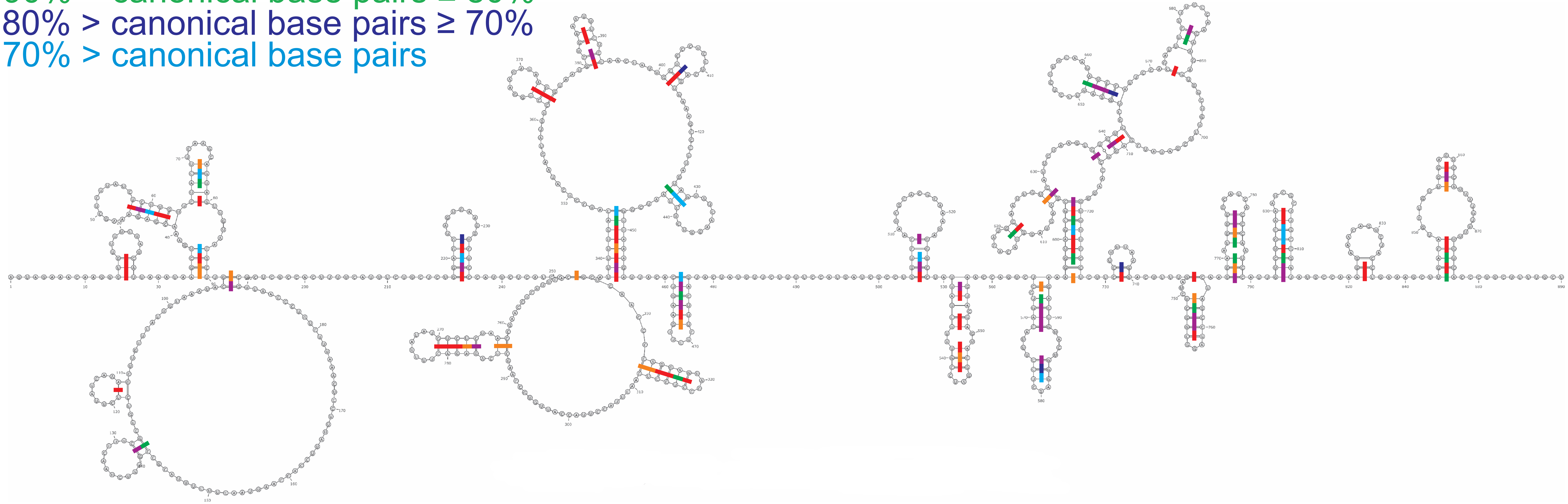

vRNA8 *in cellulo* local MEA

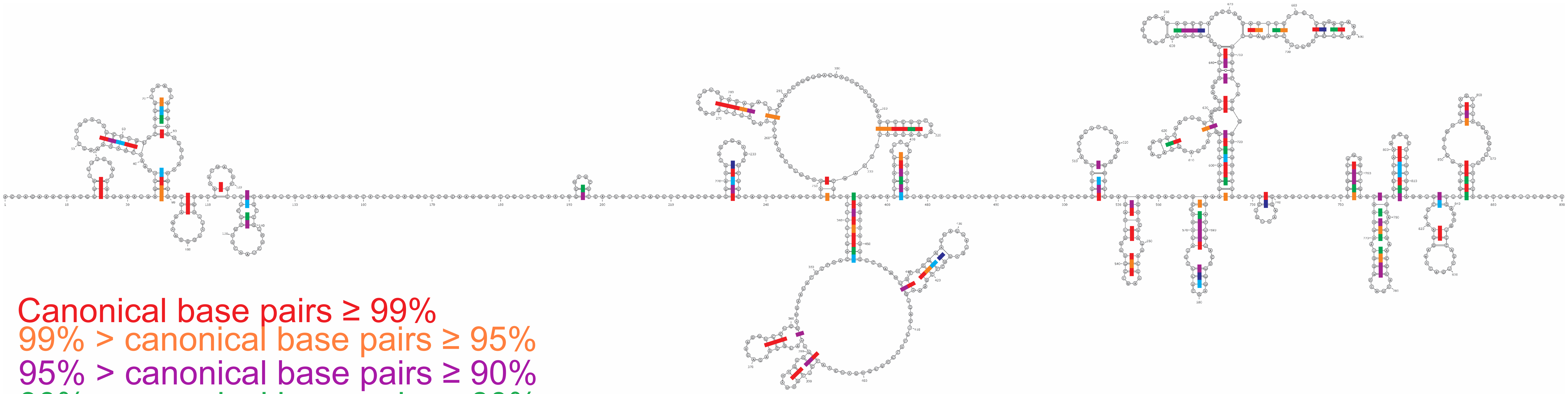

Canonical base pairs ≥ 99%
99% > canonical base pairs ≥ 95%
95% > canonical base pairs ≥ 90%
90% > canonical base pairs ≥ 80%
80% > canonical base pairs ≥ 70%
70% > canonical base pairs

vRNA8 *in cellulo* local MFE
